# Straightforward synthesis of Solomon-red-BAPTA as NIR fluorescent sensors *via* metal-free oxidative coupling

**DOI:** 10.1039/d6ra01589a

**Published:** 2026-06-01

**Authors:** Gergő Riszter, László Forgách, Zoltán Mucsi, Laura Molnár, Dóra Bogdán, Fatemeh Heydari, Domokos Máthé, István Mándity, Ágnes Gömöry, Zoltán Kaleta

**Affiliations:** a Institute of Organic Chemistry, Semmelweis University Hőgyes Endre Street 7 H-1092 Budapest Hungary kaleta.zoltan@semmelweis.hu; b PROGRESSIO Engineering Bureau Ltd Muhar u. 54 H-1028 Budapest Hungary; c Artificial Transporters Research Group, Institute of Materials and Environmental Chemistry, Research Centre for Natural Sciences, HUN-REN Hungarian Research Network Magyar Tudósok Körútja 2 Budapest H-1117 Hungary; d Department of Biophysics, Semmelweis University Tűzoltó utca 37-47 Budapest 1094 Hungary; e In Vivo Imaging Advanced Core Facility, Hungarian Center of Excellence for Molecular Medicine (HCEMM) Tűzoltó Utca 37-47 Budapest 1094 Hungary; f Department of Nuclear Medicine, Medical Imaging Centre, Semmelweis University Üllői út 78b Budapest 1083 Hungary; g BrainVisionCenter 1094 43-45. Liliom Str. Budapest Hungary; h Institute of Chemistry, University of Miskolc 3515 Miskolc-Egyetemváros A/2 Building Hungary; i CROmed Translational Research Centers 37-47 Tűzoltó Street Budapest 1094 Hungary; j Centre for Pharmacology and Drug Research & Development, Semmelweis University Budapest Hungary; k MS Proteomics Research Group, Institute of Organic Chemistry, Research Centre for Natural Sciences, HUN-REN Hungarian Research Network Magyar Tudósok Körútja 2 Budapest H-1117 Hungary

## Abstract

An effective, near-infrared (NIR) fluorescent Ca^2+^ sensor, Solomon-red-BAPTA, featuring a dioxothioxanthene moiety was developed for microscopy. A rapid and operationally simple synthetic route is described. The key step involves a room-temperature DDQ-mediated cross-dehydrogenative coupling (CDC) of the fluorescent precursor and BAPTA followed by a one-pot oxidation. The sensor exhibited excellent photophysical characteristics including a 160-fold fluorescence enhancement with excitation and emission maxima at 712 nm and 736 nm, respectively, suggesting high tissue-penetrating capacity. Based on the experimental data, the structure–property relationship was elucidated and further supported by DFT calculations, particularly with respect to the photoinduced electron transfer (PET)-based fluorescence turn-on mechanism.

## Abstract 2

A room-temperature, DDQ-mediated oxidative coupling enables the straightforward synthesis of a dioxothioxanthene-based NIR sensor, bypassing cumbersome traditional multi-step pathways and achieving a 160-fold fluorescence turn-on.

## Graphical abstract

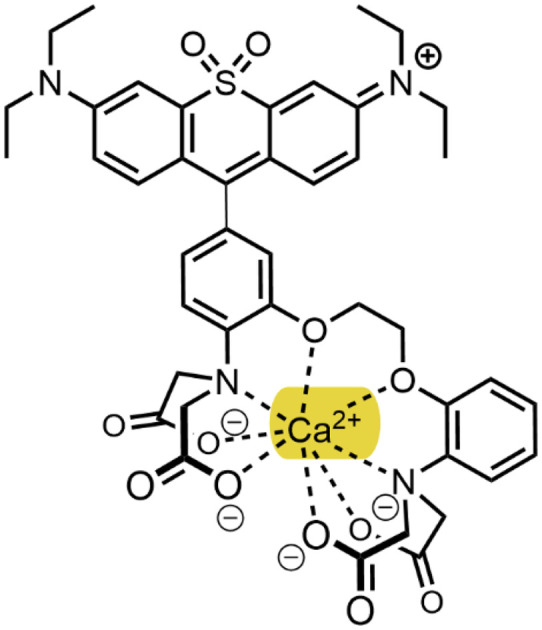

## Introduction

Fluorescent imaging is widely used in biological research for the study of living tissues and biochemical processes with single- or multi-photon irradiation.^1–3^ Among these approaches, *in vivo* Ca^2+^ imaging plays a central role, as it enables the identification of networks of actively firing nerve cells within living animals.^4,5^ For this purpose, numerous fluorescent sensors coupled to the BAPTA (1,2-bis(*o*-aminophenoxy)ethane-*N*,*N*,*N*′,*N*′-tetraacetic acid) chelator have been synthesized, providing a wide range of photophysical characteristics.^6–8^ Although the widely used green sensor is Oregon Green BAPTA (OGB), numerous rhodol derivatives have also been developed, including various xanthene derivatives.

As a rule of thumb, tissue penetration by the emitted light increases with wavelength.^9^ To visualize a plethora of biological processes deep inside the body, fluorescent two- and three-dimensional microscopic and mesoscopic *in vivo* imaging have gained prominence in other research areas as well, such as cancer and immune biology and stem cell studies. Nowadays, these fluorescent imaging applications are entering clinical practice, with a number of fluorescent dyes in large-scale clinical surgery trials around the world.^10^ Consequently, there is a high demand for the development and application of fluorescent dyes emitting in the far-red or near-infrared (NIR-I spectral window: 650–950 nm) regions.^11,12^ Numerous such fluorescent dyes have been synthesized and reported in the literature.^13–18^ However, the photostability of these dyes remains a frequently cited limitation that requires further improvement.^19–22^

**Fig. 1:**
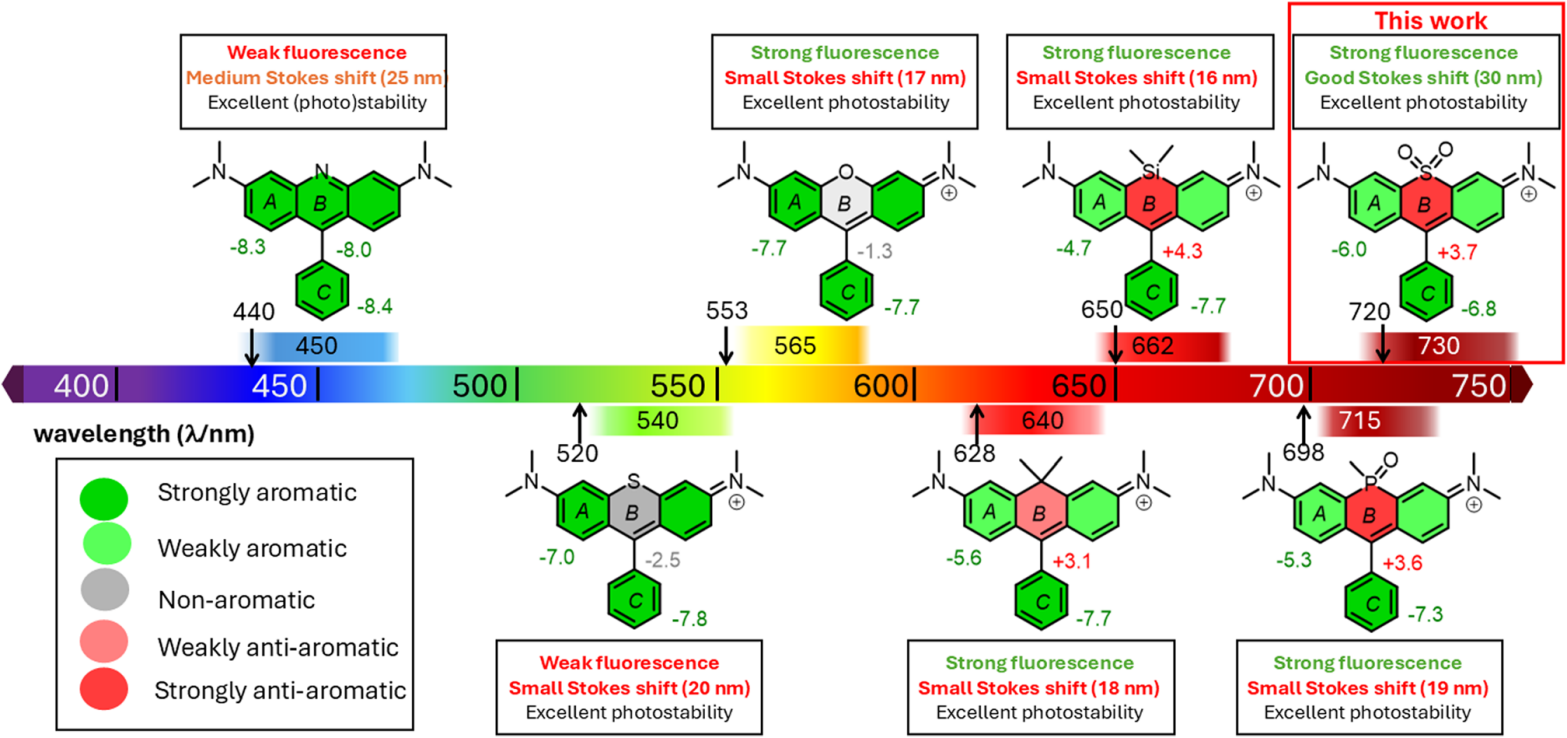
Most relevant fluorescent phenyl heteroanthracene scaffolds, illustrating their spectroscopic characteristics on the visible spectra. The arrows show the excitation wavelengths. The colour of the rings illustrates their aromaticity, measured by the NICS method. The corresponding NICS values are given in ppm and are calculated at the B3LYP/6-31G(d,p)[PCM(w)] level of theory as reported in this work.

Rhodamine fluorophores hold an outstanding position thanks to their excellent properties, such as good photostability, high molar extinction coefficients and quantum yields (QYs), resulting in high fluorescence brightness.^22–24^ By extending the conjugation of the π-aromatic framework or replacing the central oxygen atom, the initial excitation/emission maxima have been shifted from below 600 nm up to around 700 nm.^25^ Liu and co-workers recently demonstrated (summarized in Fig. 1) that dioxothioxanthene derivatives are very promising as NIR fluorescent dyes for bioimaging.^26,27^

The exchange of the central heteroatoms in the xanthene framework affects not only the spectroscopic characteristics, but also the aromaticity of the individual condensed rings A and B (Fig. 1).

The more electron-deficient the heteroatom, the more antiaromatic the central ring B becomes, as measured by nucleus-independent chemical shift (NICS) calculations.^28–30^

Despite its high brightness and photostability, the application of a dioxothioxanthene derivative dye is cumbersome and scarcely reported, mainly due to its low-yielding synthesis.^31^ This synthetic bottleneck has now been overcome, opening the way for broader exploration of these promising NIR fluorophores in practical imaging applications.

## Result and discussion

For the synthesis of rhodamine-core dyes, there are two main approaches from the retrosynthetic point of view.

**Fig. 2:**
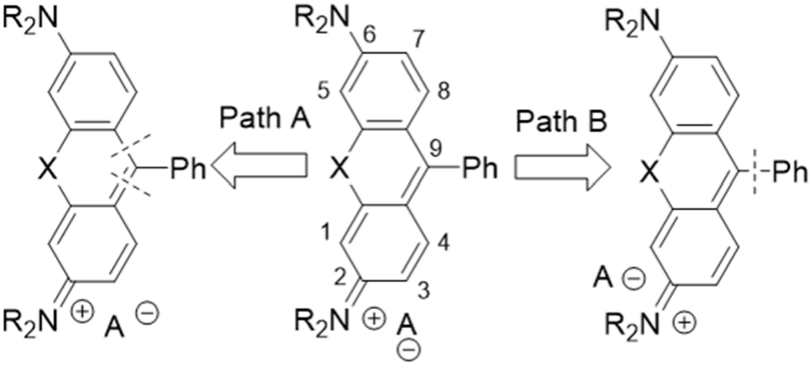
Retrosynthetic analysis of rhodamine dyes.

As shown in path “A” in Fig. 2, carbon 9 of the xanthene (anthracene) moiety originates from the coupling partner. If the coupling partner in the condensation is an acid or ester, no further oxidation is required;^32,33^ if the carbon is a carbonyl group, further oxidation is necessary.^7^

In path “B”, the xanthene part and a phenyl moiety are the two used synthons. According to this approach, the coupling is done with substituted phenyl-lithium and the appropriate oxo-compound.^34,35^ This synthesis requires using low temperatures and butyl-lithium and sometimes results in low yields. The use of Suzuki-coupling also follows path “B”. However, the Suzuki-coupling route, while enabling the synthesis of otherwise inaccessible compounds, and often requiring hardly accessible arylboroxins, is contingent upon extremely dry conditions. This strict requirement stems from the high reactivity of the triflated xanthone intermediates, which rapidly react with water or alcohols to regenerate the parent chalcogenoxanthones.^36^ The triflated xanthones are also the starting material for pyronin derivative synthesis.^37^ This derivatisation shows that position “9” of the xanthene ring system is easily attacked by nucleophiles. Both main synthetic approaches suffer from moderate or even low yields and require special reaction conditions.

Our goal was to conduct extensive research on the nucleophilic addition to the xanthene ring system and prepare a novel NIR calcium sensor for microscopic imaging, the Solomon-red-BAPTA (1).^38^ The origin of the synthesis was described as early as 1913.^39^ The oxidative coupling of xanthene derivatives has been extensively studied during the past decade using various coupling^40^ partners such as aldehydes,^41–43^ anisoles,^44^ heteroatoms,^45,46^ ketones,^47–49^ nitroalkanes.^50^ There are two possible ways to reach the active intermediate pyronin: either through the mild oxidation of the xanthene CH_2_ group or through the reduction of the 9-oxo derivative. Since the latter proceeds very sluggishly,^27^ we tried to find an appropriately mild and straightforward condition for oxidizing and coupling.

**Scheme 1:**
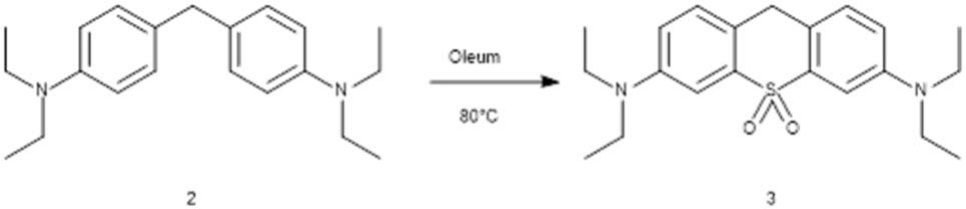
Synthesis of the sulfone-rhodamine-core.

We synthesized the sulfone-pyronin-core (3) based on an already described convenient and scalable way from 2, as shown in Scheme 1.^26,51^

**Scheme 2:**
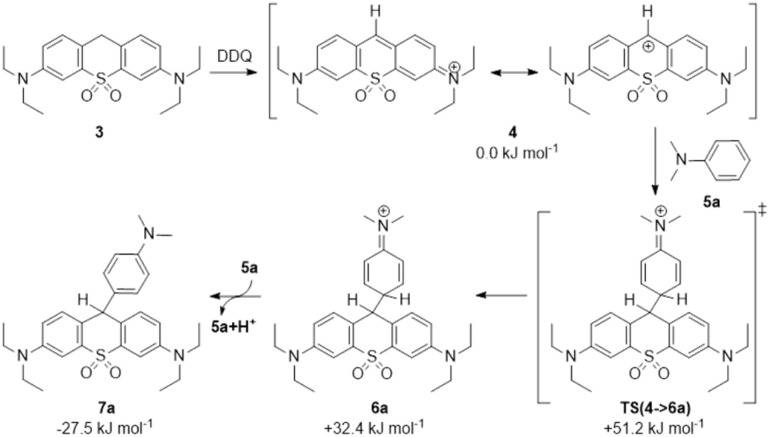
Coupling between sulfone-pyronin (3) and *N*,*N*-dimethylaniline (5a). Calculated enthalpy values are indicated in kJ mol^−1^ [B3LYP/6-31G(d,p)//PCM(tol)].

In the next step, as a model reaction, we studied the coupling between sulfone-pyronin (3) and *N*,*N*-dimethylaniline (5a), leading to compound 7a in one pot, as shown in Scheme 2.

In order to find an optimal condition, various solvents and oxidizing agents were screened and the results are summarized in Table 1.

**Table 1:** Optimization of the cross-dehydrogenative coupling (CDC) of 3 and 5a to 7a in various solvents and with different oxidising agents

Entry	Solvent	Oxidising agent	Yield^a^
1	AcOH	DDQ	67%^b^
2	NMP	DDQ	39%
3	DMF	DDQ	51%
4	DMSO	DDQ	50%
5	DCM	DDQ	52%
6	MeOH	DDQ	29%
7	EtOH	DDQ	51%
8	Toluene	DDQ	25%
9	HCOOH	DDQ	15%
10	TFA	DDQ	1%
11	ACN	DDQ	19%
12	Pyridine	DDQ	16%
13	AcOH/H_2_O 1/1	DDQ	40%
14	AcOH	(NH_4_)_2_S_2_O_8_	0%
15	AcOH	K_2_S_2_O_8_	0%
16	AcOH	H_2_O_2_	0%
17	AcOH	Na_2_CO_3_·1.5H_2_O_2_	0%
18	AcOH	Perurea	0%

a Yields of 7a are determined by HPLC using toluene as an internal standard.

b Isolated yields of 7a.

The best result was obtained using glacial acetic acid and DDQ (Entry 1). Surprisingly DDQ and TFA or formic acid (Entry 10 and 9, respectively) gave very low yields, on the other hand aqueous 50% acetic acid gave a fair yield (Entry 13). Both a rapid colour-change and HPLC measurements showed that the oxidation of sulfone-pyronin (3) is completed in less than 10 min to yield the fluorescent intermediate (4).^27^ To avoid the side reactions between the oxidizing agent and the coupling anilinic partner, 5a was added after an initial period of time (*ca.* 5–10 min). The theoretical study [B3LYP/6-31G(d,p)//PCM(tol), Fig. 4] confirms that the coupling reaction (4 → 6a) exhibits a very smooth transition state [TS(4 → 6a), 51.2 kJ mol^−1^] and is followed by an exothermic deprotonation to 7a, using 4 as a reference point.

To evaluate the substrate scope and the general feasibility of the transformation, various nucleophilic coupling partners were investigated under the optimized conditions (Table 2).

**Table 2:** Substrate scope of the test reaction

Entry	Coupling partner	Yield^a^
1	Aniline (5b)	67%
2	*N*,*N*-Dimethylaniline (5a)	39%
3	*N*,*N*-Diethylaniline (5c)	51%
4	*N*,*N*-Dipropylaniline (5d)	50%
5	*N*,*N*-Dibutylaniline (5e)	52%
6	Pyridine (5f)	—
7	Benzimidazole (5g)	—
8	Indole (5h)	80%
9	Benzidine (5i)	—
10	Anthranilic acid (5j)	50%
11	Salicylic acid (5k)	—
12	3-Hydroxy-2-naphthoic acid (5l)	—
13	3-Amino-2-naphthoic acid (5m)	—
14	Pyrogallol (5n)	75%
15	*o*-Phenylenediamine (5o)	70%
16	2-Aminophenol (5p)	30%
17	4-Aminophenol (5q)	—
18	*p*-Phenylenediamine (5r)	—
19	3-Aminophenol (5s)	43%

a isolated yields.

In contrast to most amines, benzidine (5i) failed to react under these conditions. Regarding carboxylic acid derivatives, anthranilic acid (5j) gave a fair yield, whereas salicylic acid (5k), 3-hydroxy-2-naphthoic acid (5l), and 3-amino-2-naphtholic acid (5m) resulted in no product formation. The coupling resulted in good yields in case of aromatic compounds with multiple electron-donating groups: pyrogallol (5n), *o*-phenylenediamine (5o) and 2-aminophenol (5p) Notably, while 4-aminophenol (5q) and *p*-phenylenediamine (5r) did not yield *ortho*-adducts, 3-aminophenol (5s) exhibited unique reactivity. In this case, the reaction occurred at both the 4- and 6-positions, providing a diadduct in reasonable yield, demonstrating that *ortho*-substitution is possible under specific electronic configurations.

Aniline derivatives (5a–e) afforded the corresponding products in good to excellent yields, even with relatively short reaction times. While pyridine (5f) and benzimidazole (5g) proved to be unreactive, indole (5h) underwent coupling in high yield (80%).

Consequently, two primary conclusions can be drawn: first, the presence of an electron-donating group is essential for the reaction to proceed, and second, *ortho*-adducts are generally suppressed, unless favored by the substitution pattern of the nucleophile, as seen with 5s.

**Scheme 3:**
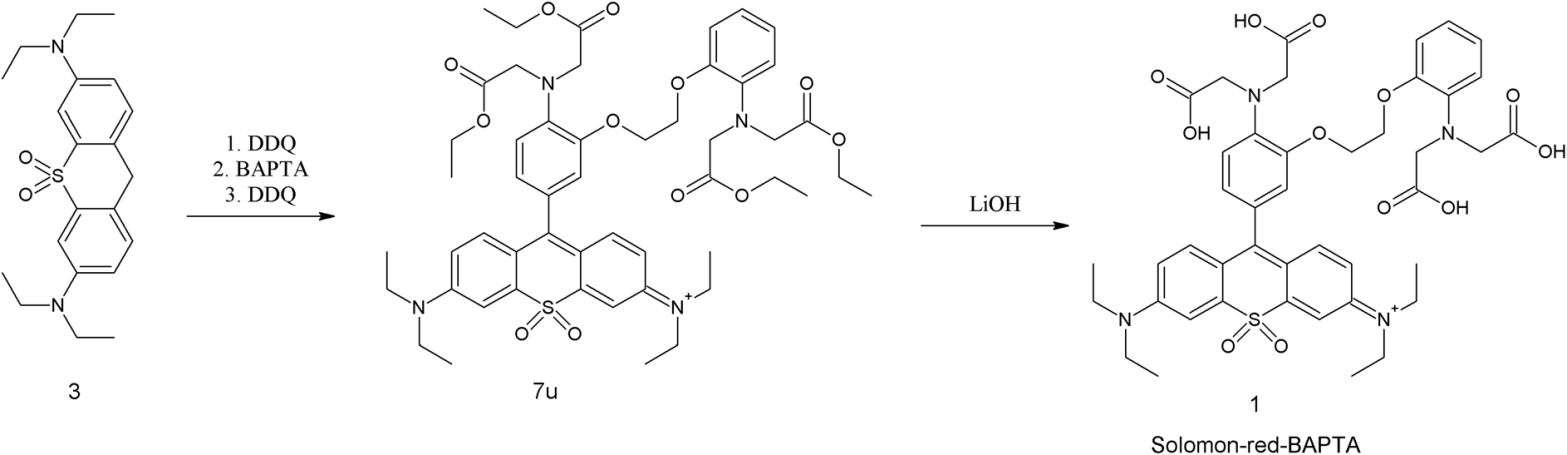
Synthesis of Solomon-red-BAPTA.

After the successful optimization and demonstration of the feasibility of DDQ-mediated cross-dehydrogenative coupling (CDC), we turned our attention to our main goal, namely, coupling with BAPTA esters (5t, 5u). Since this chelating compound is also an aniline derivative, we were expecting a successful reaction, however, an additional hydroxy substituent was required for a successful reaction as described by Minta and coworkers.^52^ The reaction time proved to be longer (2 h at RT) than that of unsubstituted anilines. The resulting non-fluorescent Et-BAPTA-sulfone-pyronin (7u) can be oxidized to fluorescent 8u either in a consecutive step after purification or in a one-pot procedure by adding an additional equivalent of DDQ to the reaction mixture as shown in Scheme 3.

Yields for the two-step and the one-pot methods are 37% and 25%, respectively. The synthesized ethyl ester was hydrolysed under conventional basic conditions (aq. LiOH) to afford the BAPTA-based sensor 1.

**Fig. 3:**
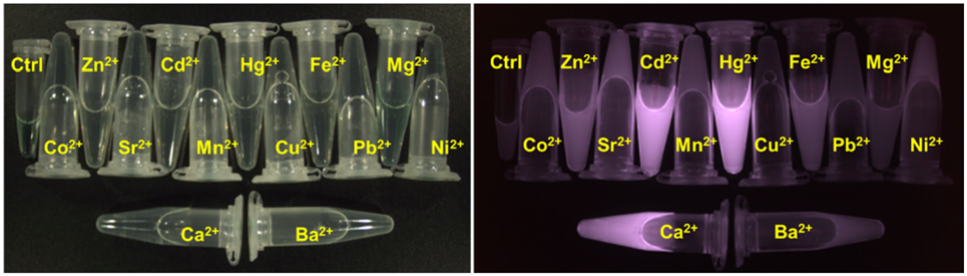
Solomon-red-BAPTA (1) under visible and NIR light: control, Co^2+^, Zn^2+^, Sr^2+^, Cd^2+^, Mn^2+^, Hg^2+^, Cu^2+^, Fe^2+^, Pb^2+^, Mg^2+^, Ni^2+^, Ca^2+^, and Ba^2+^.

Following isolation, the photophysical properties were characterized, revealing a molar extinction coefficient *t* (*ε* = 6760 M^−1^ cm^−1^, apo-state, see Fig. SI[X]) and a respectable quantum yield (:*φ* ≈ 0.25) upon Ca^2+^ complexation. While the free sensor 1 is virtually non-fluorescent in aqueous media, it displays a dramatic “turn-on” response in the presence of Ca^2+^, Cd^2+^ and Hg^2+^ (Fig. 3). Due to this high signal-to-background ratio and striking visual brightness, sensor 1 was designated as Solomon-red-BAPTA.

The selectivity of the probe was first screened against a series of biologically relevant metal ions. As shown in Fig. 3, cations such as Mg^2+^, Fe^2+^, or Sr^2+^ elicited negligible fluorescence responses, confirming the high specificity of the BAPTA scaffold within this chemical environment. These experimental findings are in excellent agreement with our computational models, which suggest that the hybrid system is optimally configured to favor the coordination of Ca^2+^ and its electronic analogues over smaller and harder cations.

**Fig. 4:**
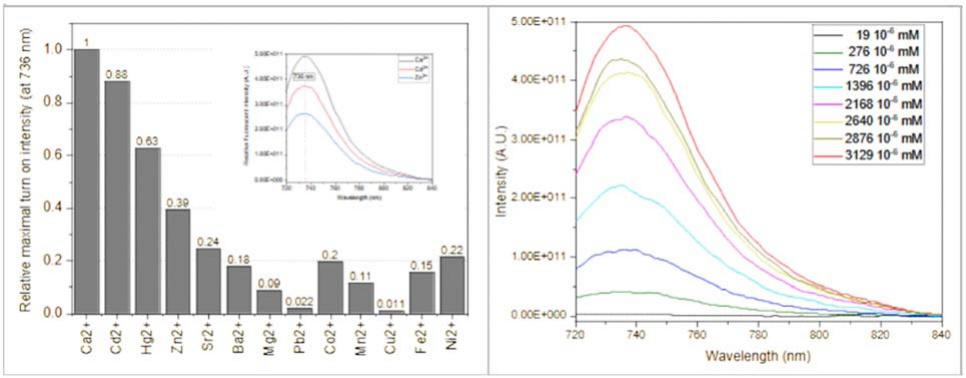
Normalized fluorescence intensity of Solomon-red-BAPTA (1) in the presence of 0.1 mM different metal ions (light blue) at 736 nm. Inset shows the spectra in the case of 0.1 mM Ca^2+^, Cd^2+^ and Zn^2+^.

**Fig. 5:**
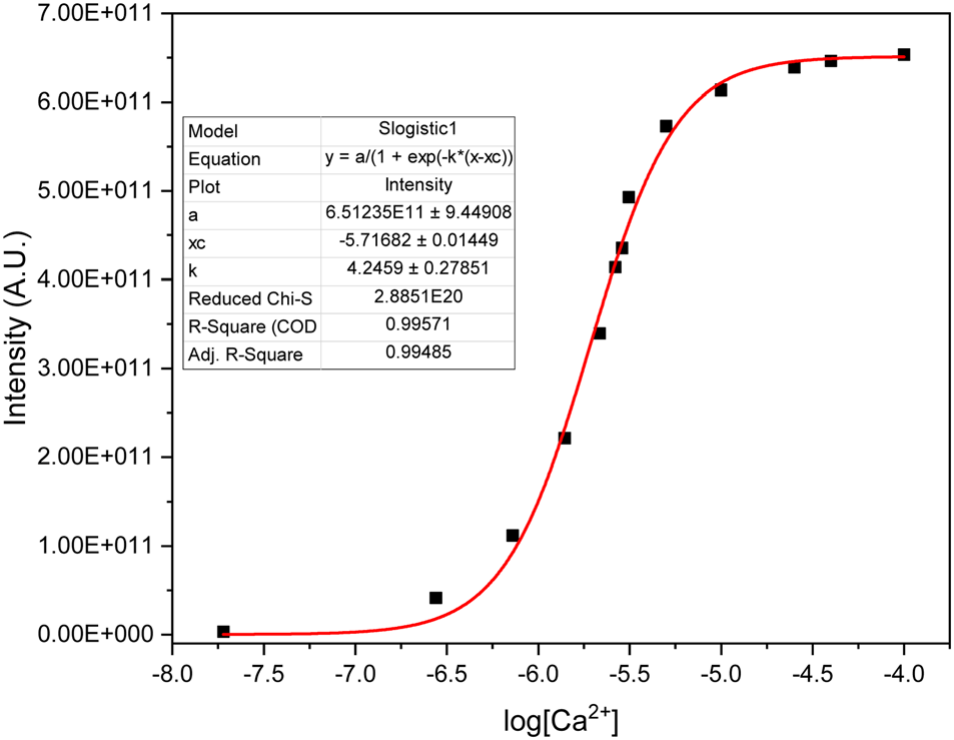
Ca^2+^ concentration dependence of the fluorescence of Solomon-red-BAPTA (1).

Additional properties of 1 were investigated to highlight and confirm its applicability. The absorption maximum was at 712 nm, while the emission maximum was at 736 nm, providing enough Stokes shift (24 nm) to significantly separate the absorption and emission spectra and minimize light scattering at the detection side. The growth of the emission maxima was as high as 160, 140 and 62 folds in the presence of Ca^2+^, Cd^2+^ and Zn^2+^, respectively. The fluorescent spectra did not change under various Ca^2+^ concentrations, as shown in Fig. 4, right. The calculated apparent binding constant 
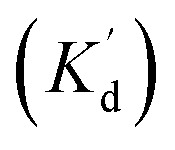
 for Ca^2+^ is 
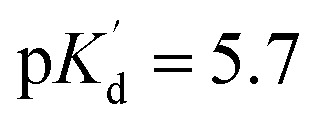
 (*ca.* 2 µM) as determined by a competitive fluorescent titration (Fig. 5). The dynamic range of sensor 1 is 0.3–10 µM, which is similar to other reported sensors. We have determined a relatively high water solubility of >10 mg l^−1^ for sensor 1, the estimated concentration exceeds the probe concentration needed in biology in general.

**Fig. 6:**
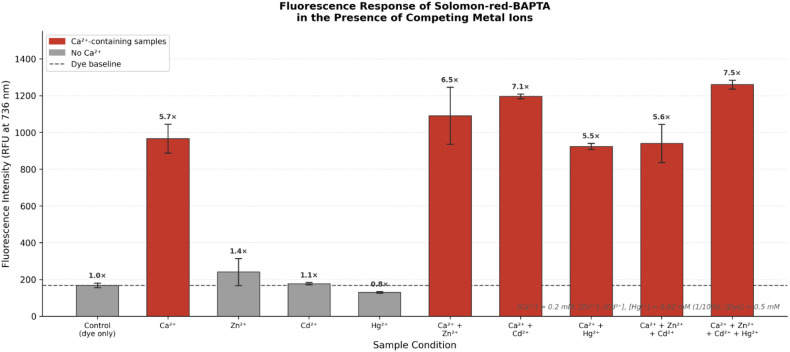
Fluorescence response of Solomon-red-BAPTA in the presence of competing metal ions.

To further evaluate the analytical robustness of Solomon-red-BAPTA, competitive binding assays were performed (Fig. 6). At the investigated concentrations, the presence of Zn^2+^, Cd^2+^ or Hg^2+^ alone resulted in well-defined, discrete fluorescence responses (0.8–1.4 fold relative to the baseline). In the specific case of Hg^2+^, a slight attenuation of the background signal was observed. This phenomenon is likely attributable to a subtle heavy-atom effect, which is readily overcome by the strong chelation-enhanced fluorescence (CHEF) in the presence of Ca^2+^. In multi-ion systems containing Ca^2+^, a sustained and significant enhancement (up to 7.5-fold) was observed regardless of the presence of other divalent cations, demonstrating that the probe remains highly responsive even in complex ionic environments.

**Fig. 7:**
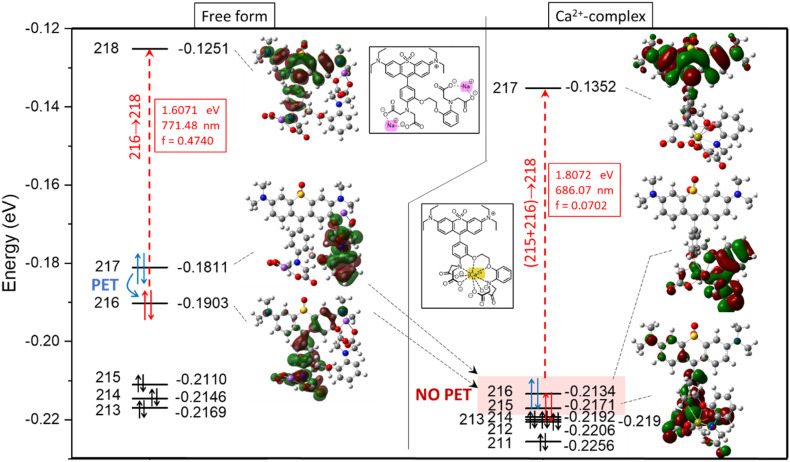
Computed molecular orbitals (MOs) at the B3LYP/6-31G(d,p)[PCM(water)] level of theory for the free form (left) and the Ca^2+^ complex (right) of Solomon-red-BAPTA, along with their prevalent electronic excitations (red boxes) calculated at the TD-B3LYP/6-31G(d,p)[PCM(water)] level. The photoinduced electron transfer (PET) may be a favourable quenching process for the free form, while PET is excluded in the case of complex form due to the mixed S1 excitation.

The computed photophysical properties, including absorption and emission wavelengths and oscillator strengths, of the free and complexed forms of 1 were calculated at the TD-B3LYP/6-31G(d,p)[PCM(water)]^53,54^ level of theory using Gaussian 16.^55^ The experimentally observed fluorescence quenching of the free sensor may be attributed to the photoinduced electron transfer (PET) mechanism. Here, the predicted electronic excitation of the most stable conformers of the uncomplexed form of 1 (with 2 Na^+^ ions) can be assigned to the HOMO − 1 (216) to LUMO (218) orbitals, as shown in Fig. 7, left. The two Na^+^ ions are included to ensure the overall charge neutrality during the complexation process with Ca^2+^. In this structure, the HOMO (217) is localized mainly on the electron-rich aromatic moiety of the BAPTA unit, spatially separated from the fluorophore, whereas the HOMO − 1 (216) corresponds predominantly to the chromophoric π-system. Following excitation, electron transfer from the energetically accessible BAPTA-centered HOMO (217) to the partially depleted chromophore-centered orbital can occur efficiently, providing a non-radiative relaxation pathway that competes with fluorescence emission. Here, the electron from HOMO (217) can occupy the vacant position of HOMO − 1 (216), preventing the radiative deexcitation of the electron from LUMO (218) and forcing non-radiative relaxations. This PET process therefore results in strong fluorescence quenching of the free sensor, which is similar to that of related BAPTA-based fluorescent probes.^56–58^

Upon complexation with Ca^2+^, substantial changes in the frontier molecular orbital energies and distributions were observed. The coordination of Ca^2+^ significantly stabilizes the electron-donating orbitals associated with the BAPTA unit, lowering their energies and thereby suppressing the thermodynamic driving force of the PET process. In the Ca^2+^-bound form of 1, the photochemical process represents a more complex situation, where the electronic excitation occurs from the mixed HOMO (216)/HOMO − 1 (215) to LUMO (217) (see Fig. 7, right). As a consequence, the PET-mediated non-radiative deactivation pathway becomes energetically unfavorable, allowing radiative decay to dominate and thereby restoring strong fluorescence emission. These computational findings are in good agreement with the experimentally observed fluorescence turn-on response upon Ca^2+^ binding.

## Conclusions

In summary, we have successfully developed a novel class of non-fluorescent sulfo-pyronin adducts, demonstrating that aniline substitution serves as a versatile and synthetically unique platform for the design of “turn-on” fluorogenic probes. The primary novelty of this work lies in the utilization of the sulfo-pyronin scaffold, which enables a robust fluorescence response upon the oxidative recovery of the aromatic system, characterized by a distinct 24 nm Stokes shift. The performance of the resulting Solomon-red-BAPTA was rigorously characterized by determining its molar extinction coefficient (*ε*) and fluorescence quantum yield (*φ* ≈ 0.25). Compared to existing red/NIR Ca^2+^ probes, our system offers a straightforward synthetic route and a remarkably high signal-to-background ratio. Furthermore, we evaluated the selectivity and analytical robustness of the probe by investigating the Ca^2+^ response in the presence of potentially interfering ions such as Zn^2+^, Cd^2+^, and Hg^2+^. These competitive assays confirmed that the sensor maintains its efficacy for Ca^2+^ detection even in complex ionic environments. This selectivity profile was further elucidated by computational studies, which are in excellent agreement with experimental observations. The theoretical results suggest that the coordination environment of the BAPTA-sulfo-pyronin hybrid is optimally configured for the ionic radii of these specific cations. These findings establish Solomon-red-BAPTA as a valuable tool for selective cation sensing and provide a theoretical framework for the future development of pyronin-based fluorogenic sensors with tunable selectivity.

**Fig. 8:**
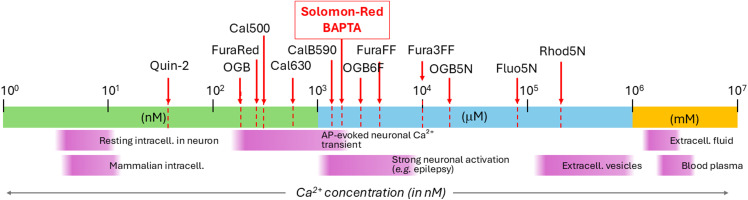
Schematic compares typical free Ca^2+^ levels from resting intracellular nanomolar concentrations to extracellular and vesiclar millimolar pools. The apparent 
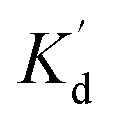
 of Solomon-red-BAPTA, approximately 1.7–2.0 µM, places it in the medium-affinity range suitable for pronounced intracellular Ca^2+^ transients.

Fig. 8 represents the biologically relevant free Ca^2+^ concentration ranges, spanning from resting cytosolic nM levels to extracellular and vesicular mM Ca^2+^ pools, together with several well-known fluorescent sensor molecules. The experimentally determined Ca^2+^
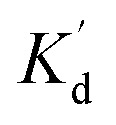
 of Solomon-red-BAPTA is indicated at approximately 1.7–2.0 µM, placing it in the biologically very relevant medium-affinity region and showing that it is better suited for detecting pronounced, *e.g.*, epileptic-type intracellular Ca^2+^ elevations rather than small near-resting Ca^2+^ fluctuations.

## Author contributions

Conceptualization: Z. K.; methodology: G. R., L. M., D. B.; software: F. H., K. S., D. M.; validation: Z. K., Z. M., I. M.; formal analysis: F. H., D. M., K. S., B. D; investigation: G. R., L. F.; L. M., I. M., Z. K.; resources: L. F., D. M.; data curation: L. F., Z. M., L. M.; writing—original draft preparation: Z. K., G. R.; writing—review and editing: Z. K., Z. M., I. M., L. F.; visualization: L. F., Z. M.; supervision: Z. K., I. M.; project administration: Z. K.; and funding acquisition: Z. K., I. M., D. M. All authors have read and agreed to the published version of the manuscript.

## Conflicts of interest

There are no conflicts of interest to declare.

## Supplementary Material

RA-016-D6RA01589A-s001

RA-016-D6RA01589A-s002

## Data Availability

All experimental data, synthetic procedures, analytical measurements, and characterization results supporting the findings of this study are available in the supplementary information (SI) files. These include detailed synthesis methods, NMR spectra, HRMS data, HPLC analyses, photophysical measurements, and computational details. The SI corresponding to this work has been uploaded as part of the submission package and can be accessed through the Royal Society of Chemistry (RSC) publication platform upon acceptance of the manuscript. Supplementary information: detailed experimental descriptions, structural formulas, sample preparation details, HR-MS measurements, NMR data with signal assignments, and supplementary spectroscopic characterization data.The accompanying XLSX file contains the raw absorption and emission spectroscopic measurement data. See DOI: https://doi.org/10.1039/d6ra01589a.
