# Supplementary material for: Straightforward synthesis of Solomon-red-BAPTA as NIR fluorescent sensors via metal-free oxidative coupling
Source: RSC Adv. 2026 Jun 1;16(32):29450–7. doi: 10.1039/d6ra01589a (PMC13227500; doi:10.1039/d6ra01589a)
Supplement: RA-016-D6RA01589A-s001 [file RA-016-D6RA01589A-s001.pdf]

Supplementary Information (SI) for RSC Advances.
This journal is © The Royal Society of Chemistry 2026

Straight-forward Synthesis of Solomon-red-BAPTA as NIR Fluorescent sensors via Metal-free Oxidative Coupling

Gergő Riszter[1,2,3], László Forgách[4,5], Zoltán Mucsi[6,7], Laura Molnár[2], Dóra Bogdán[1], Fatemeh Heydari[4,5], Domokos Máthé[4,5,8], István Mándity[1,3,9], Gömöry Ágnes[10], Zoltán Kaleta[1,3,9*]

[1] Department of Organic Chemistry, Semmelweis University, Hőgyes Endre Street 7, H-1092 Budapest, Hungary

[2] PROGRESSIO Engineering Bureau Ltd., Muhar u. 54, H-1028 Budapest, Hungary

[3] Artificial Transporters Research Group, Institute of Materials and Environmental Chemistry, Research Centre for Natural Sciences, Eötvös Loránd Research Network, Magyar Tudósok Körútja 2, H-1117, Budapest, Hungary

[4] Department of Biophysics, Semmelweis University, 37-47 Tűzoltó utca, Budapest, 1094, Hungary

[5] In Vivo Imaging Advanced Core Facility, Hungarian Center of Excellence for Molecular Medicine (HCEMM), 37-47 Tűzoltó utca, Budapest, 1094, Hungary

[6] BrainVisionCenter, 1094 43-45. Liliom str., Budapest, Hungary

[7] Institute of Chemistry, University of Miskolc 3515 Miskolc-Egyetemváros, A/2 building, Hungary

[8] CROmed Translational Research Centers, 37-47 Tűzoltó Street, Budapest, 1094, Hungary

[9] Centre for Pharmacology and Drug Research & Development, Semmelweis University, Budapest, Hungary

[10] MS Proteomics Research Group, Institute of Organic Chemistry, Research Centre for Natural Sciences, HUN-REN Hungarian Research Network, Magyar Tudósok Körútja 2, H-1117, Budapest, Hungary

*Corresponding Author: Zoltán Kaleta: Email Address: kaleta.zoltan@semmelweis.hu

All reactions were carried out under an argon atmosphere.

Aniline, iodoethane, 1-bromopropane, 1-bromobutane were purchased from TCI and used as received.

2-nitrophenol was purchased from Alfa Aesar and used as received.

1-bromo-2-chloroethane, ethyl bromoacetate, lithium hydroxide monohydrate, tetramethyl 2,2',2'',2'''-(((ethane-1,2-diylbis(oxy))bis(2,1-phenylene))bis(azanetriyl)) tetraacetate, *N*,*N*-dimethylaniline were purchased from Sigma Aldrich and used as received.

Diisopropylethylamine was purchased from VWR and used as received.

2,3-dichloro-5,6-dicyano-p-benzoquinone (DDQ) was purchased from Fluorochem and used as received.

N,*N*-Dimethylformamide, potassium carbonate, ethyl acetate, sodium sulfate, glacial acetic acid, 30% formaldehyde solution, fuming sulfuric acid, sodium iodide, acetonitrile, iron, ethanol, tetrahydrofuran were purchased from Molar and used as received.

N,*N*-dialkyl anilines were synthesized based on literature procedure. [i]

NMR spectra were recorded at room temperature on Varian Mercury Plus spectrometer (^{1}H: 400 MHz and ^{13}C 100 MHz) or on Bruker Avance III spectrometer equipped with a cryoprobe (^{1}H: 500 MHz and ^{13}C 125 MHz). Amounts of 5–10 mg of compounds were dissolved in 0.6 ml DMSO-*d6*, $CDCl_3$ or CD_3OD and transferred to 5 mm NMR sample tubes. Chemical shifts are given on the δ-scale and referenced to the solvent (DMSO-*d6*: δ_C=39.50 ppm and δ_H=2.50 ppm; $CDCl_3$: δ_C=77.00 ppm and δ_H=7.26 ppm; CD_3OD: δ_C=49.00 ppm and δ_H=3.31 ppm or CD_3CN: δ_H=1.95 ppm). For structure elucidation, one-dimensional ^{1}H, ^{13}C, two-dimensional ^{1}H-^{13}C-HSQC, ^{1}H-^{13}C-HMBC measurements were run. For ^{1}H NMR measurement 24 K data points, 2.0 s acquisition time and 6400 Hz sweep width were used. ^{13}C spectra were recorded with 62 K data points and 24000 Hz sweep width. For 2D measurements, in case of the gHSQC spectrum the sweep width in F2 was 3000 Hz; data points (t2 × t1) were acquired with 1 K × 128, in case of the gHMBC spectrum the sweep width in F2 was 3000 Hz; data points (t2 × t1) were acquired with 1 K × 256, respectively.

HRMS measurements were carried out on a Waters QTOF Premier (Manchester, UK) instrument operated in positive ESI mode.

The solvent dependence was determined by Agilent 1100 HPLC.

The spectral properties were determined by NanoDrop 1100 Spectrophotometer, NanoDrop 3300 Fluorospectrometer and Edinburgh FLS 980 fluorescence spectrophotometer.

***N,N*-diethylaniline:**

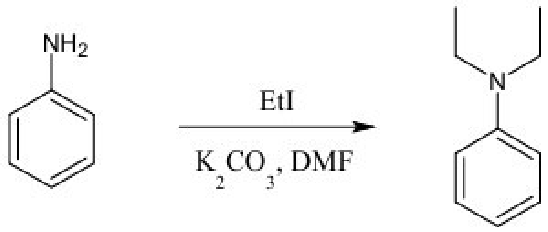

Aniline (160 mmol; 14,9 g) was dissolved in dimethylformamide (DMF) in a round bottom flask, a magnetic stirrer and potassium carbonate (480 mmol; 66,3 g) was added. Iodoethane (480 mmol; 74,9 g) was added dropwise to the resulted suspension under vigorous stirring at room temperature. The resulted mixture was stirred at room temperature overnight. After the completion, what was determined by TLC or HPLC-MS, water and ethyl acetate were added. The two phases were transferred into a separatory funnel. The aqueous layer was extracted 3 times with ethyl acetate. The combined organic layers were washed with brine, dried over sodium sulfate, and the solvents were evaporated. The residue used without further purification. Yield: 21g (141 mmol, 88%). NMR was identical with reported data's.[i]

(2): 4,4'-methylene-bis(*N,N*-diethylaniline):

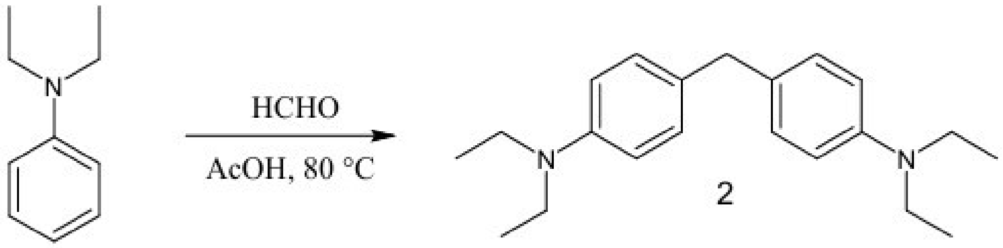

N,N-diethyl aniline (160 mmol, 23,9 g) was dissolved in glacial acetic acid in a round bottom flask. A magnetic stirring bar and 30% formaldehyde-water solution (320 mmol; 32 g) were added to the flask, and the resulted mixture was stirred at 80°C for 1h. The completion of the reaction was determined by HPLC-MS and the mixture was cooled to room temperature the solvents were evaporated. The residue was carefully neutralized with sodium hydrogen carbonate solution. The aqueous solution was transferred to a separatory funnel and was extracted 3 times with ethyl acetate. The combined organic layers were washed with brine, dried over sodium sulfate, and the solvents were evaporated. Yield is around 24,5 g (79 mmol, 99%). The residue was used without further purification. NMR was identical with reported data's[ii].

<u>(3):</u> 3,6-bis(diethylamino)-)9H-thioxanthene-10,10-dioxide:

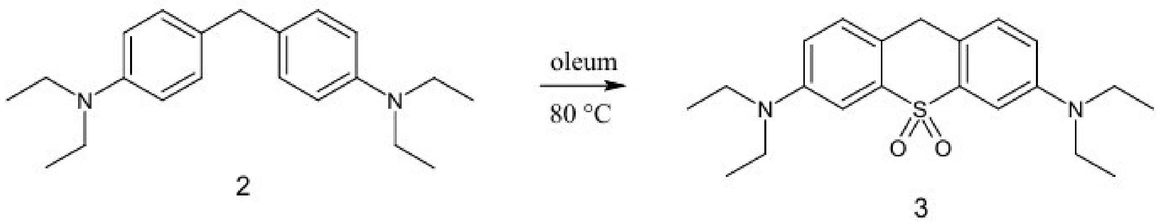

2

3

A round bottom flask was equipped with a magnetic stirring bar, charged with fuming sulfuric acid (20%, 1ml/mmol of **2**). 4,4'-methylenebis(*N,N*-diethylaniline) (100 mmol; 31 g) was added carefully and the resulted mixture was stirred at 80°C. The reaction was followed by HPLC, and was ready in one hour. The reaction mixture was poured onto ice, 10 times of its volume, and carefully neutralized with aqueous ammonia, transferred into a separatory funnel, and was extracted 3 times with ethyl acetate. The combined organic layers were washed with brine, dried over sodium sulfate, and solvents were evaporated. The crude product was purified by crystallization from small amount of ethyl acetate to yield 22,7 g (61%) light brown crystals.

<u>NMR:</u>

1 H NMR (400 MHz, CD 3 CN) δ 7.28 (d, J = 8.6 Hz, 2H), 7.19 (d, J = 2.8 Hz, 2H), 6.84 (dd, J = 8.6, 2.8 Hz, 2H), 3.94 (s, 2H), 3.40 (q, J = 7.1 Hz, 8H), 1.13 (t, J = 7.0 Hz, 12H).

13 C NMR (101 MHz, CD 3 CN) δ 147.9, 139.4, 130.9, 125.9, 118.3, 116.2, 105.9, 45.1, 32.1, 12.6.

<u>1,2-bis(2-nitrophenoxy)ethane:</u>

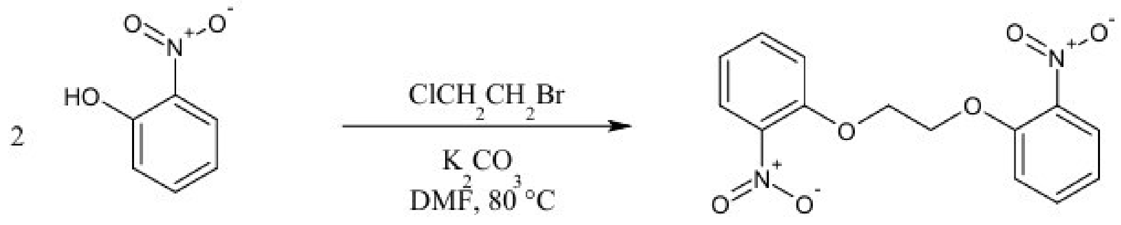

A round botton flask was equipped with a magnetic stirring bar, charged with 2-nitrophenol (14,4 mmol; 2 g), potassium carbonate (26,1 mmol; 3,61 g), sodium iodide (0,65 mmol; 98 mg) and acetonitrile. 1-bromo-2-chloroethane was added to the stirred suspension, and the mixture was heated to 70°C overnight. After the completion was determined by TLC or HPLC-MS water was added. The two phases were transferred into a separatory funnel. The aqueous layer was extracted 3 times with ethyl acetate. The combined organic layers were washed with brine, dried over sodium sulfate, and the solvents were evaporated. Yield: 78%, NMR was identical with reported data's[iii].

2,2'-[ethane-1,2-diylbis(oxy)]dianiline:

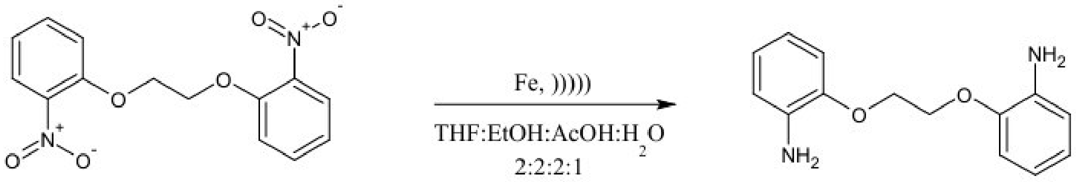

A suspension of 1,2-bis(2-nitrophenoxy)ethane (7,5 mmol, 2,28 g) and iron (75 mmol, 4,19 g) in solvent mixture of tetrahydrofuran, ethanol, glacial acetic acid and water (2:2:2:1, sum 63 ml) was sonicated for 1h. After completion of the reaction, sat. sodium carbonate solution was added, the layers were separated, the aqueous layer was extracted 3 times with ethyl acetate. The combined organic layers were dried over sodium sulfate and evaporated to yield crude product. Yield 91%, NMR was identical with reported data's[iv].

(5t) Et-BAPTA: Tetraethyl 2,2',2'',2'''-(((ethane-1,2-diylbis(oxy))bis(2,1-phenylene)) bis(azanetriyl)) tetraacetate:

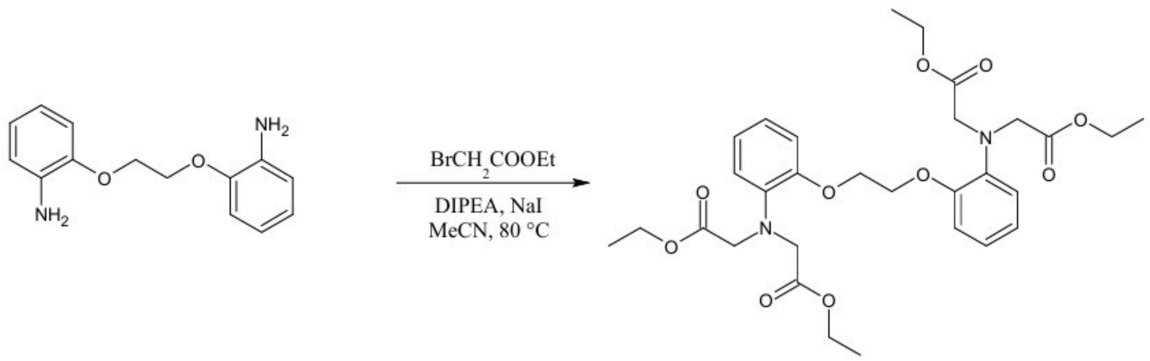

A round bottom flask was equipped with a magnetic stiring bar, charged with 2,2'-[ethane-1,2-diylbis(oxy)]dianiline (13,1 mmol; 3,2 g), diisopropylethylamine (DIPEA) (131 mmol; 1,46 ml), sodium iodide (13,1 mmol; 1,96 g), ethyl bromoacetate (52,4 mmol; 5,81 ml) and acetonitrile. The mixture was heated to 80°C over the night. After the completion was determined by TLC or HPLC-MS the solvent was evaporated. Water and ethyl acetate were added, the layers were separated, the aqueous layer was extracted 3 times with ethyl acetate. The combined organic layers were dried over sodium sulfate and evaporated. Yield 90%, NMR was identical with reported data's[v].

^{1}H NMR (400 MHz, CD_3OD) δ: 6.96-6.87 (m, 4H), 6.85 (td, J=7.8, 1.5 Hz, 2H), 6.78 (dd, J=7.8, 1.5 Hz, 2H), 4.23 (s, 4H), 4.12 (s, 8H), 3.99 (q, J=7.2 Hz, 8H), 1.10 (t, J=7.2 Hz, 12H)

^{13}C NMR (100 MHz, CD_3OD) δ: 173.5, 151.7, 140.6, 123.0, 122.2, 119.6, 114.0, 68.4, 61.9, 54.6, 14.3

<u>7b:</u>

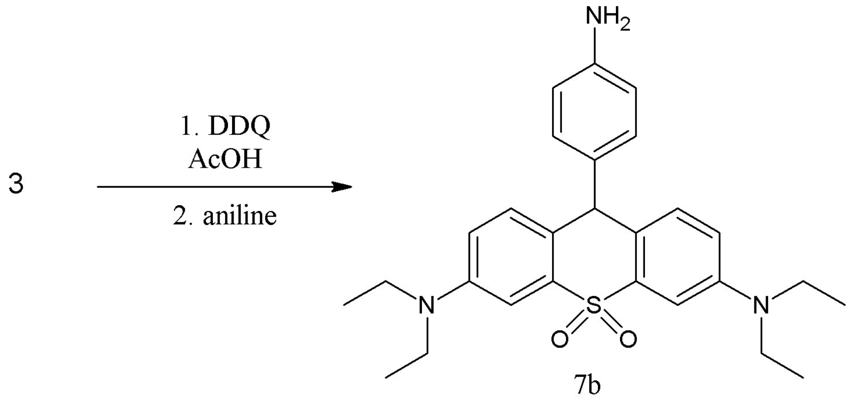

7b

To a solution of **3** (1 mmol; 373 mg) in glacial acetic acid 2,3-dichloro-5,6-dicyano-p-benzoquinone (DDQ) (1 mmol; 227 mg) was added and the reaction mixture was stirred at rt for 5 minutes, followed by HPLC-MS. After that, aniline (1 mmol; 91,2 µl) was added and the solution was stirred at rt for 1h. After the completion was determined by HPLC-MS, the solvent was evaporated. To the residue ethyl acetate and sat. sodium hydrogen carbonate solution was added (to remove the forming 4,5-dichloro-3,6-dihydroxybenzene-1,2-dicarbonitrile). The organic layers were washed with brine, dried over sodium sulfate, and the solvent was evaporated. The product was crystallized from small volume of ethyl acetate. Yield 57%.

<u>HRMS:</u> m/z calculated: 464.2372, measured: 464.2367: $[M+H]^+$, 232.6203: $[M+H]^{++}$

<u>NMR:</u>

^{1}H NMR (600 MHz, $CDCl_3$) δ 7.33 (d, *J*=2.6 Hz, 2H), 7.07 (d, *J*=8.2 Hz, 2H), 6.97 (d, *J*=8.8 Hz, 2H), 6.69-6.64 (m, *J*=8.8, 4H), 5.17 (s, 1H), 3.38 (q, *J*=7.1 Hz, 8H), 1.15 (t, *J*=7.1 Hz, 12H)

^{13}C NMR (150 MHz, $CDCl_3$) δ 146.7, 145.3, 137.6, 131.5, 130.6, 130.2, 128.0, 115.4, 115.1, 105.2, 46.1, 44.4, 12.4

<u>General procedures of 7a-7r:</u>

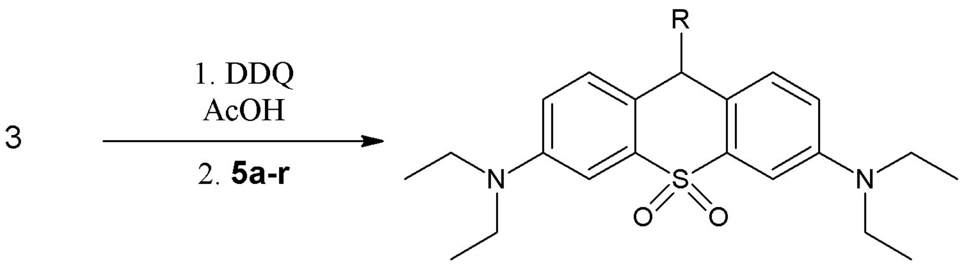

7a-r

To a solution of **3** (0.1 mmol) in glacial acetic acid DDQ (0.1 mmol) was added and the reaction mixture was stirred at rt for 5 minutes, followed by HPLC-MS. After that **5a-r** (0,15 mmol) was added and the solution was stirred at rt for 1h. After the completion was determined by HPLC-MS, the solvent was evaporated. To the residue ethyl acetate and sat. sodium hydrogen carbonate solution was added (to remove the forming 4,5-dichloro-3,6-dihydroxybenzene-1,2-dicarbonitrile). The organic layers were

washed with brine, dried over sodium sulfate, and the solvent was evaporated. The product was crystallized from small volume of methanol, or purified by RP Flash Chromatography.

7a:

Yield: 67% (crystallized)

HRMS: m/z calculated: 492.2685, measured: 492.2682: $[M+H]^+$, m/z 246.6360: $[M+H]^{++}$

NMR:

^{1}H NMR (600 MHz, $CDCl_3$) δ 7.34 (d, *J*=2.3 Hz, 2H), 7.16 (d, *J*=8.3 Hz, 2H), 6.97 (d, *J*=8.8 Hz, 2H), 6.73 (d, *J*=8.3 Hz, 2H), 6.66 (dd, *J*=8.8, 2.3 Hz, 2H), 5.20 (s, 1H), 3.38 (q, *J*=7.1 Hz, 8H), 2.94 (s, 6H), 1.15 (t, *J*=7.1 Hz, 12H)

^{13}C NMR (150 MHz, $CDCl_3$) δ 149.6, 146.6, 137.6, 130.4, 130.2, 129.2, 128.3, 115.1, 112.8, 105.2, 46.0, 44.4, 40.6, 12.4

7c:

Yield: 34% (crystallized)

HRMS: m/z calculated: 520.2998, measured: 520.2989: $[M+H]^+$, m/z 260.6513: $[M+H]^{++}$

NMR:

^{1}H NMR (600 MHz, $CDCl_3$) δ 7.34 (d, *J*=1.9 Hz, 2H), 7.11 (d, *J*=8.0 Hz, 2H), 7.01 (d, *J*=8.7 Hz, 2H), 6.67 (dd, *J*=8.7, 1.9 Hz, 2H), 6.66 (d, *J*=8.0 Hz, 2H), 5.18 (s, 1H), 3.37 (q, *J*=7.0 Hz, 8H), 3.34 (t, *J*=7.2 Hz, 4H), 1.16 (t, *J*=7.2 Hz, 6H), 1.15 (t, *J*=7.0 Hz, 12H)

^{13}C NMR (150 MHz, $CDCl_3$) δ 146.8, 146.6, 137.6, 130.6, 130.3, 128.5, 127.9, 115.1, 111.9, 105.2, 46.0, 44.4, 44.3, 12.6, 12.4

7d:

Yield: 66% (crystallized)

HRMS: m/z calculated: 548.3311, measured: 548.3326: $[M+H]^+$, m/z 274.6680: $[M+H]^{++}$

NMR:

^{1}H NMR (600 MHz, $CDCl_3$) δ 7.33 (d, *J*=1.9 Hz, 2H), 7.10 (d, *J*=8.3 Hz, 2H), 7.01 (d, *J*=8.7 Hz, 2H), 6.67 (dd, *J*=8.7, 1.9 Hz, 2H), 6.61 (d, *J*=8.3 Hz, 2H), 5.17 (s, 1H), 3.38 (q, *J*=7.1 Hz, 8H), 3.21 (t, *J*=7.6 Hz, 4H), 1.61 (sx, *J*=7.6 Hz, 4H), 1.15 (t, *J*=7.1 Hz, 12H), 0.92 (t, *J*=7.6 Hz, 6H)

^{13}C NMR (150 MHz, $CDCl_3$) δ 147.2, 146.6, 137.6, 130.5, 130.3, 128.5, 127.6, 115.1, 111.7, 105.2, 52.9, 46.1, 44.4, 20.5, 12.4, 11.4

7e:

Yield: 39% (crystallized)

HRMS: m/z calculated: 576.3624, measured: 576.3624: $[M+H]^+$, m/z 288.6824: $[M+H]^{++}$

NMR:

^{1}H NMR (600 MHz, $CDCl_3$) δ 7.34 (d, *J*=1.6 Hz, 2H), 7.10 (d, *J*=8.3 Hz, 2H), 7.02 (d, *J*=8.6 Hz, 2H), 6.67 (dd, *J*=8.6, 1.6 Hz, 2H), 6.61 (d, *J*=8.3 Hz, 2H), 5.17 (s, 1H), 3.38 (q, *J*=6.9 Hz, 8H), 3.24 (t, *J*=7.4 Hz, 4H), 1.57 (qui, *J*=7.4 Hz, 4H), 1.34 (sx, *J*=7.4 Hz, 4H), 1.15 (t, *J*=6.9 Hz, 12H), 0.95 (t, *J*=7.4 Hz, 6H)

^{13}C NMR (150 MHz, $CDCl_3$) δ 147.2, 146.6, 137.6, 130.4, 130.3, 128.4, 127.6, 115.1, 111.7, 105.2, 50.8, 46.1, 44.4, 29.4, 20.4, 14.0, 12.4

7h:

Yield: 80% (purified by RP Flash Chromatography (1% TFA H2O - MeOH gradient))

HRMS: m/z calculated: 488.2372, measured: 488.2378: $[M+H]^+$

NMR:

^{1}H (DMSO-d6, 400 MHz) δ 11.09 (d, *J*=2.3 Hz, 1H), 7.42 (d, *J*=8.1 Hz, 1H), 7.36 (d, *J*=2.3 Hz, 1H), 7.14 (d, *J*=2.6 Hz, 2H), 7.11-7.03 (m, 2H), 6.96 (d, *J*=8.8 Hz, 2H), 6.85 (t, *J*=8.1 Hz, 1H), 6.75 (dd, *J*=8.8, 2.6 Hz, 2H), 5.58 (s, 1H), 3.35 (m, 8H), 1.07 (t, *J*=7.0 Hz, 12H).

^{13}C (DMSO-d6, 100 MHz) δ 146.1, 137.3, 136.7, 130.0, 127.2, 125.7, 125.7, 121.0, 119.5, 118.6, 115.2, 113.3, 111.8, 103.7, 43.7, 37.4, 12.1.

7j:

Yield: 50% (purified by RP Flash Chromatography (1% TFA H2O - MeOH gradient))

HRMS: m/z calculated: 507.6643, measured: 507.6645: $[M+H]^+$

NMR:

^{1}H (CD_3CN, 400 MHz) δ 7.76 (s, 1H), 7.17 (d, *J*=2.0 Hz, 2H), 7.15-7.07 (m, 3H), 6.80 (dd, *J*=8.0, 2.0 Hz, 2H), 6.62 (d, *J*=8.2 Hz, 1H), 5.16 (s, 1H), 3.38 (q, *J*=6.9 Hz, 8H), 1.11 (t, J=6.9 Hz, 12H).

^{13}C (CD_3CN, 100 MHz) δ 171.5, 151.5, 148.2, 138.9, 135.3, 132.7, 132.4, 131.6, 127.9, 118.0, 117.0, 106.1, 105.0, 46.8, 45.2, 12.7.

7n:

Yield: 75% (purified by RP Flash Chromatography (1% TFA H2O - MeOH gradient))

HRMS: m/z calculated: 497.2110, measured: 497.2116: $[M+H]^+$

NMR:

^{1}H (DMSO-d6, 400 MHz) δ 9.09 (brs, 1H), 8.54 (brs, 1H), 8.41 (brs, 1H), 7.10-7.00 (m, 4H), 6.85 (dd, *J*=8.8, 2.5Hz, 2H), 6.32 (d, *J*=8.4 Hz, 1H), 6.23 (d, *J*=8.4 Hz, 1H), 5.56 (s, 1H), 3.36 (m, 8H), 1.08 (t, *J*=7.0 Hz, 12H).

^{13}C (DMSO-d6, 100 MHz) δ 146.0, 144.8, 144.4, 137.0, 133.0, 130.3, 127.4, 120.0, 119.6, 115.7, 106.9, 103.0, 43.8, 38.8, 12.2.

7o:

Yield: 70% (purified by RP Flash Chromatography (1% TFA H2O - MeOH gradient))

HRMS: m/z calculated: 479.2481, measured: 479.2483: $[M+H]^+$

NMR:

^{1}H (DMSO-d6, 400 MHz) δ 7.09 (d, *J*=8.8 Hz, 2H), 7.05 (d, *J*=2.4 Hz, 2H), 6.86 (dd, *J*=8.8, 2.4 Hz, 2H), 6.43 (d, *J*=7.8 Hz, 1H), 6.38 (d, *J*=1.7 Hz. 1H), 6.33 (dd, *J*=7.8, 1.7 Hz, 1H), 5.01 (s, 1H), 4.38 (brs, 4H), 3.37 (m, 8H), 1.09 (t, *J*=6.9 Hz, 12H).

^{13}C (DMSO-d6, 100 MHz) δ 146.1, 137.0, 135.1, 133.8, 131.1, 131.0, 127.4, 118.1, 115.6, 114.9, 114.3, 103.2, 45.6, 43.8, 12.2.

7p:

Yield: 30% (purified by RP Flash Chromatography (1% TFA H2O - MeOH gradient))

HRMS: m/z calculated: 479.2321, measured: 479.2326: $[M+H]^+$

NMR:

^{1}H (DMSO-d6, 400 MHz) δ 8.90 (brs, 1H), 7.13 (d, *J*=8.7 Hz, 2H), 7.05 (d, *J*=2.5Hz, 2H), 6.88 (dd, *J*=8.7, 2.5 Hz, 2H), 6.55-6.46 (m, 3H), 5.09 (s, 1H), 4.45 (brs, 2H), 3.36 (m, 8H), 1.09 (t, *J*=6.9 Hz, 12H).

^{13}C (DMSO-d6, 100 MHz) δ 146.2, 143.9, 137.0, 135.3, 131.1, 130.7, 127.1, 120.1, 115.6, 114.8, 114.1, 103.2, 45.4, 43.8, 12.2.

7s:

To a solution of 3 (0.25 mmol) in glacial acetic acid DDQ (0.25 mmol) was added and the reaction mixture was stirred at rt for 5 minutes, followed by HPLC-MS. After that **5s** (0,1 mmol) was added and the solution was stirred at rt for 1h. After the completion was determined by HPLC-MS, the solvent was evaporated. To the residue ethyl acetate and sat. sodium hydrogen carbonate solution was added (to remove the forming 4,5-dichloro-3,6-dihydroxybenzene-1,2-dicarbonitrile). The organic layers

were washed with brine, dried over sodium sulfate, and the solvent was evaporated. The product was purified by RP Flash Chromatography (1% TFA H2O - MeOH gradient).

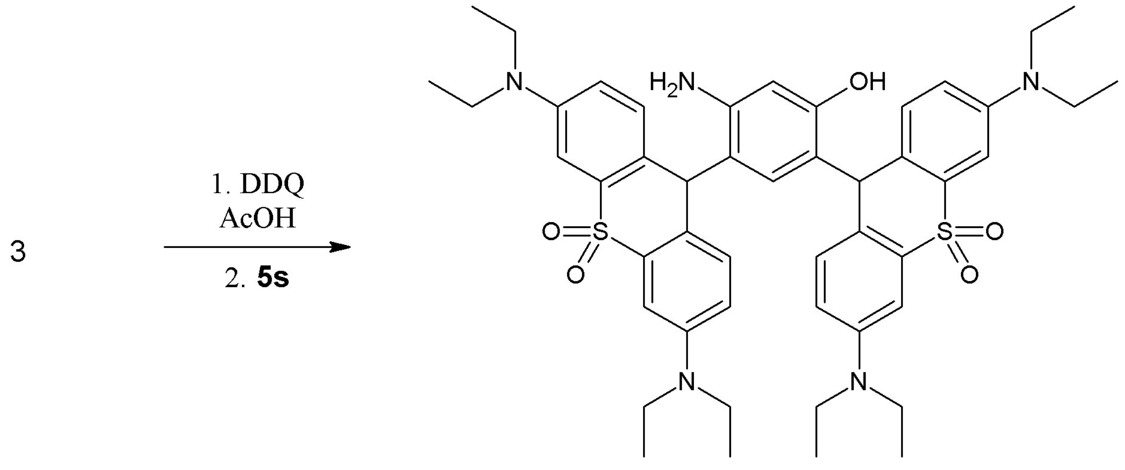

7s

Yield: 43%

HRMS: m/z calculated: 850.1426, measured: 850.1431: $[M+H]^+$

NMR:

^{1}H (DMSO-d6, 400 MHz) δ 7.08-7.02 (m, 4H), 6.96-6.88 (m, 4H), 6.85 (dd, *J*=8.5, 1.8 Hz, 2H), 6.76 (dd, *J*=8.7, 1.9 Hz, 2H), 6.45 (s, 1H), 6.28 (s, 1H), 5.47 (s, 1H), 5.21 (s, 1H), 3.41-3.26 (m, 16H), 1.12-0.97 (m, 24H).

^{13}C (DMSO-d6, 100 MHz) δ 155.8, 147.4, 146.7, 146.2, 137.6, 137.5, 135.3, 130.4, 130.0, 128.7,127.5, 118.1, 118.1, 115.5, 115.1, 104.1, 103.8, 102.5, 44.0, 44.0, 39.9, 38.0, 12.2, 12.2.

7t:

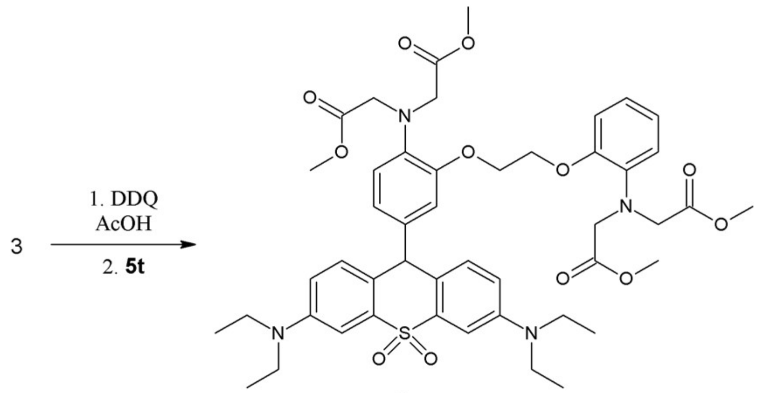

7t

To a solution of **3** (0,35 mmol; 130 mg) in glacial acetic acid was added DDQ (0.35 mmol; 79,5 mg) and the reaction mixture was stirred at rt for 5 minutes, followed by HPLC-MS. After that tetramethyl 2,2',2'',2'''-(((ethane-1,2-diylbis(oxy))bis(2,1-phenylene))bis(azanetriyl)) tetraacetate (0,8 mmol; 149 mg) was added and the reaction mixture was stirred at rt for 1h. The completion was determined by

HPLC-MS. The solvents were evaporated and the residue was purified by RP flash chromatography (1% TFA H2O - MeOH gradient). Yield: 37%.

<u>HRMS</u>: m/z calculated: 903.3850, measured: 903.3870: $[M+H]^+$, m/z 925.3690: $[M+Na]^+$

<u>NMR:</u>

^{1}H NMR (600 MHz, DMSO-d_6) δ 7.27 (d, *J*=8.6 Hz, 2H), 7.15 (s, 2H), 7.00 (s, 1H), 6.95 (d, *J*=8.6 Hz, 2H), 6.88 (d, *J*=7.0 Hz, 1H), 6.86-6.78 (m, 3H), 6.66 (d, *J*=7.0 Hz, 1H), 6.60 (d, *J*=8.2 Hz, 1H), 5.33 (s, 1H), 4.10 (m, 2H), 4.04 (s, 4H), 4.04 (s, 4H), 4.02 (m, 2H), 3.43 (s, 6H), 3.39 (m, 8H), 3.36 (s, 6H), 1.08 (t, *J*=6.9 Hz, 12H)

^{13}C NMR (150 MHz, DMSO-d_6) δ 171.4, 171.3, 149.5, 149.4, 145.9, 138.8, 137.3, 137.1, 136.2, 131.3, 121.1, 121.1, 120.8, 117.7, 117.7,116.6, 116.4, 114.4, 113.2, 113.2, 104.0, 66.9, 66.9, 53.1, 52.9, 51.2, 51.2, 45.8, 44.4, 12.0

<u>8t:</u>

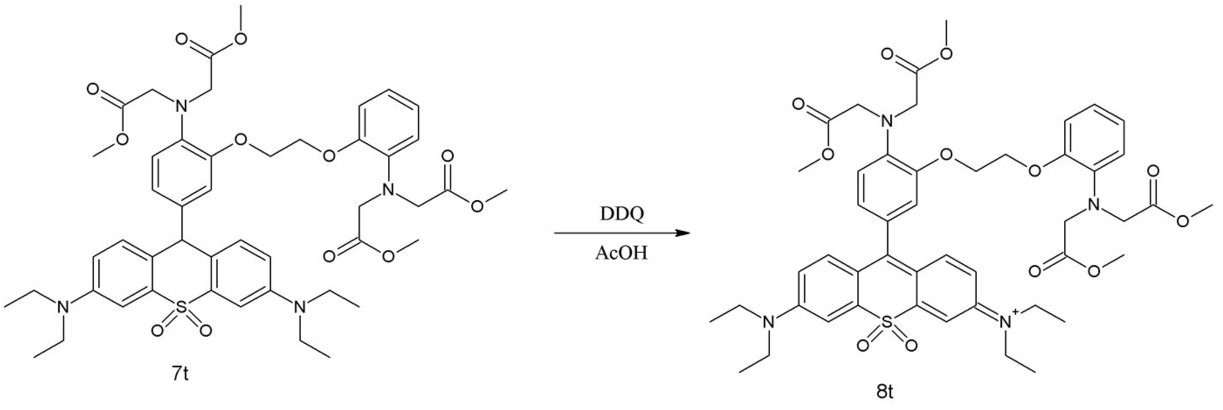

To a solution of 7t (0.1 mmol; 104 mg) in glacial acetic acid was added DDQ (0.1 mmol; 22,7 mg) and the reaction mixture was stirred at rt for 5 minutes. The completion was determined by HPLC-MS. Purified by RP prep HPLC (1% TFA H2O - MeOH gradient). Yield 99%.

<u>HRMS</u>: m/z calculated: 901.3694, measured: 901.3705 M^+, m/z 925.3690: MH^+Na

7u:

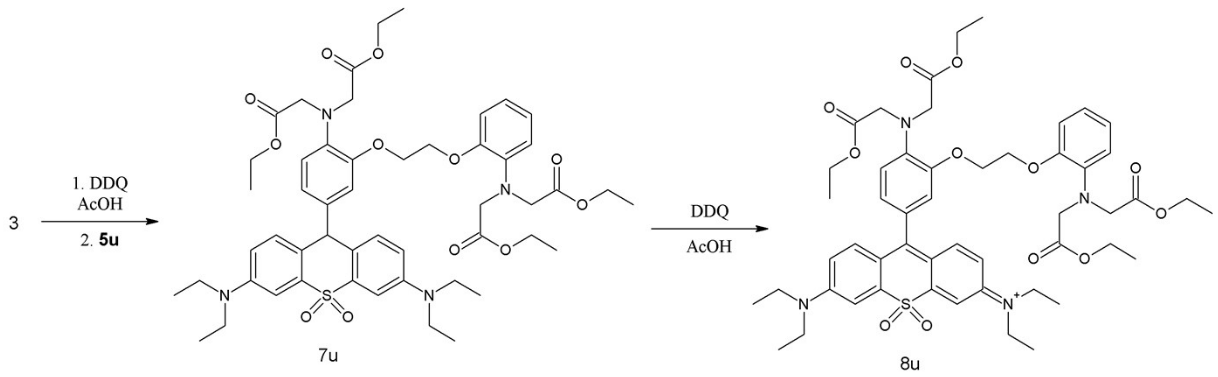

To a solution of **3** (1 eq; 0.6 mmol, 224 mg) in glacial acetic acid was added 2,3-dichloro-5,6-dicyano-p-benzoquinone (1 eq; 0.6 mmol, 136 mg) and the reaction mixture was stirred at rt for 5 minutes, followed by HPLC-MS. After that tetraethyl 2,2',2'',2'''-(((ethane-1,2-diylbis(oxy))bis(2,1-phenylene))bis(azanetriyl)) tetraacetate (0.33 eq. 0,2 mmol, 117 mg) was added and the reaction mixture was stirred at rt for 1h. The completion was determined by HPLC-MS. The solvent were evaporated and the residue was purified by RP flash chromatography (1% TFA H2O - MeOH gradient). Yield: 25%.

NMR:

^{1}H NMR (400 MHz, DMSO-d_6) δ: 7.22 (d, *J*=8.9 Hz, 2H), 7.07 (d, *J*=2.5 Hz, 2H), 7.02 (m, 1H), 6.92-6.85 (m, 3H), 6.85-6.78 (m, 3H), 6.66 (m, 1H), 6.57 (d, *J*= 8.3 Hz, 1H), 5.29 (s, 1H), 4.11 (m, 2H), 4.08-3.98 (m, 10H), 3.90 (q, *J*=7.1 Hz, 4H), 3.85 (q, *J*=7.2 Hz, 4H), 3.37 (m, 8H), 1.09 (t, *J*=6.9 Hz, 12H), 1.02 (t, *J*=7.1 Hz, 6H), 0.95 (t, *J*=7.2 Hz, 6H).

^{13}C NMR (100 MHz, DMSO-d_6) δ: 170.9, 170.8, 149.5, 149.4, 146.3, 138.8, 137.2, 137.0, 136.3, 131.1, 126.2, 121.0, 121.0, 120.7, 117.6, 115.8, 113.1, 113.0, 103.1, 66.9, 66.8, 60.1, 60.1, 53.1, 52.9, 45.7, 43.8, 13.8, 13.8, 12.1.

8u:

To a solution of **7u** (0.104 mmol; 52,7 mg) in glacial acetic acid DDQ (0.104 mmol; 23.6 mg) was added and the reaction mixture was stirred at rt for 5 minutes. The completion was determined by HPLC-MS. Purified by RP prep HPLC (1% TFA H2O - MeOH gradient). Yield 99%.

NMR:

^{1}H NMR (400 MHz, CD_3OD) δ: 7.39 (d, *J*=9.1 Hz, 2H), 7.16 (m, 2H), 7.10-7.01 (m, 2H), 6.96 (dm, *J*=9.1 Hz, 2H), 6.89-6.77 (m, 3H), 6.71 (d, *J*=7.5 Hz, 1H), 6.63 (d, *J*=8.3 Hz, 1H), 4.11 (m, 2H), 4.08-3.98 (m, 10H), 3.92 (q, *J*=7.1 Hz, 4H), 3.81 (q, *J*=7.1 Hz, 4H), 3.45 (q, *J*=6.7 Hz, 8H), 1.19 (t, *J*=6.7 Hz, 12H), 1.05 (t, *J*=7.1 Hz, 6H), 0.97 (t, *J*=7.1 Hz, 6H).

^{13}C NMR (100 MHz, CD_3OD) δ: 173.5, 173.4, 151.5, 151.0, 148.8, 142.4, 140.6, 139.2, 139.0, 131.5, 126.9, 122.8, 122.1, 119.1, 118.7, 118.6, 117.8, 113.6, 111.8, 103.5, 78.7, 68.2, 68.2, 62.0, 62.0, 54.6, 54.4, 45.4, 14.3, 14.3, 12.7.

1: General procedure of the hydrolysis:

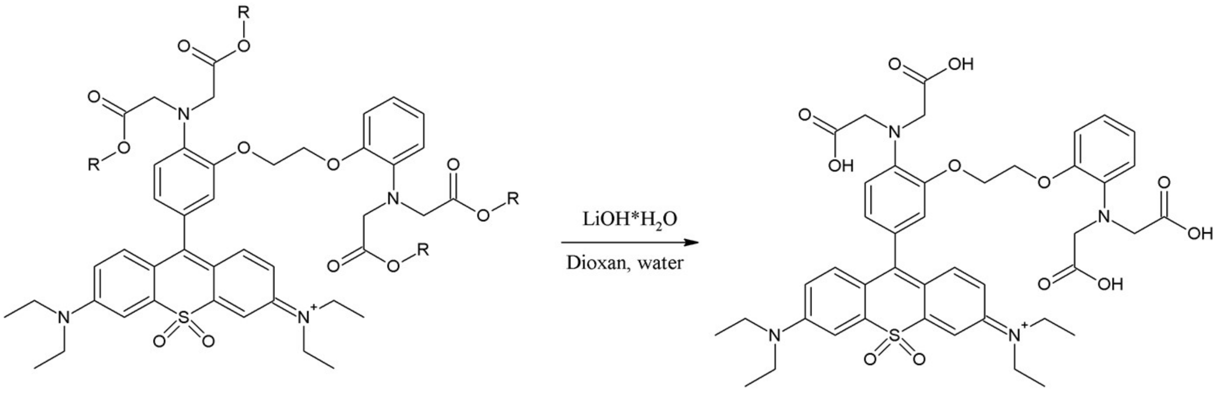

8t: R: Me

8u: R: Et

1

To a solution of **8t** or **8u** (0,07 mmol) in dioxan - water was added lithium hydroxide monohydrate (0.07 mmol) and the reaction mixture was stirred at rt. The completion was determined by HPLC-MS. The solvent was evaporated. Purified by RP prep HPLC (1% TFA H2O - MeOH gradient). Yield 99%.

HRMS: m/z calculated: 845.3068, measured: 845.3092: M^+, possible fragment ion MS: m/z 787.3039: $[M\text{-}C2H2O2]^+$

NMR:

^1H (CD_3CN, 500 MHz) δ 7.62 (d, *J*=2.5 Hz, 2H), 7.42 (d, *J*=9.7 Hz, 2H), 7.04-6.85 (m, 9H), 4.35-4.28 (m, 4H), 4.20 (s, 4H), 3.99 (s, 4H), 3.73 (m, 8H), 1.28 (t, *J*=7.0 Hz, 12H).

^{13}C (CD_3CN, 125 MHz) δ 174.8, 174.5, 160.3, 155.0, 151.8, 150.2, 145.3, 141.2, 141.1, 139.0, 128.1, 124.7, 124.3, 122.0, 120.3, 119.9, 117.9, 116.6, 116.5, 114.2, 112.5, 68.6, 67.8, 57.1, 56.8, 47.6, 13.1.

3:

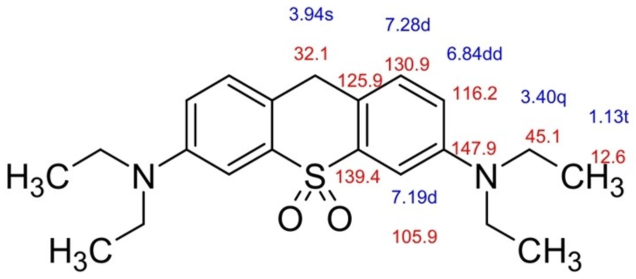
3.94s
7.28d
6.84dd
32.1
125.9
130.9
116.2
3.40q
1.13t
45.1
147.9
12.6
139.4
7.19d
105.9

[1]H:

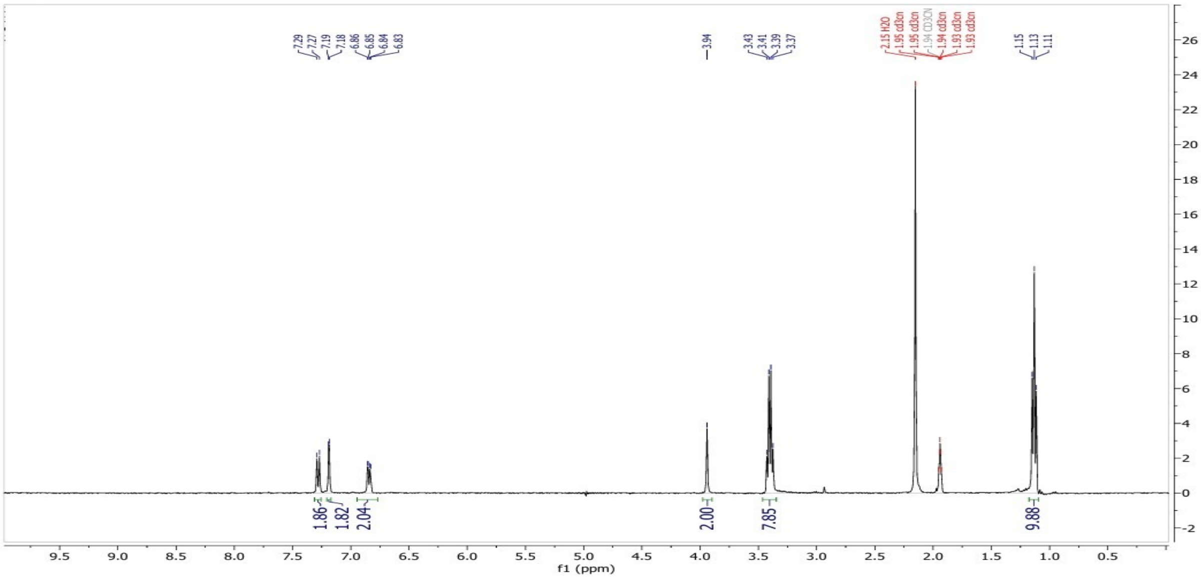

[13]C:

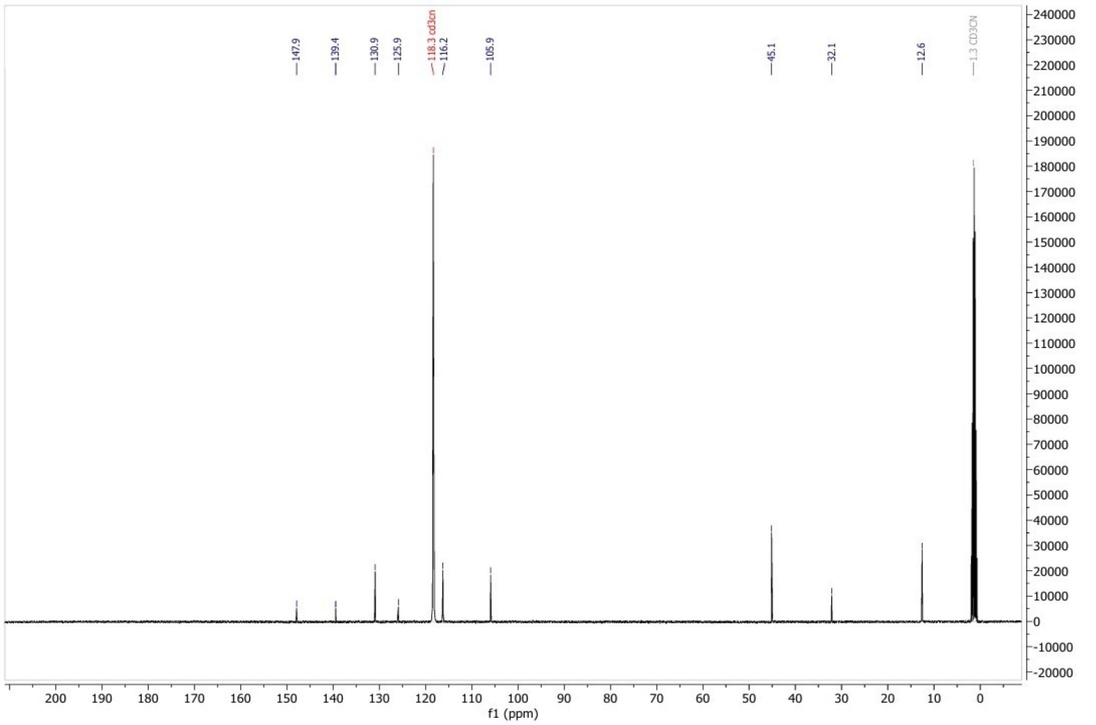

7b:

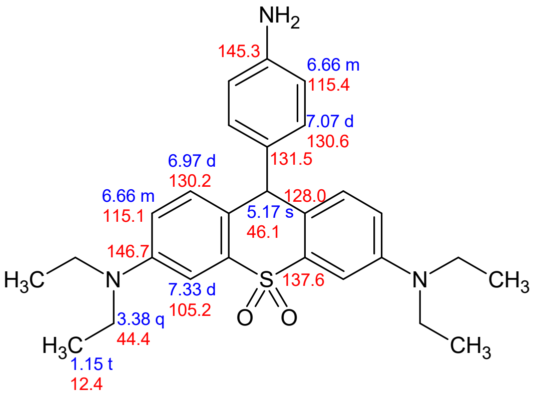
NH2
145.3
6.66 m
115.4
7.07 d
130.6
131.5
6.97 d
130.2
6.66 m
115.1
128.0
5.17 s
46.1
146.7
H3C
N
S
137.6
N
CH3
7.33 d
105.2
3.38 q
44.4
O
O
H3C
1.15 t
12.4
CH3

^{1}H:

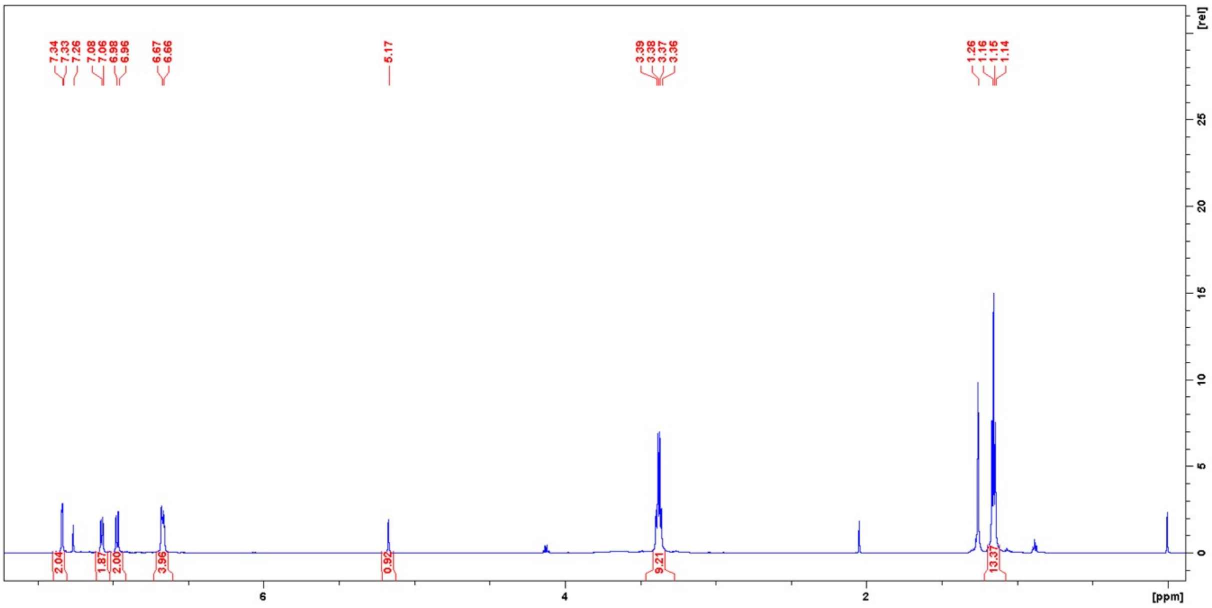

^{13}C:

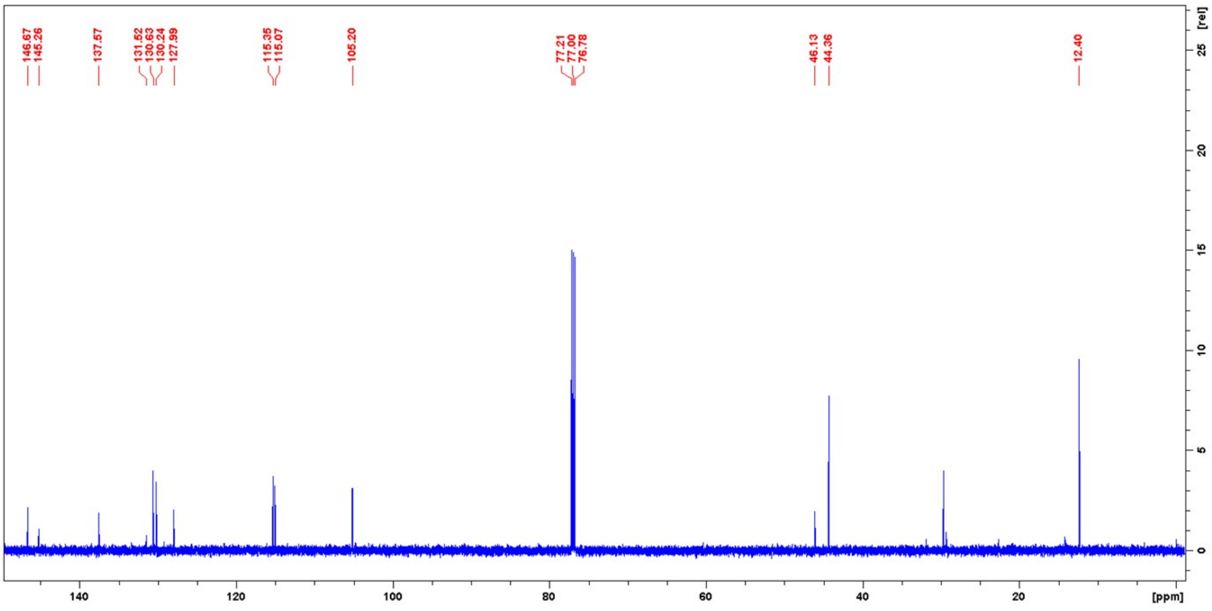

7a:

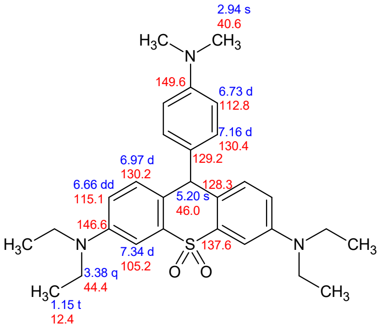
2.94 s
40.6
149.6
6.73 d
112.8
7.16 d
130.4
129.2
6.97 d
130.2
6.66 dd
115.1
128.3
5.20 s
46.0
146.6
7.34 d
105.2
137.6
3.38 q
44.4
1.15 t
12.4

^{1}H:

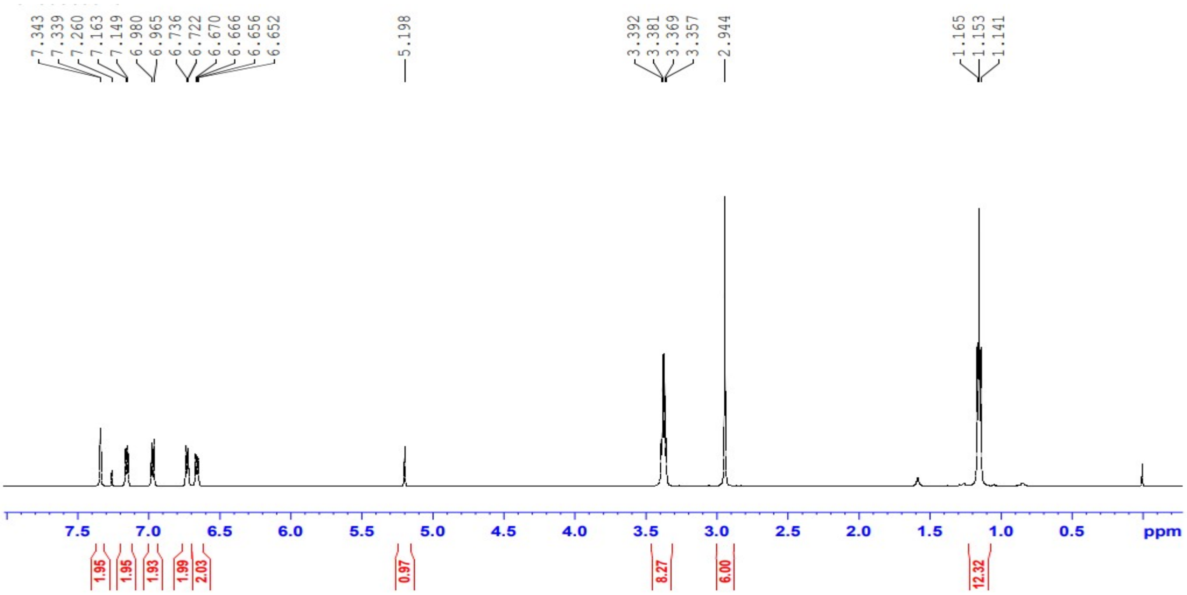
7.343
7.339
7.260
7.163
7.149
6.980
6.965
6.736
6.722
6.670
6.666
6.656
6.652
5.198
3.392
3.381
3.369
3.357
2.944
1.165
1.153
1.141
7.5
7.0
6.5
6.0
5.5
5.0
4.5
4.0
3.5
3.0
2.5
2.0
1.5
1.0
0.5
ppm
1.95
1.95
1.93
1.99
2.03
0.97
8.27
6.00
12.32

^{13}C:

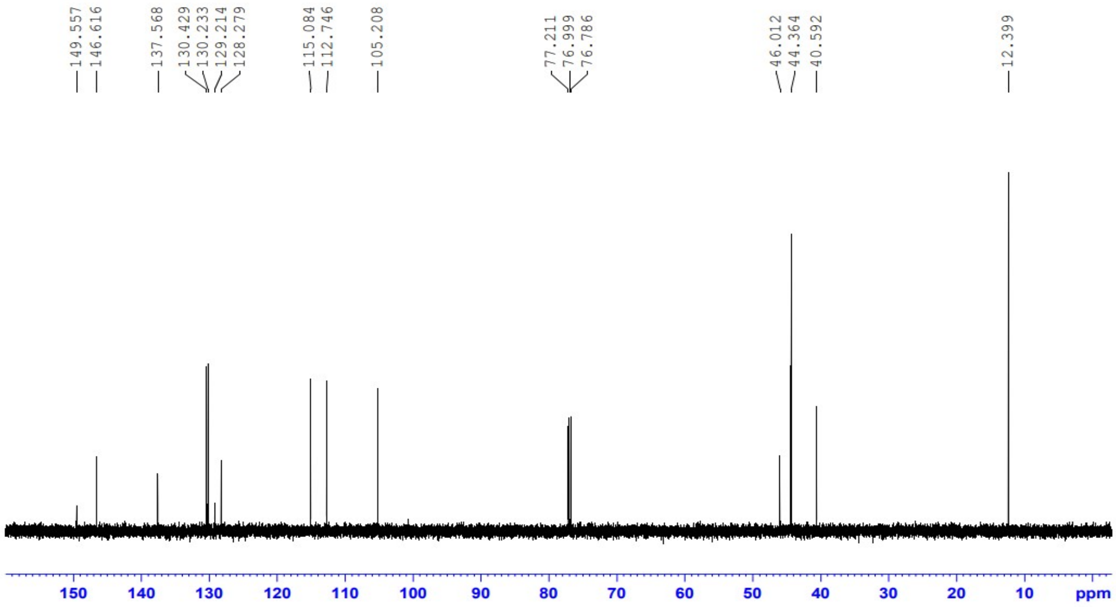
149.557
146.616
137.568
130.429
130.233
129.214
128.279
115.084
112.746
105.208
77.211
76.999
76.786
46.012
44.364
40.592
12.399
150
140
130
120
110
100
90
80
70
60
50
40
30
20
10
ppm

7c:

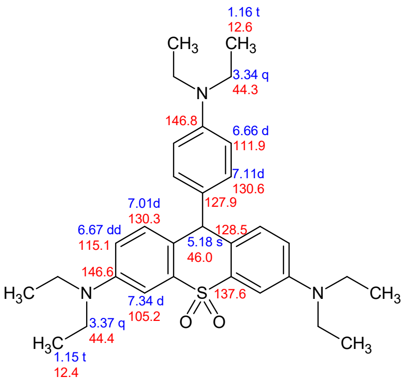
1.16 t
12.6
CH3
CH3
3.34 q
44.3
N
146.8
6.66 d
111.9
7.11d
130.6
7.01d
130.3
127.9
6.67 dd
115.1
128.5
5.18 s
46.0
146.6
H3C
N
7.34 d
105.2
S
137.6
N
CH3
3.37 q
44.4
O
O
H3C
1.15 t
12.4
CH3

^{1}H:

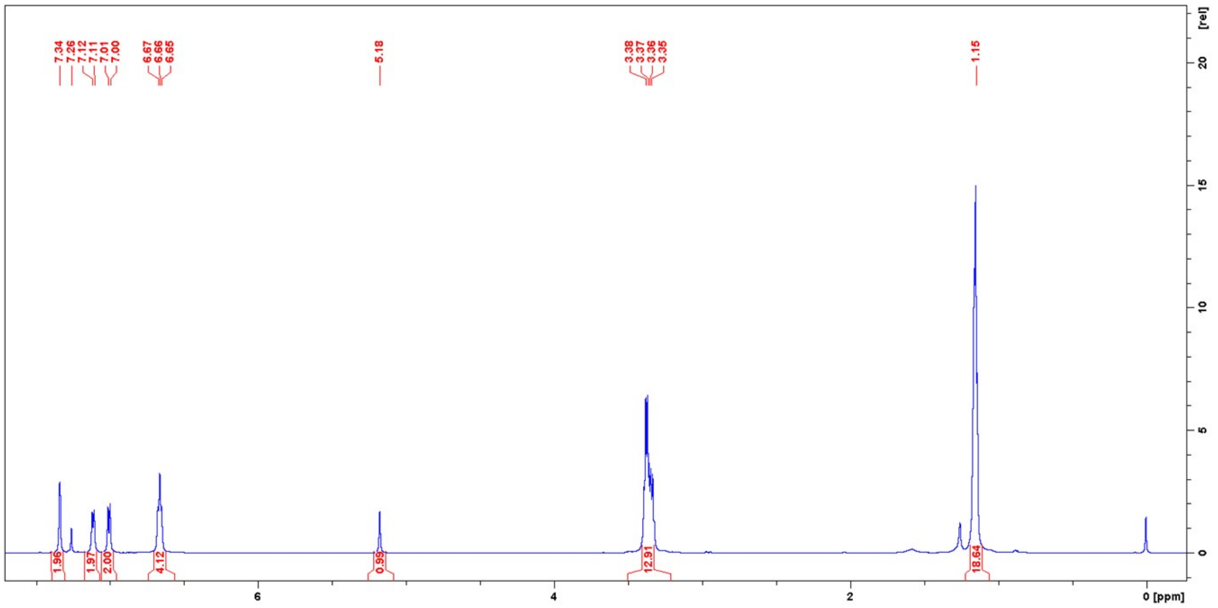
7.34
7.26
7.12
7.11
7.01
7.00
6.67
6.66
6.65
5.18
3.38
3.37
3.36
3.35
1.15
1.96
1.97
2.00
4.12
0.99
12.91
18.64
[rel]
6
4
2
0 [ppm]

^{13}C:

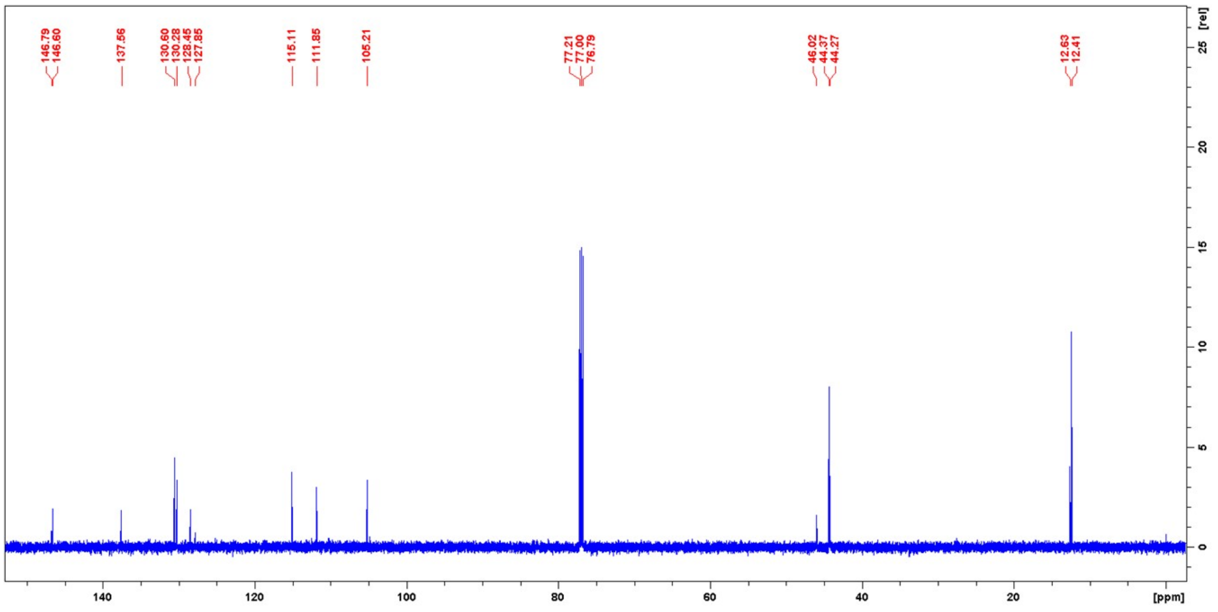
146.79
146.60
137.56
130.60
130.28
128.45
127.85
115.11
111.85
105.21
77.21
77.00
76.79
46.02
44.37
44.27
12.63
12.41
[rel]
140
120
100
80
60
40
20
[ppm]

7d:

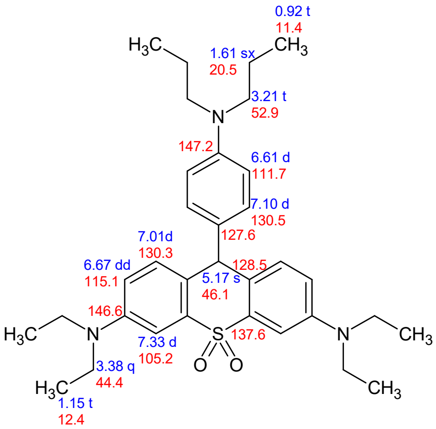

0.92 t
11.4
1.61 sx
20.5
3.21 t
52.9
147.2
6.61 d
111.7
7.10 d
130.5
127.6
7.01d
130.3
6.67 dd
115.1
128.5
5.17 s
46.1
146.6
137.6
7.33 d
105.2
3.38 q
44.4
1.15 t
12.4

^{1}H:

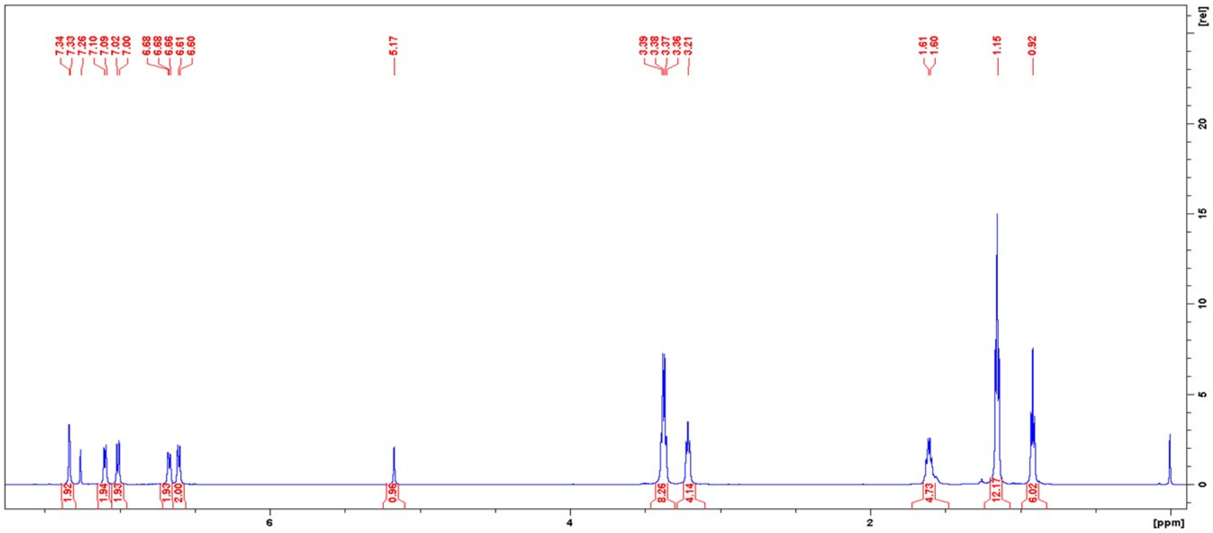

7.34
7.33
7.26
7.10
7.09
7.02
7.00
6.68
6.68
6.66
6.61
6.60
5.17
3.39
3.38
3.37
3.36
3.21
1.61
1.60
1.15
0.92
1.92
1.94
1.93
1.93
2.00
0.96
8.26
4.14
4.73
12.17
6.02
[ppm]
[rel]

^{13}C:

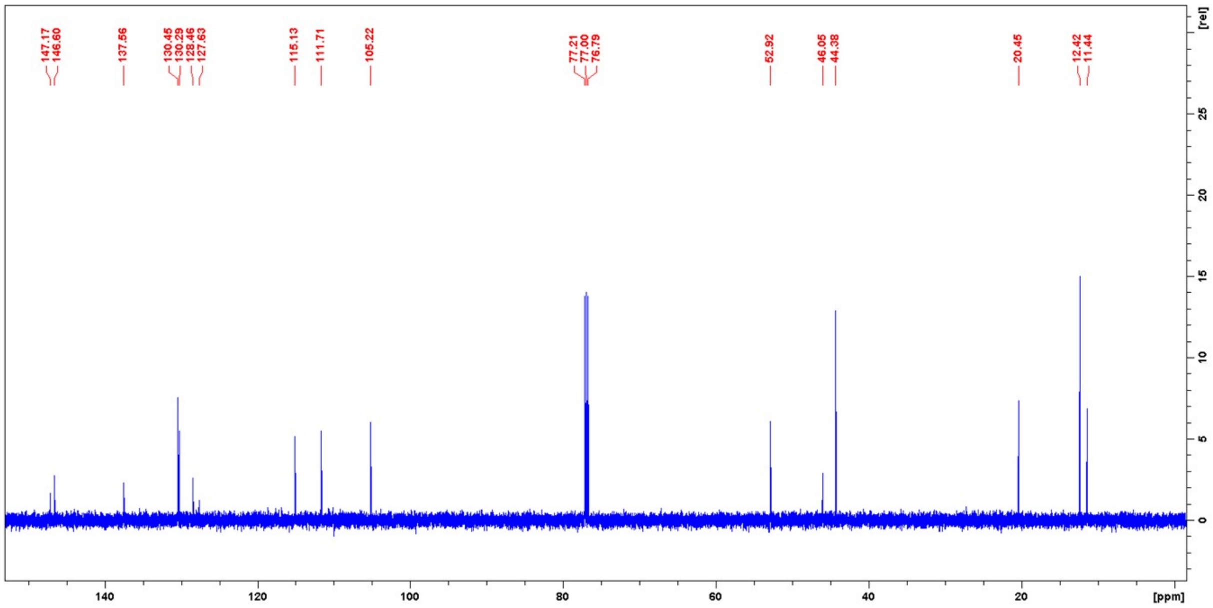

147.17
146.60
137.56
130.45
130.29
128.46
127.63
115.13
111.71
105.22
77.21
77.00
76.79
52.92
46.05
44.38
20.45
12.42
11.44
[ppm]
[rel]

7e:

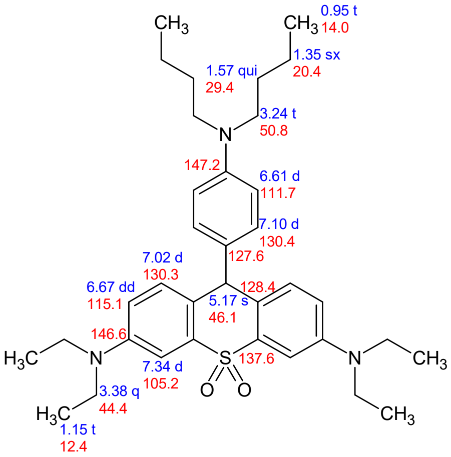

0.95 t
14.0
1.35 sx
20.4
1.57 qui
29.4
3.24 t
50.8
147.2
6.61 d
111.7
7.10 d
130.4
127.6
7.02 d
130.3
6.67 dd
115.1
128.4
5.17 s
46.1
146.6
7.34 d
105.2
137.6
3.38 q
44.4
1.15 t
12.4

^{1}H:

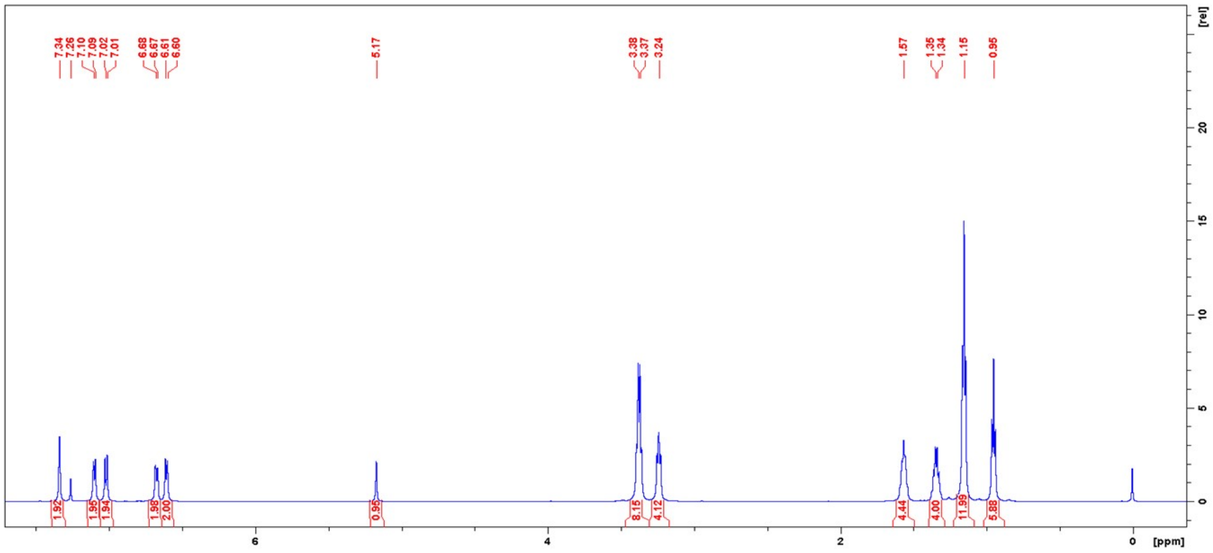

^{13}C:

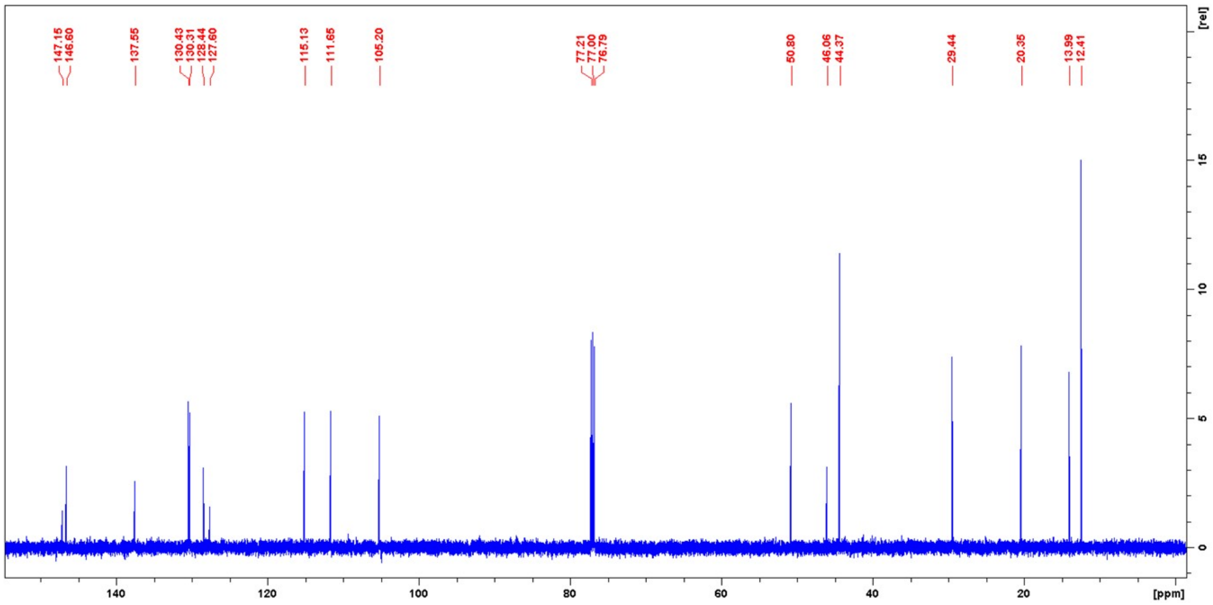

7h:

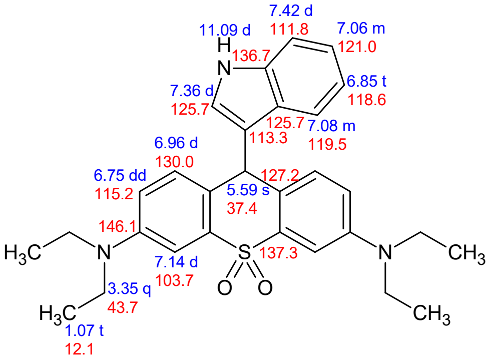

7.42 d
111.8
11.09 d
7.06 m
121.0
136.7
7.36 d
125.7
6.85 t
118.6
125.7
7.08 m
113.3
119.5
6.96 d
130.0
6.75 dd
127.2
115.2
5.59 s
37.4
146.1
7.14 d
137.3
3.35 q
103.7
43.7
1.07 t
12.1

^{1}H:

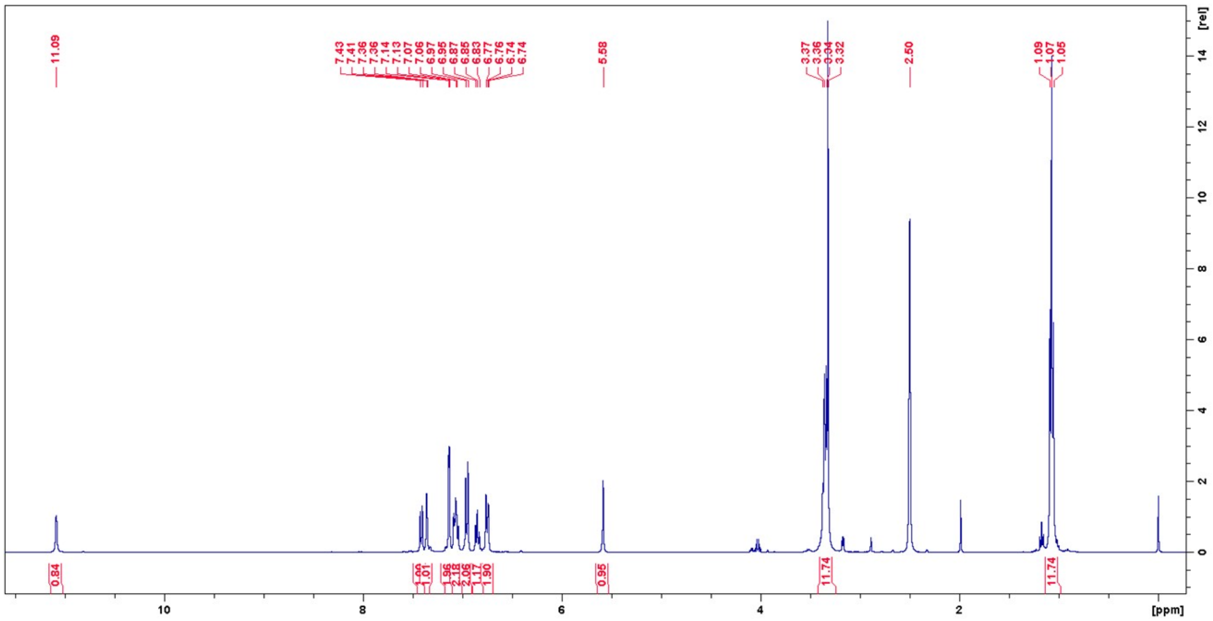

11.09
5.58
2.50
0.84
0.95
11.74
11.74
[ppm]
[rel]

^{13}C:

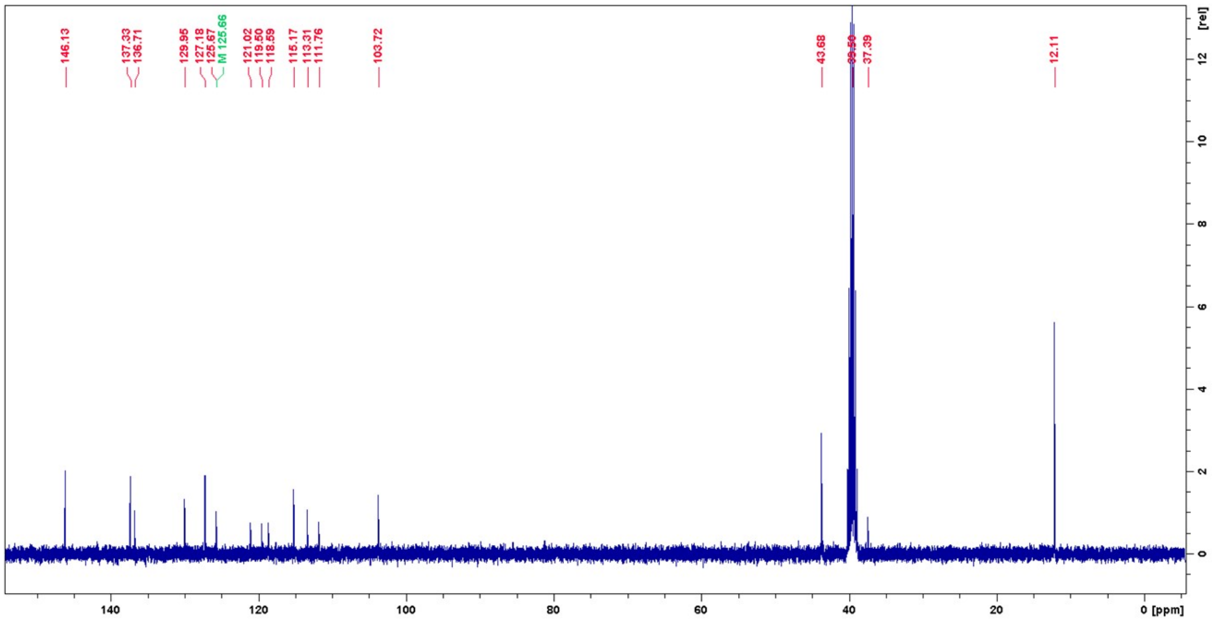

146.13
137.33
136.71
129.95
127.18
125.67
M 125.66
121.02
119.50
118.59
115.17
113.31
111.76
103.72
43.68
37.39
12.11
[ppm]
[rel]

7j:

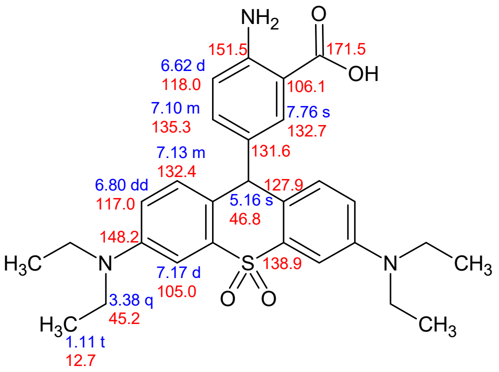
NH2
O
151.5
171.5
6.62 d
118.0
OH
106.1
7.10 m
135.3
7.76 s
132.7
7.13 m
131.6
132.4
6.80 dd
127.9
117.0
5.16 s
46.8
148.2
H3C
N
S
138.9
N
CH3
7.17 d
105.0
3.38 q
45.2
O
O
H3C
CH3
1.11 t
12.7

^{1}H:

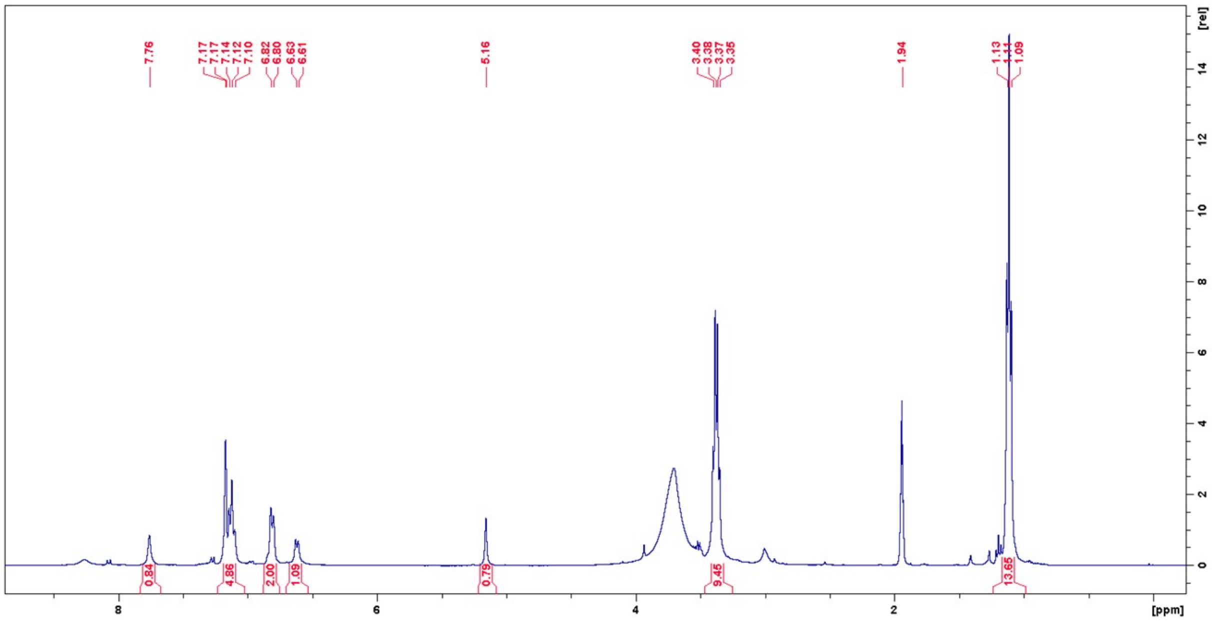
7.76
7.17
7.17
7.14
7.12
7.10
6.82
6.80
6.63
6.61
5.16
3.40
3.38
3.37
3.35
1.94
1.13
1.11
1.09
0.84
4.86
2.00
1.09
0.79
9.45
13.65
8
6
4
2
[ppm]
[rel]

^{13}C:

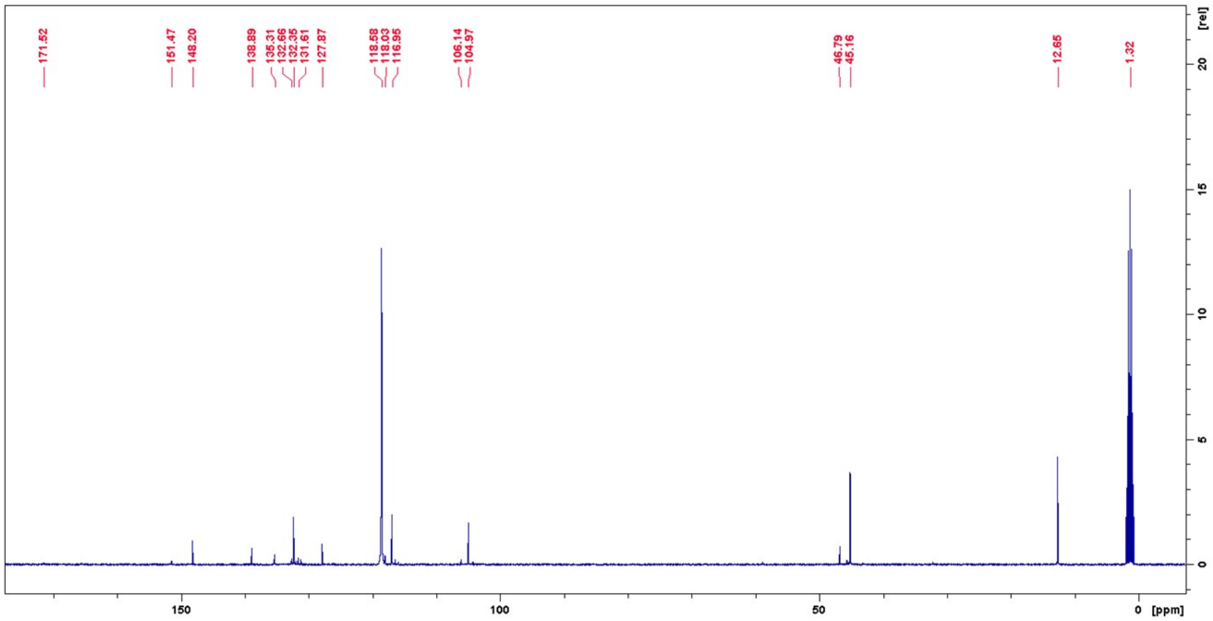
171.52
151.47
148.20
138.89
135.31
132.66
132.35
131.61
127.87
118.58
118.03
116.95
106.14
104.97
46.79
45.16
12.65
1.32
150
100
50
0
[ppm]
[rel]

7n:

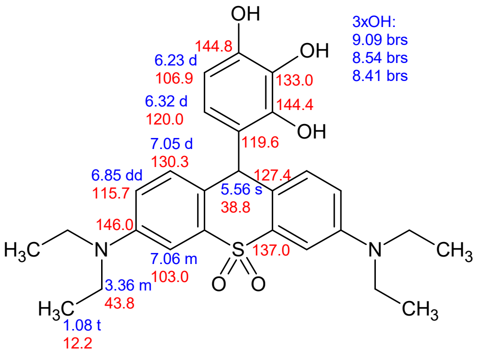
OH
OH
OH
3xOH:
9.09 brs
8.54 brs
8.41 brs
144.8
6.23 d
106.9
133.0
6.32 d
120.0
144.4
7.05 d
130.3
119.6
6.85 dd
115.7
127.4
5.56 s
38.8
146.0
137.0
7.06 m
103.0
3.36 m
43.8
1.08 t
12.2

^{1}H:

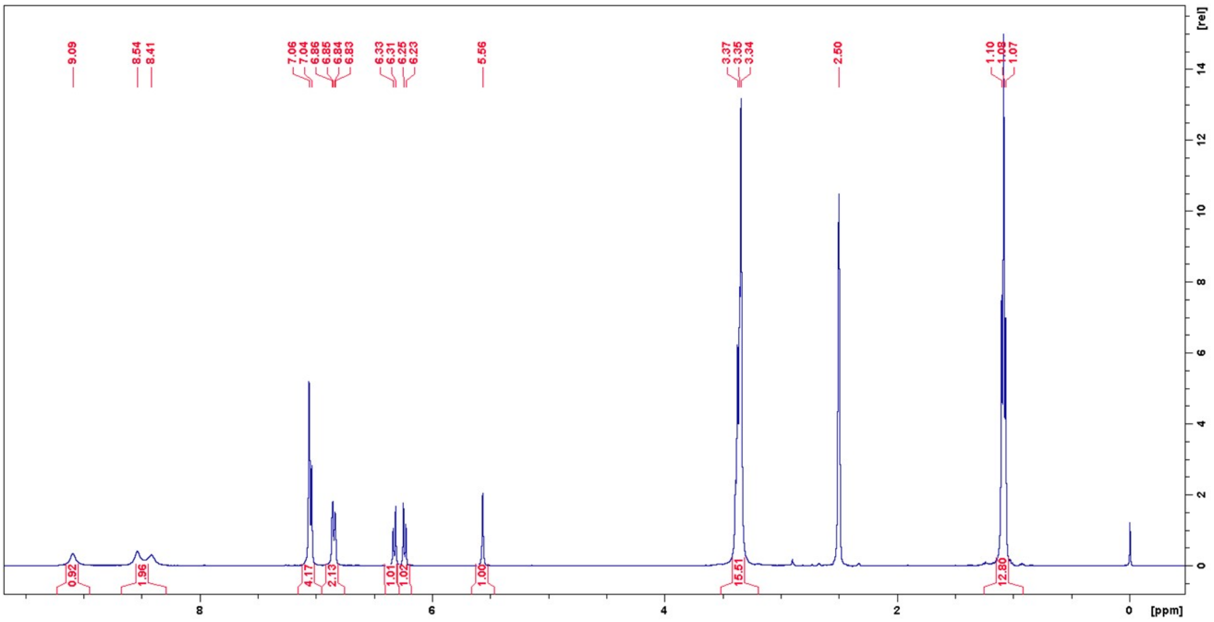

^{13}C:

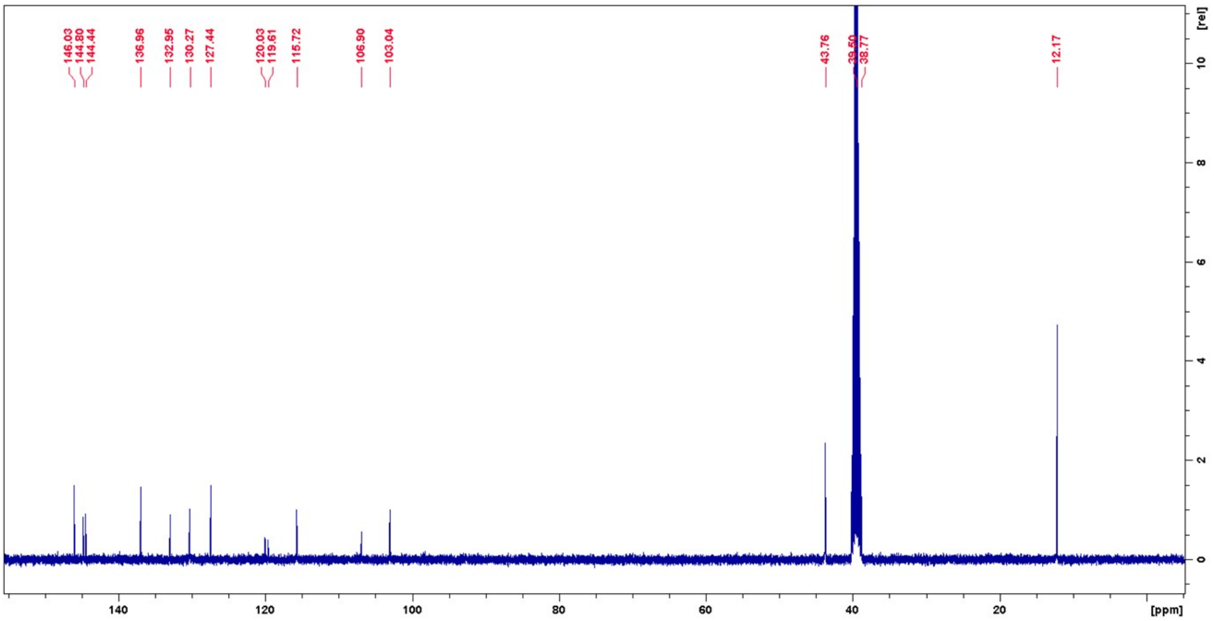

7o:

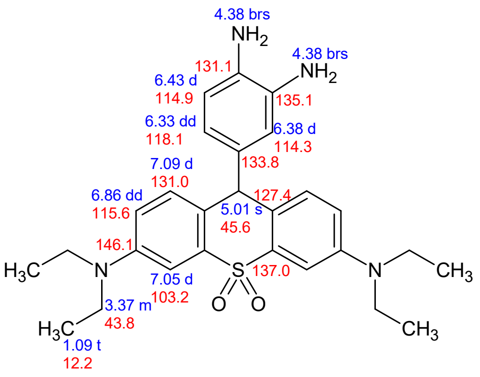

4.38 brs
NH2
4.38 brs
NH2
131.1
6.43 d
114.9
135.1
6.33 dd
118.1
6.38 d
114.3
133.8
7.09 d
131.0
6.86 dd
115.6
127.4
5.01 s
45.6
146.1
H3C
N
7.05 d
103.2
S
137.0
N
CH3
O
O
3.37 m
43.8
H3C
1.09 t
12.2
CH3

^{1}H:

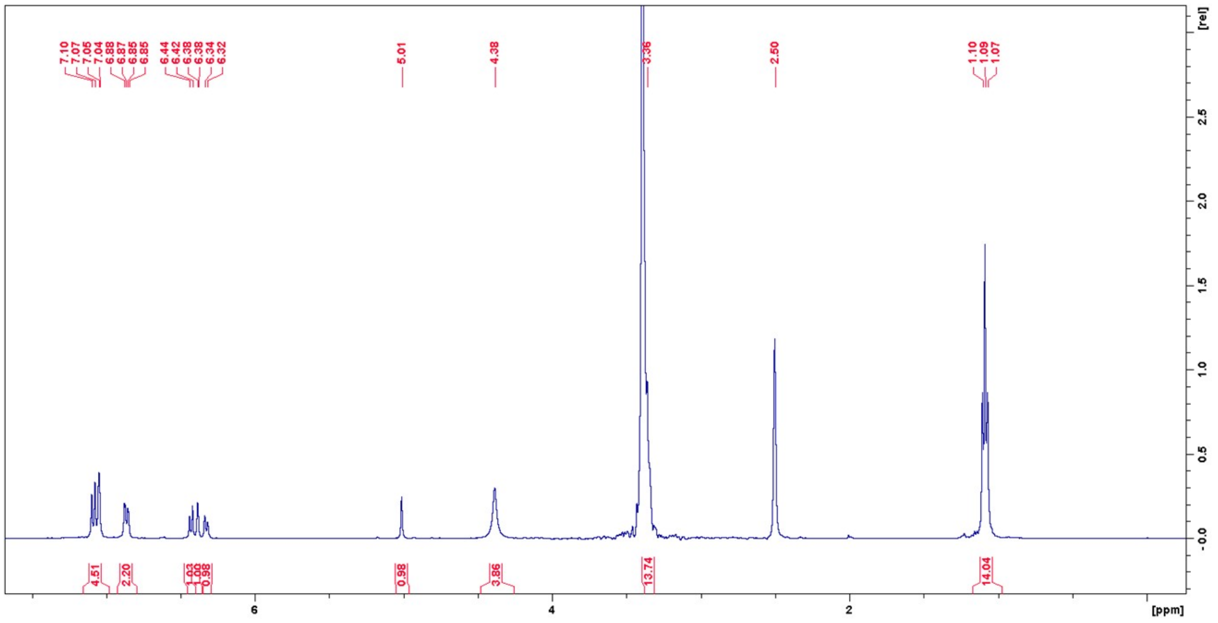

7.10
7.07
7.05
7.04
6.88
6.87
6.85
6.85
6.44
6.42
6.38
6.38
6.34
6.32
5.01
4.38
3.36
2.50
1.10
1.09
1.07
4.51
2.20
1.03
1.00
0.98
0.98
3.86
13.74
14.04
6
4
2
[ppm]
[rel]

^{13}C:

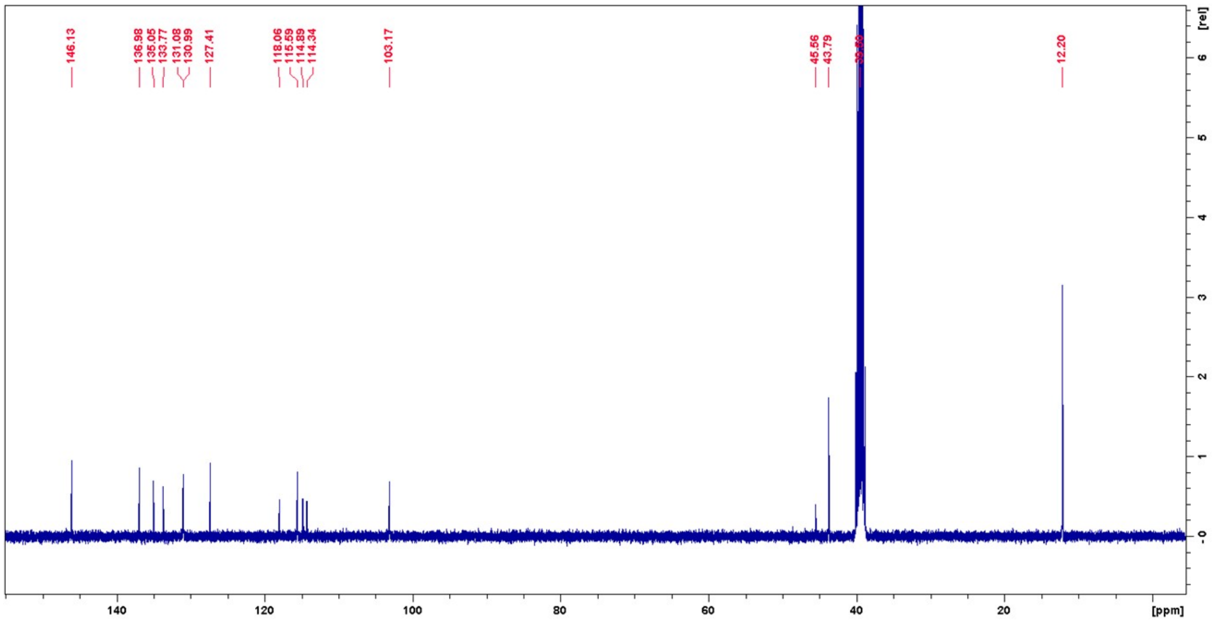

146.13
136.98
135.05
133.77
131.08
130.99
127.41
118.06
115.59
114.89
114.34
103.17
45.56
43.79
12.20
140
120
100
80
60
40
20
[ppm]
[rel]

7p:

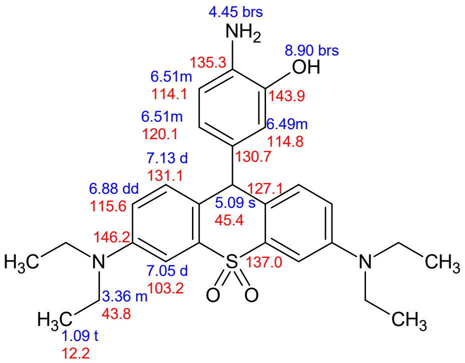
4.45 brs
NH_2
8.90 brs
OH
135.3
6.51m
114.1
143.9
6.51m
120.1
6.49m
114.8
7.13 d
131.1
130.7
6.88 dd
115.6
127.1
5.09 s
45.4
146.2
H_3C
N
S
137.0
N
CH_3
7.05 d
103.2
3.36 m
43.8
O
O
H_3C
CH_3
1.09 t
12.2

^1H:

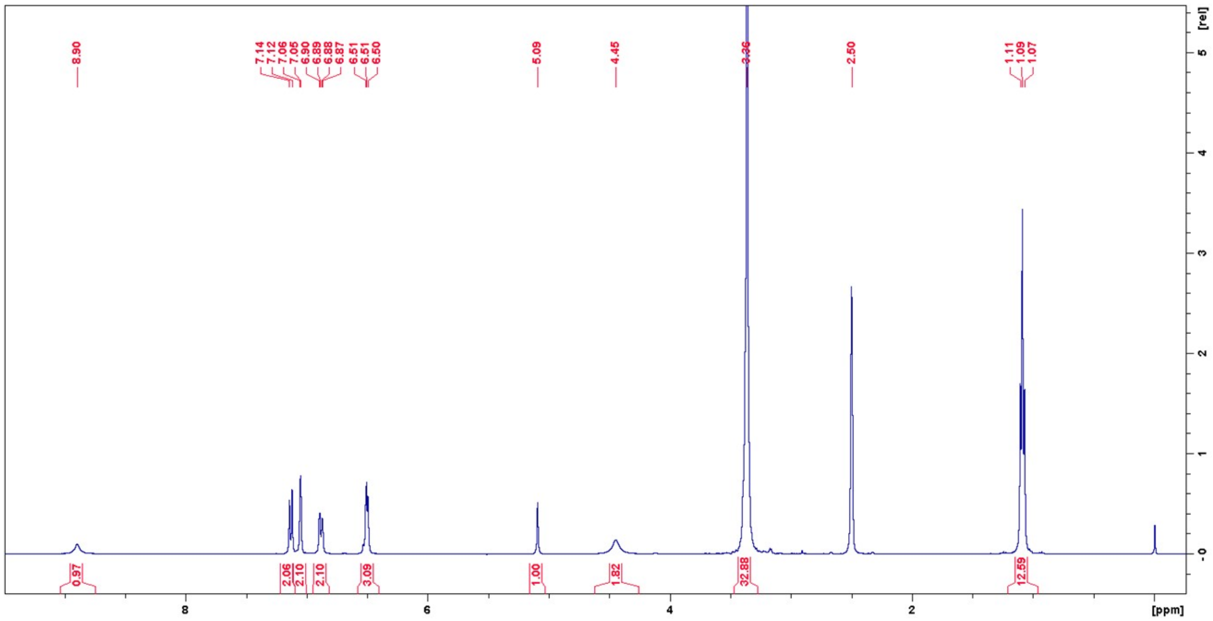

^{13}C:

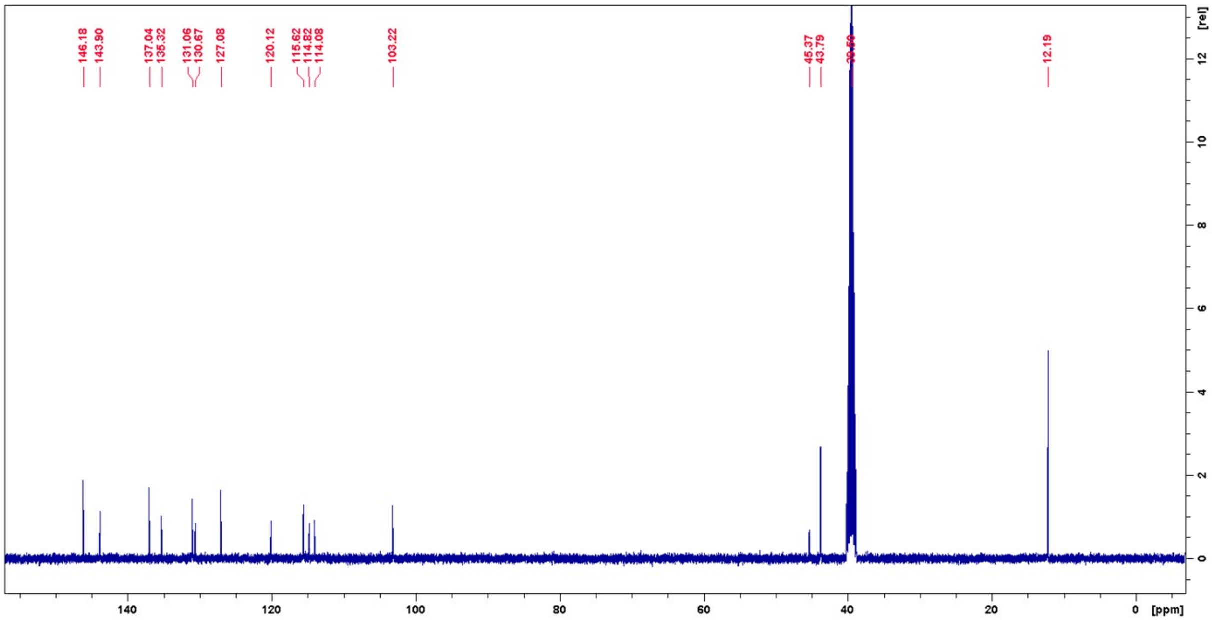

7s:

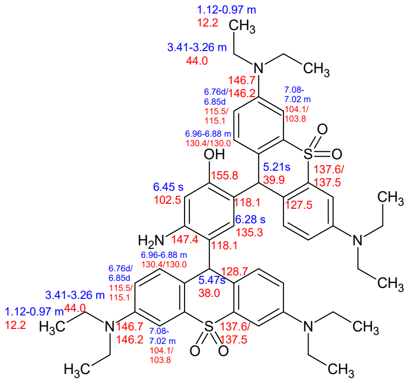

^{1}H:

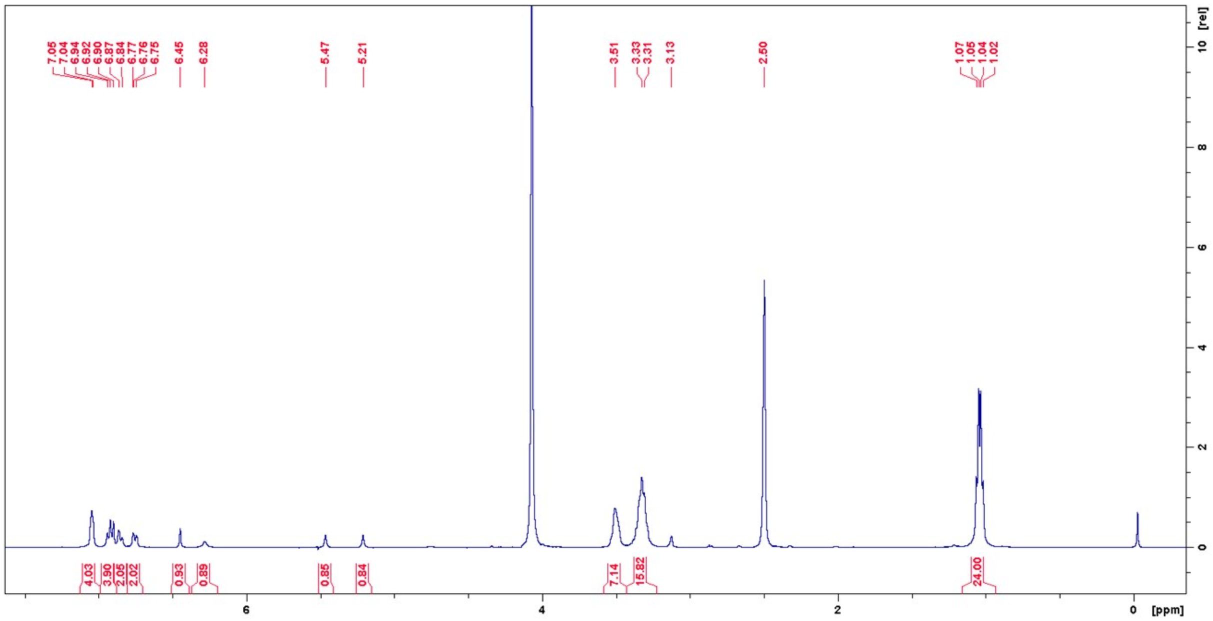
7.05
7.04
6.94
6.92
6.90
6.87
6.84
6.77
6.76
6.75
6.45
6.28
5.47
5.21
3.51
3.33
3.31
3.13
2.50
1.07
1.05
1.04
1.02
4.03
3.90
2.05
2.02
0.93
0.89
0.85
0.84
7.14
15.82
24.00
[rel]
[ppm]

^{13}C:

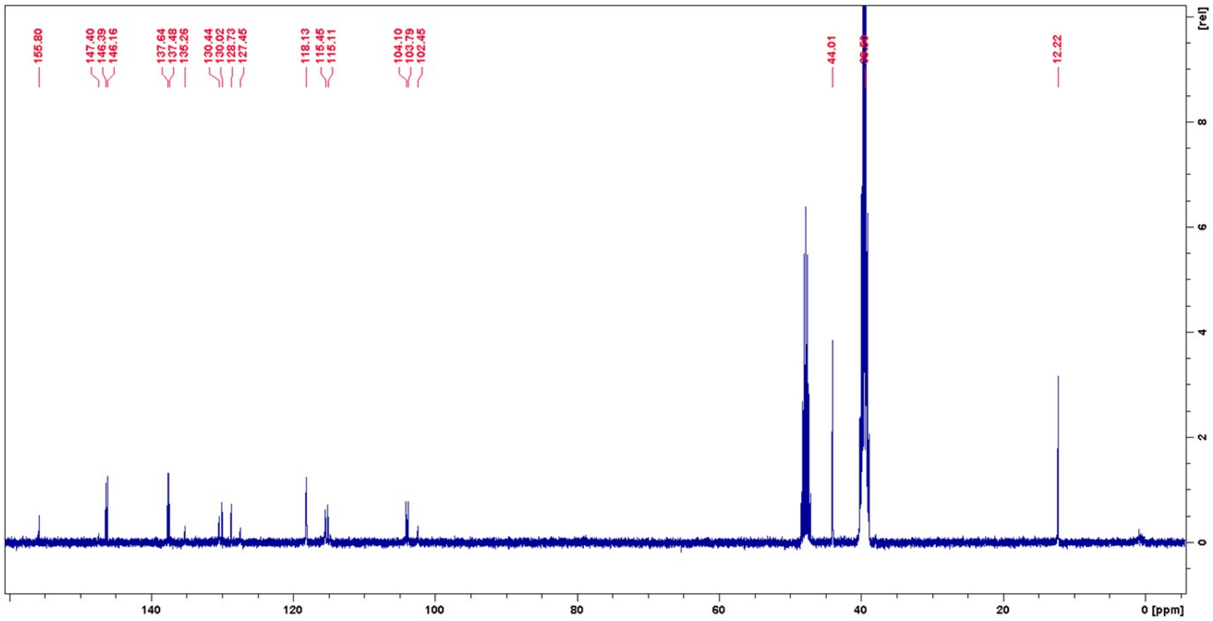
155.80
147.40
146.39
146.16
137.64
137.48
135.26
130.44
130.02
128.73
127.45
118.13
115.45
115.11
104.10
103.79
102.45
44.01
12.22
[rel]
[ppm]

7t:

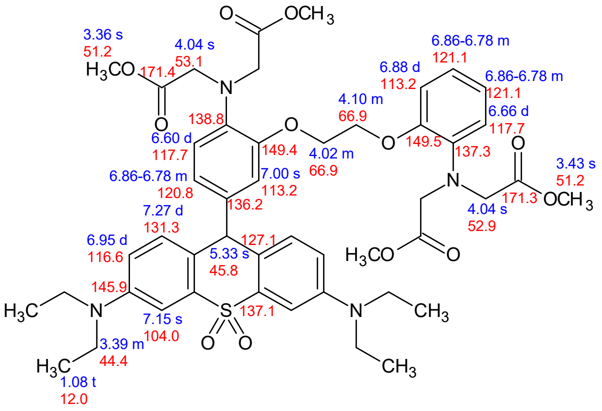

^{1}H:

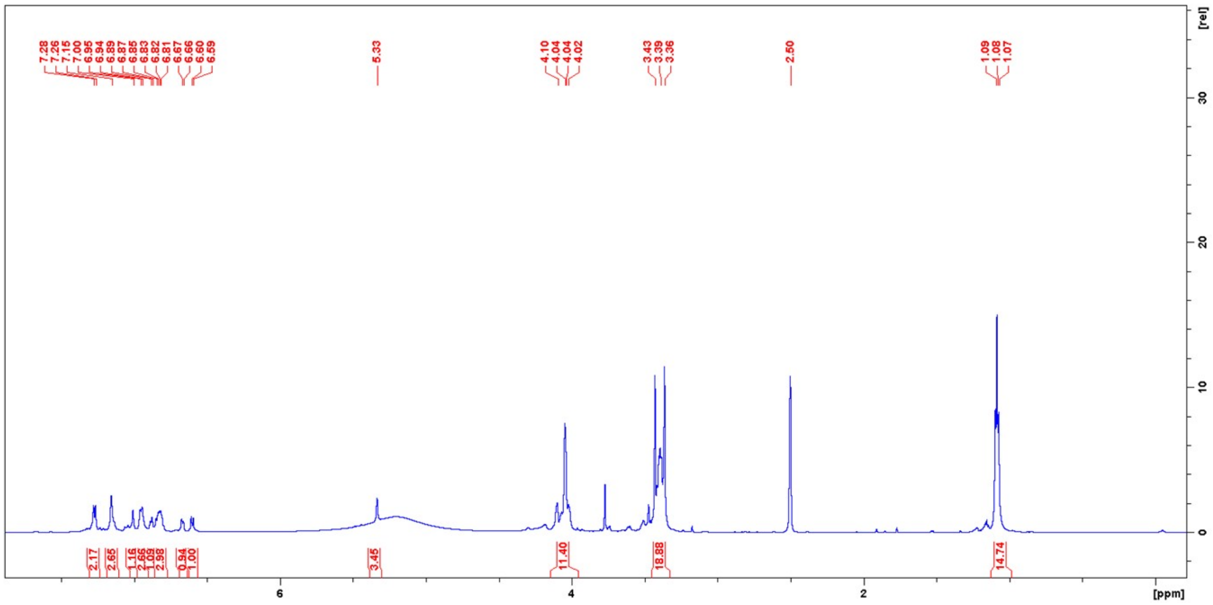

^{13}C:

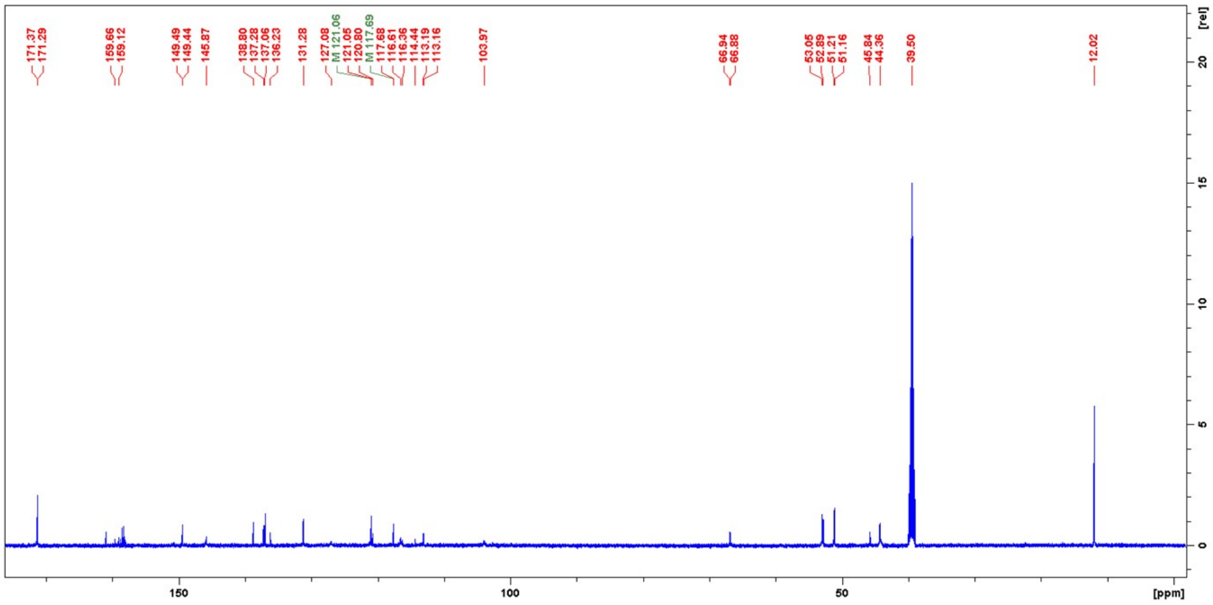

7u:

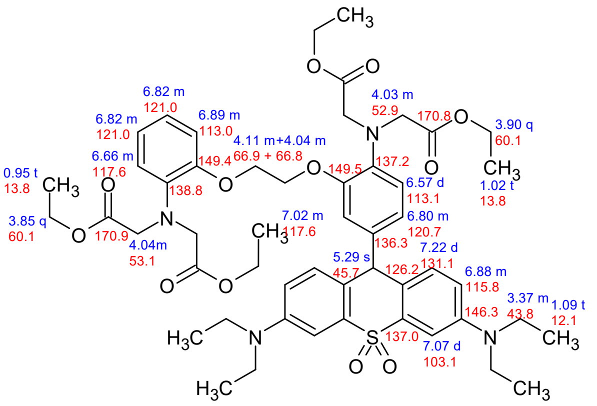

^{1}H:

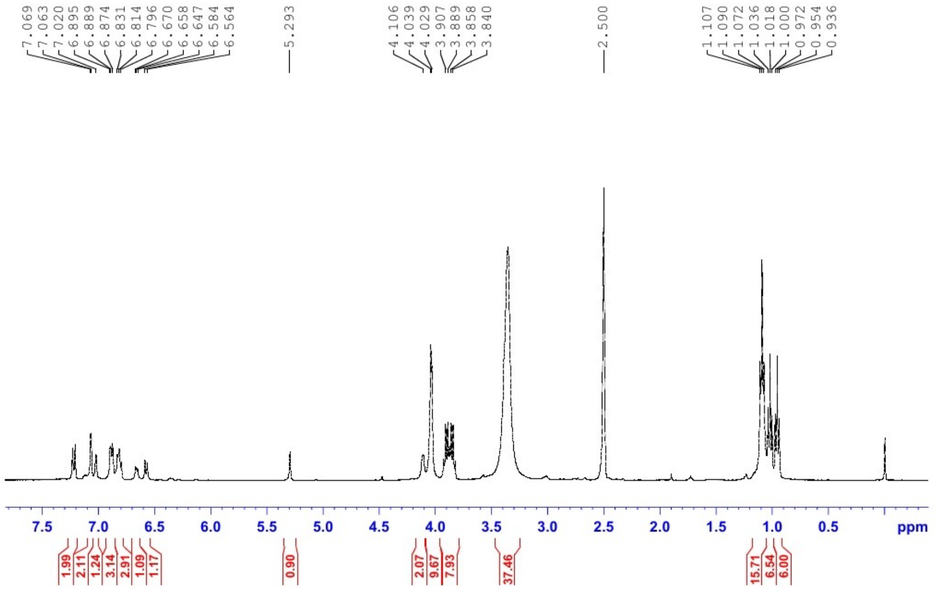
7.069
7.063
7.020
6.895
6.889
6.874
6.831
6.814
6.796
6.670
6.658
6.647
6.584
6.564
5.293
4.106
4.039
4.029
3.907
3.889
3.858
3.840
2.500
1.107
1.090
1.072
1.036
1.018
1.000
0.972
0.954
0.936
ppm
1.99
2.11
1.24
3.14
2.91
1.09
1.17
0.90
2.07
9.67
7.93
37.46
15.71
6.54
6.00

^{13}C:

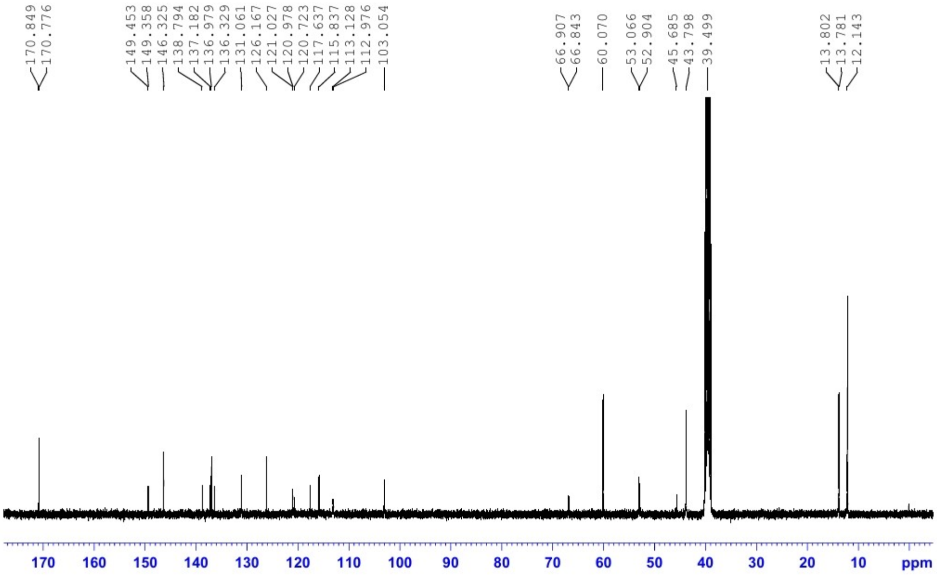
170.849
170.776
149.453
149.358
146.325
138.794
137.182
136.979
136.329
131.061
126.167
121.027
120.978
120.723
117.637
115.837
113.128
112.976
103.054
66.907
66.843
60.070
53.066
52.904
45.685
43.798
39.499
13.802
13.781
12.143
ppm

8u:

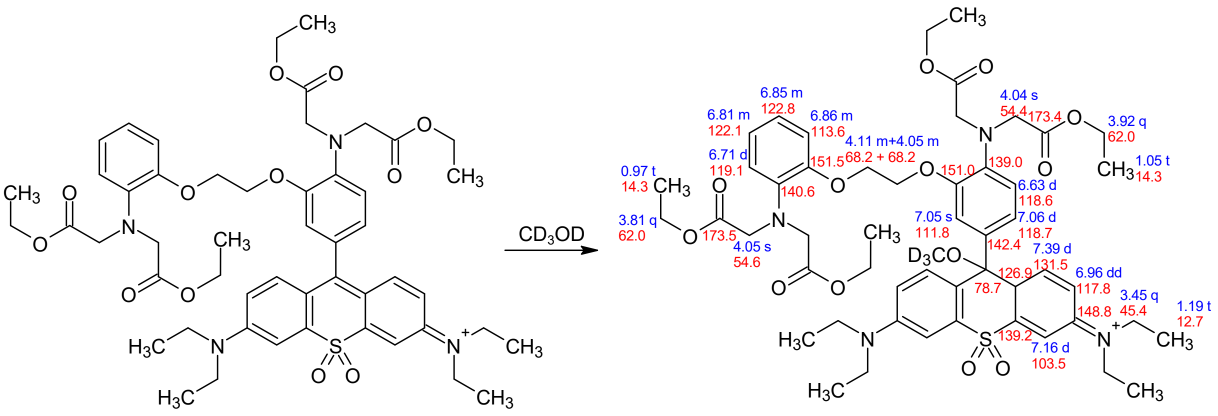

^1H:

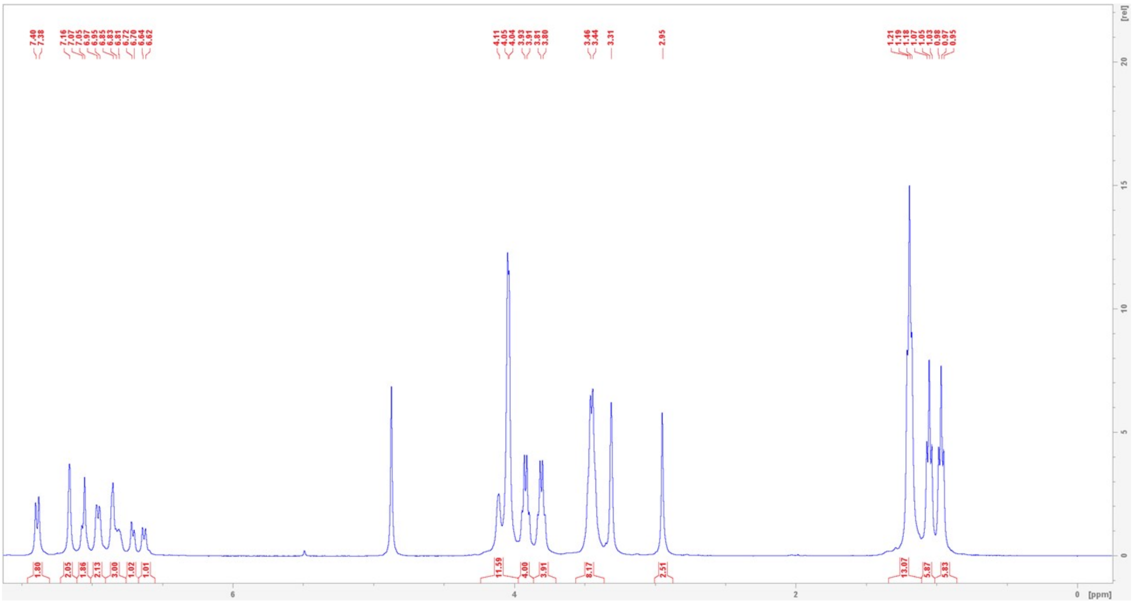

^{13}C:

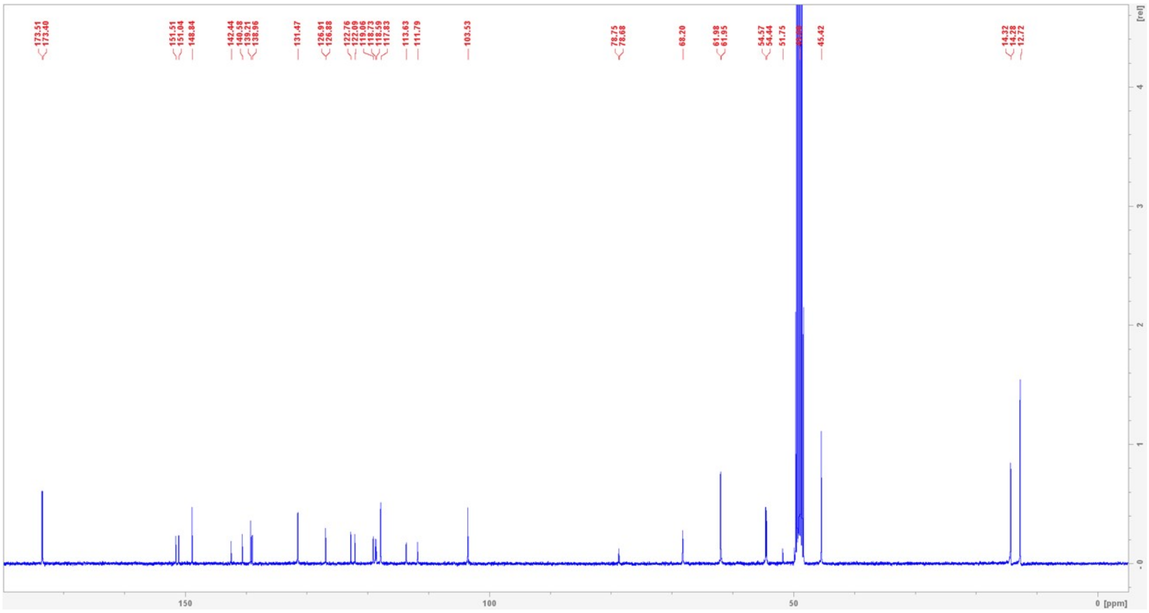

1:

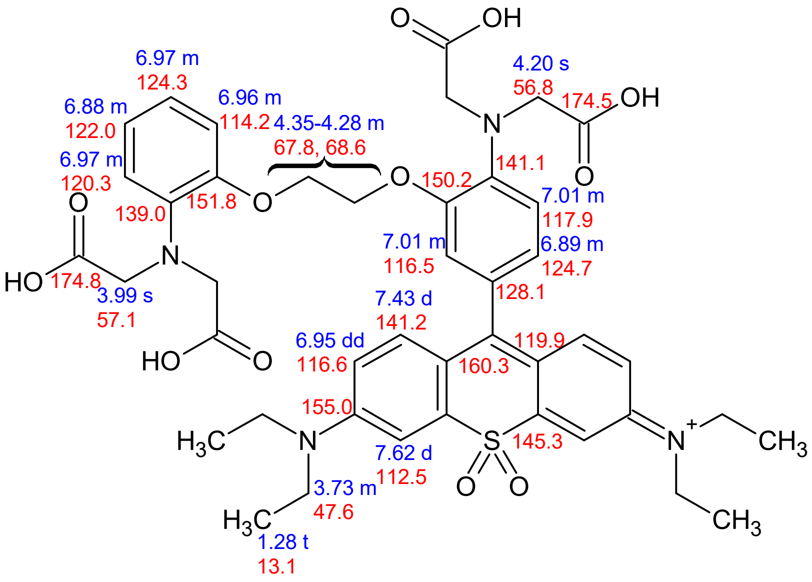

6.97 m
124.3
6.88 m
122.0
6.96 m
114.2
4.35-4.28 m
67.8, 68.6
4.20 s
56.8
174.5
OH
O
OH
6.97 m
120.3
O
139.0
151.8
O
O
150.2
141.1
7.01 m
117.9
HO
174.8
3.99 s
57.1
N
7.01 m
116.5
6.89 m
124.7
HO
O
7.43 d
141.2
128.1
6.95 dd
116.6
119.9
160.3
155.0
H3C
N
7.62 d
112.5
S
145.3
N+
CH3
3.73 m
47.6
O
O
H3C
1.28 t
13.1
CH3

^{1}H:

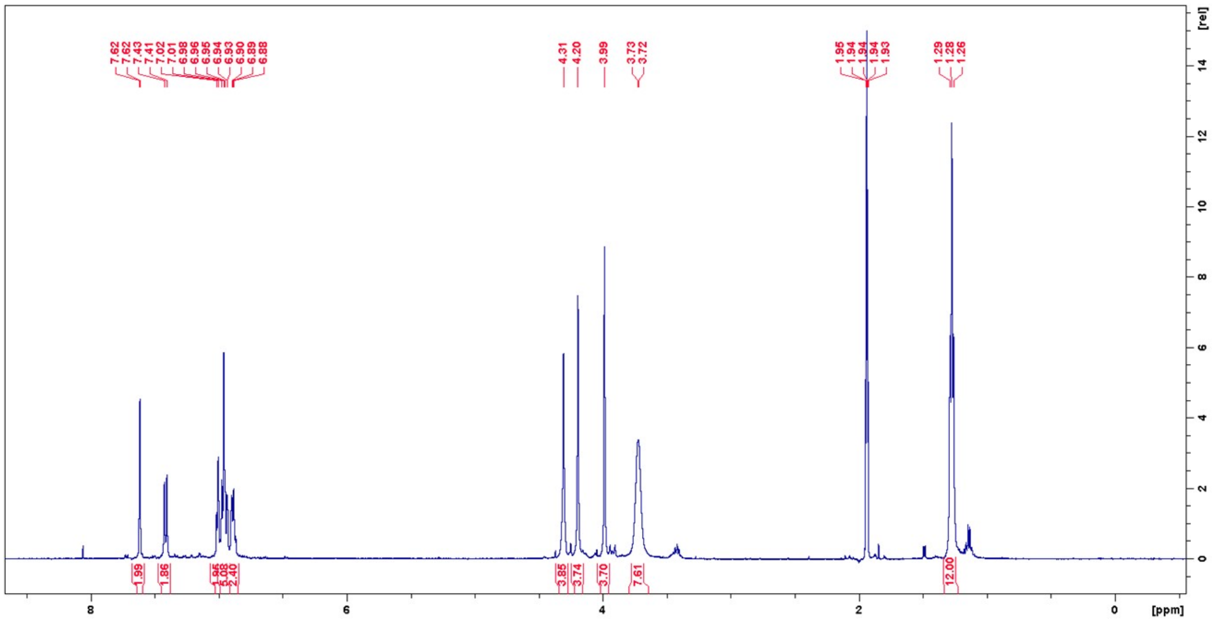

[rel]
[ppm]

[13]C:

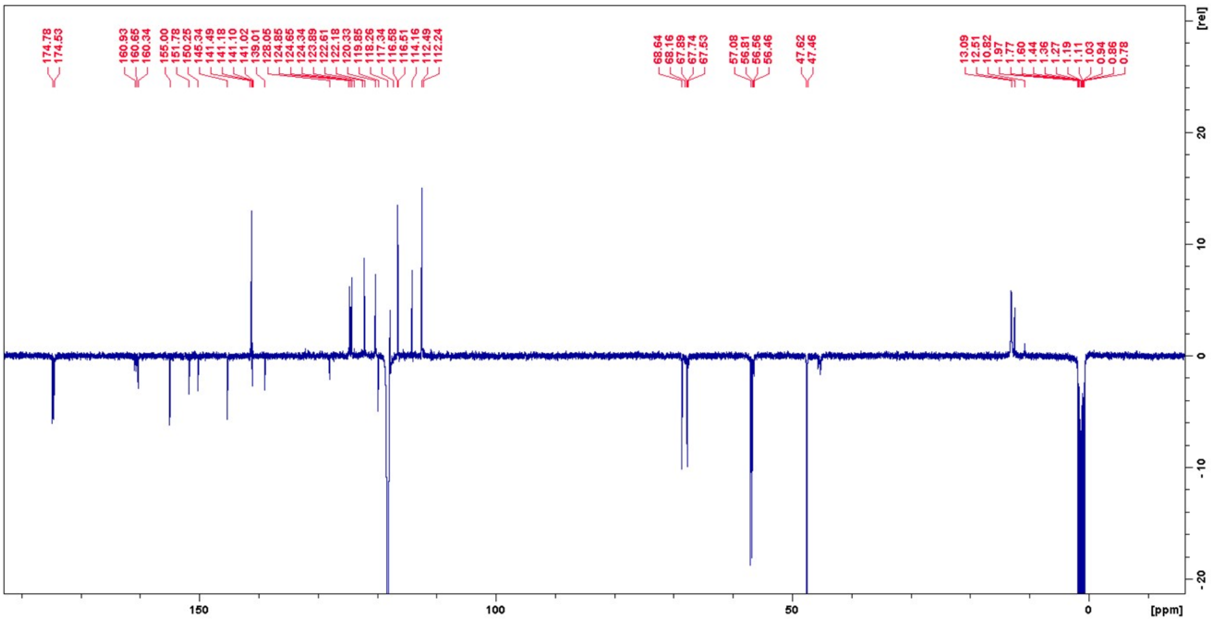

1:1 mixture of the synthesis product 1 and its -OCD_3 derivative formed by the NMR solvent during NMR sample preparation

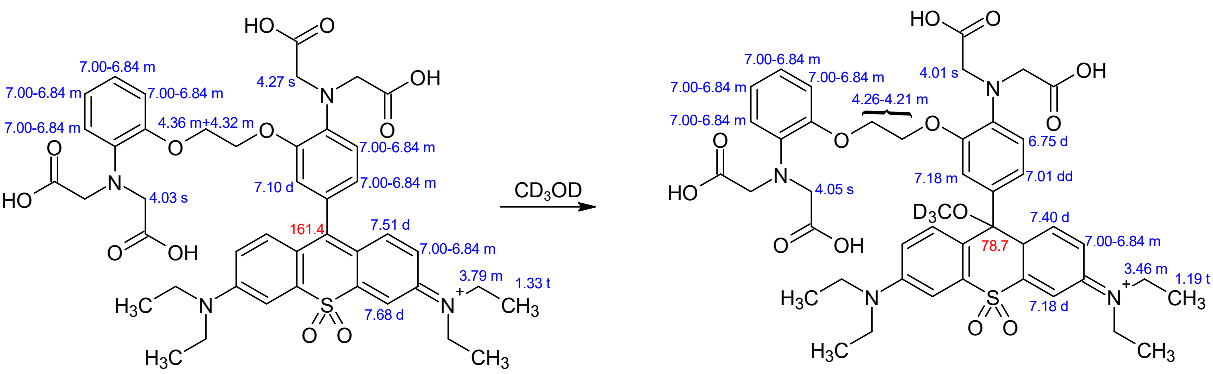

^1H NMR (500 MHz, CD_3OD) δ: 7.68 (d, *J*=2.7Hz, 2H), 7.51 (d, *J*=9.7Hz, 2H), 7.40 (d, *J*=9.0Hz, 2H), 7.18 (m, 1H), 7.18 (d, *J*=2.4Hz, 2H), 7.10 (d, *J*=1.8Hz, 1H), 7.01 (dd, *J*=8.5, 2.1Hz, 1H), 7.00-6.84 (m, 14H), 6.75 (d, *J*=8.5Hz, 1H), 4.36 (m, 2H), 4.32 (m, 2H), 4.27 (s, 4H), 4.26-4.21 (m, 4H), 4.05 (s, 4H), 4.03 (s, 4H), 4.01 (s, 4H), 3.79 (m, 8H), 3.46 (q, *J*=7.1Hz, 8H), 1.33 (t, *J*=6.8Hz, 12H), 1.19 (t, *J*=7.1Hz, 12H).

^{1}H: in CD_3OD

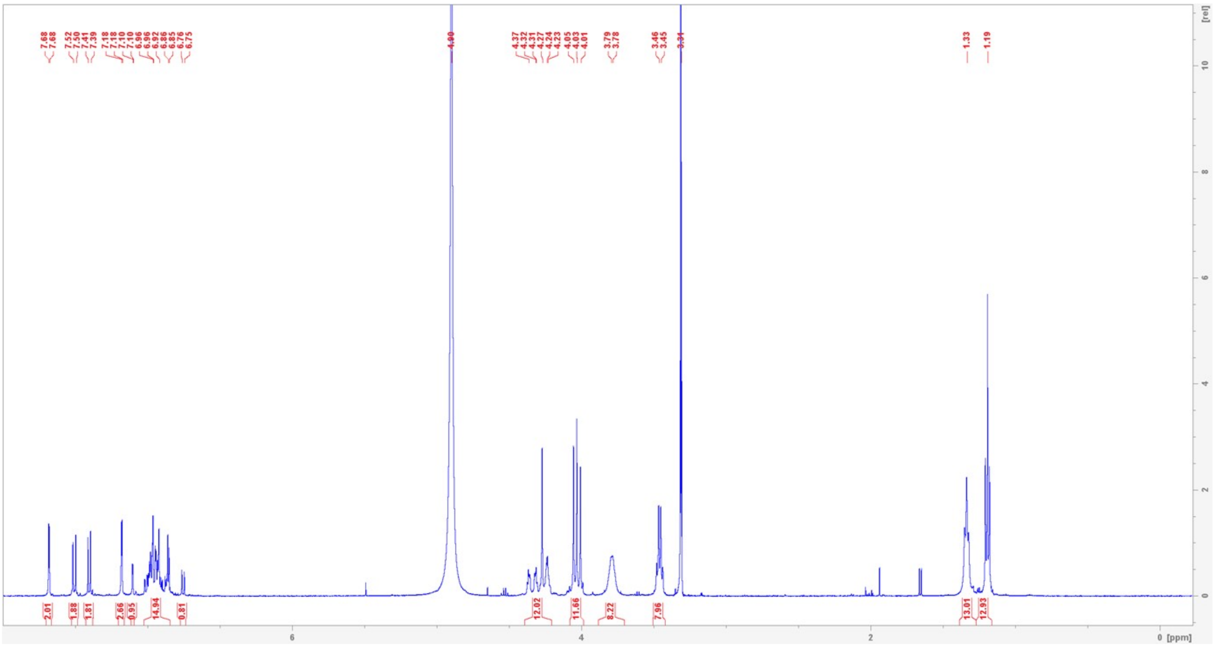

^{13}C: in CD_3OD

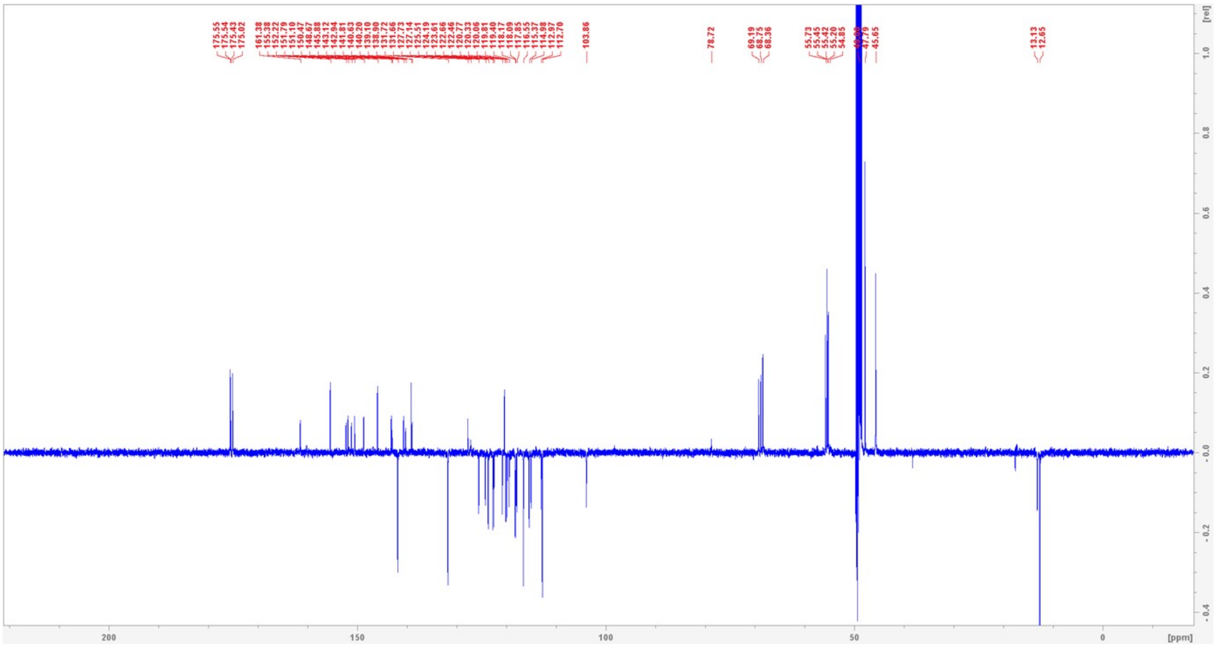

Solvent dependence of the DDQ mediated coupling

The formation of product **7a** in different solvents after 1h at room temperature, determined by Agilent 1100 HPLC:

Method #	Solvent	Starting material mg /1 ml	Product concentration	Theoretical product (mg)	Yield (%):
1	AcOH	37,34	33,23	49,29	67%
2	NMP	37,34	19,18	49,29	39%
3	DMF	37,28	25,23	49,21	51%
4	DMSO	37,34	24,55	49,29	50%
5	DCM	37,3	25,59	49,24	52%
6	MeOH	37,3	14,13	49,24	29%
7	EtOH	37,9	25,71	50,03	51%
8	Toluene	37,33	12,2	49,28	25%
9	HCOOH	37,3	7,43	49,24	15%
10	TFA	37,28	0,58	49,21	1%
11	ACN	37,24	9,1	49,16	19%
12	Pyridine	37,3	7,99	49,24	16%
13	AcOH/H2O 1/1	37,38	19,57	49,34	40%

Product **7a** purity:

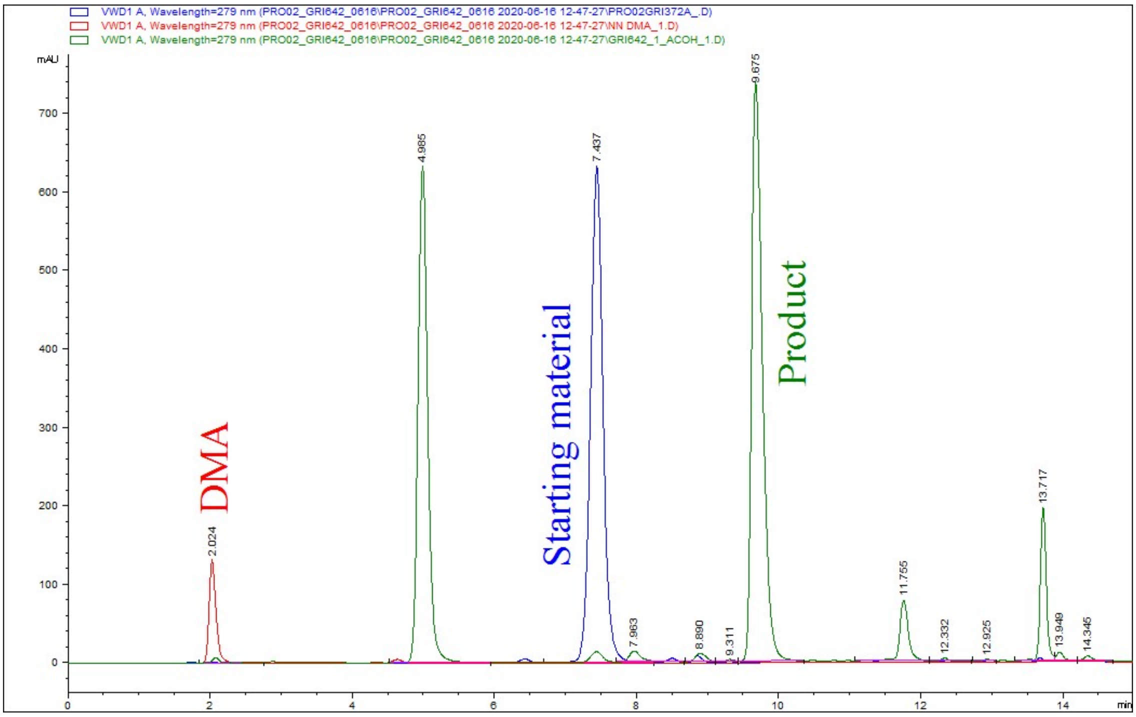

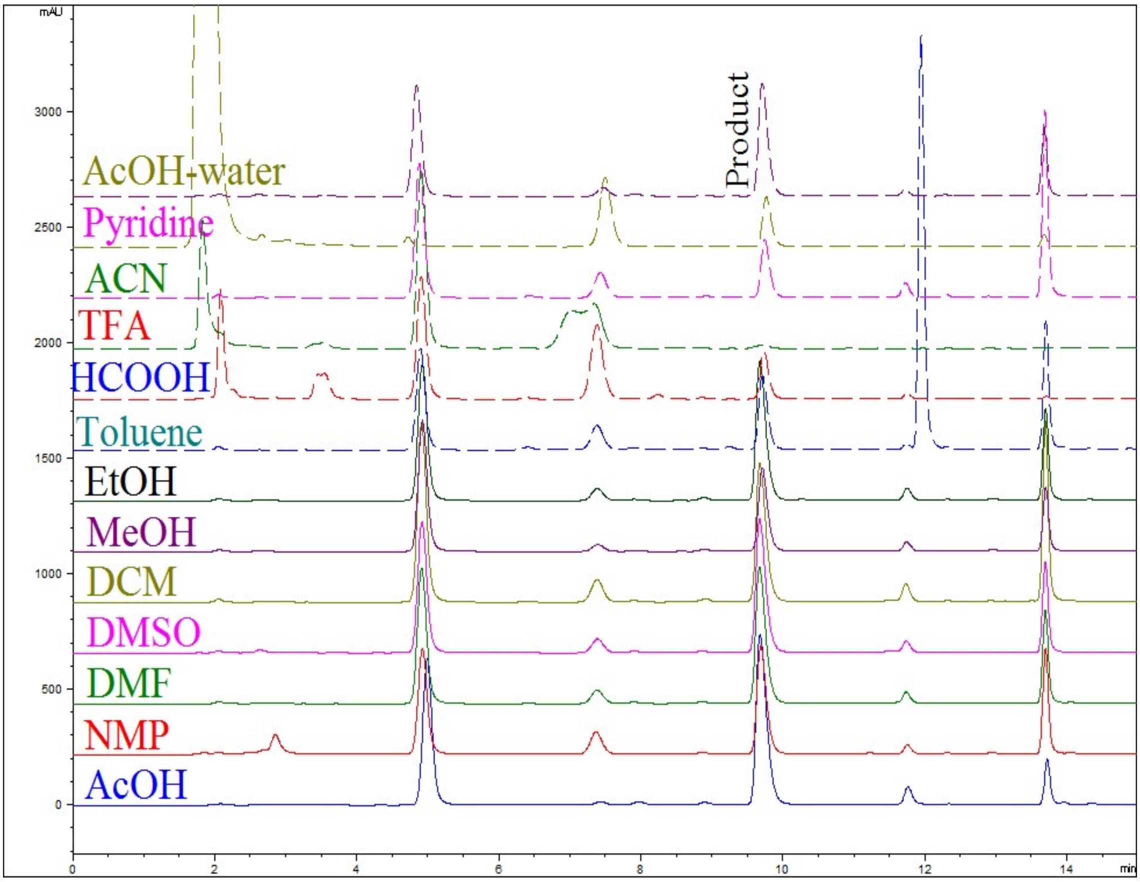

Determining Absorption and Emission maxima:

Solutions of metals were prepared in MOPS buffer by adding 0.2 mM metal ions to 30 mM MOPS and 100 mM KCl, solved in water. These metal solutions were used later on in every experiment. The pH of the MOPS buffer was set to 6.8.

Absorption measurement:

To determine the absorption peaks of the synthesized fluorescent dye, NanoDrop 1100 spectrophotometer (Thermo Scientific) was assessed. The fluorescent dye as well as equal amounts of fluorescent dye and metal ion solutions (Cu, Ni, Zn, Co, Mn, Mg, Fe and Ca) were mixed and measured. The relative intensity of the prepared solutions was measured in the range of 220-750 nm.

Emission measurement:

To determine the emission peaks of the synthesized fluorescent dye, NanoDrop 3300 fluorospectrometer (Thermo Scientific, Waltham, MA, US) was used. The fluorescent dye as well as equal amounts of fluorescent dye and metal ion solutions (Cu, Ni, Zn, Co, Mn, Mg, Fe and Ca) were mixed and measured. The relative fluorescent intensity of the prepared solutions was measured in the range of 500-750 nm.

Determining fluorescent signal increase (Qualitative):

The fluorescent labelled PBNPs were imaged using a two-dimensional epifluorescent optical imaging instrument. (FOBI™, Fluorescent Organism Bioimaging Instrument; Neoscience Co., Ltd., Suwon-si, Korea). Control sample of the fluorescent dye (0.5 mM) as well as metal ions (0.2 mM; Co, Zn, Sr, Cd, Mn, Hg, Cu, Fe, Pb, Mg, Ni, Ca and Ba) were mixed in equal volumes.

The images were collected immediately with excitation of 630–680 nm as well as 700-750 nm corresponding to the excitation maxima of the dye. The emission spectrum of the dye was in the pass band of the used emission filters for the Red as well as NIR images. Image acquisition parameters were the following: exposure time: 3000 msec and gain: 1. The images were evaluated with VivoQuant software (Invicro, 27 Drydock Avenue, Boston, MA, USA).

Quantifying fluorescent signal increase:

Previously observed metal ions were selected to determine the quantitative fluorescent signal increase of the Salamon-BAPTA dye. The selected metals were zinc, cadmium and calcium. 0.2 mM solutions of the dyes were prepared and later added to the 0.5 mM solution of the dye.

To quantify the fluorescent signal increase, synchronous scan was conducted by Edinburgh FLS 980 fluorescence spectrophotometer (Edinburgh Instruments, UK) in the range of 600-850 nm. A Xenon lamp source was used for excitation. The acquisition interval and the integration time were maintained at 3 nm and 0.1 s, respectively.

The synchronous fluorescence spectra were collected by simultaneously scanning the excitation and emission monochromator in the 600–850 nm range, with constant wavelength differences, Δλ, between them. Spectra were recorded for each sample, with Δλ of 40 nm. Fluorescence intensities were plotted as function of the excitation wavelength.

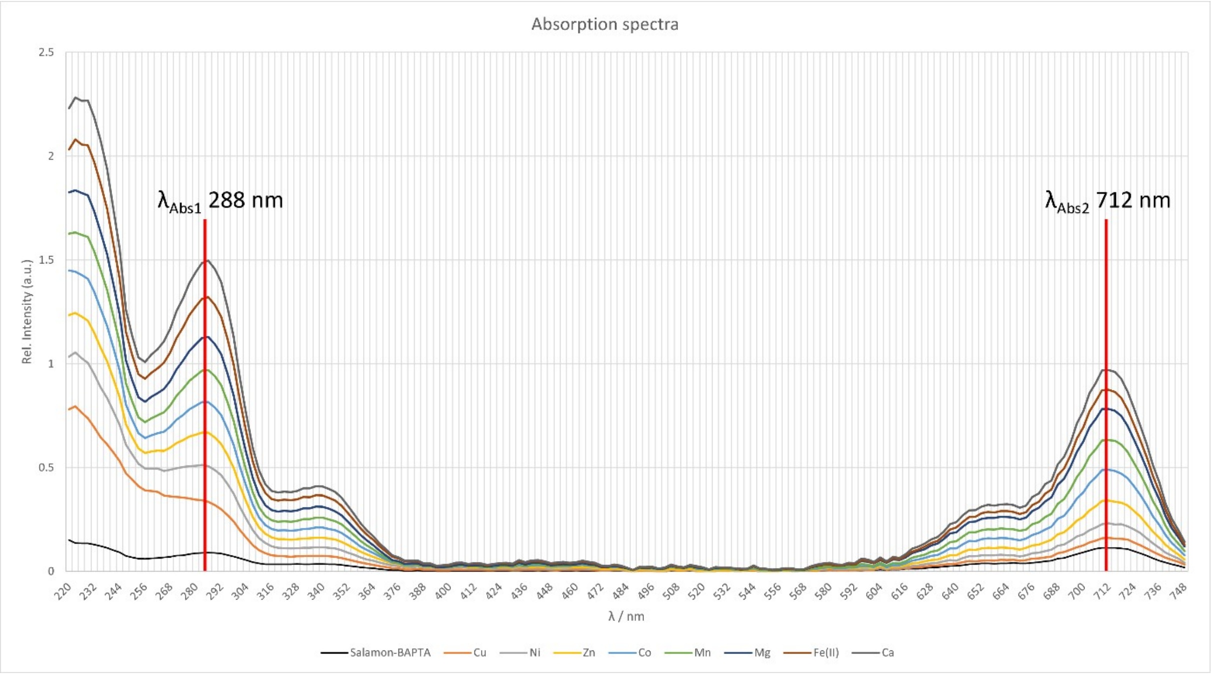

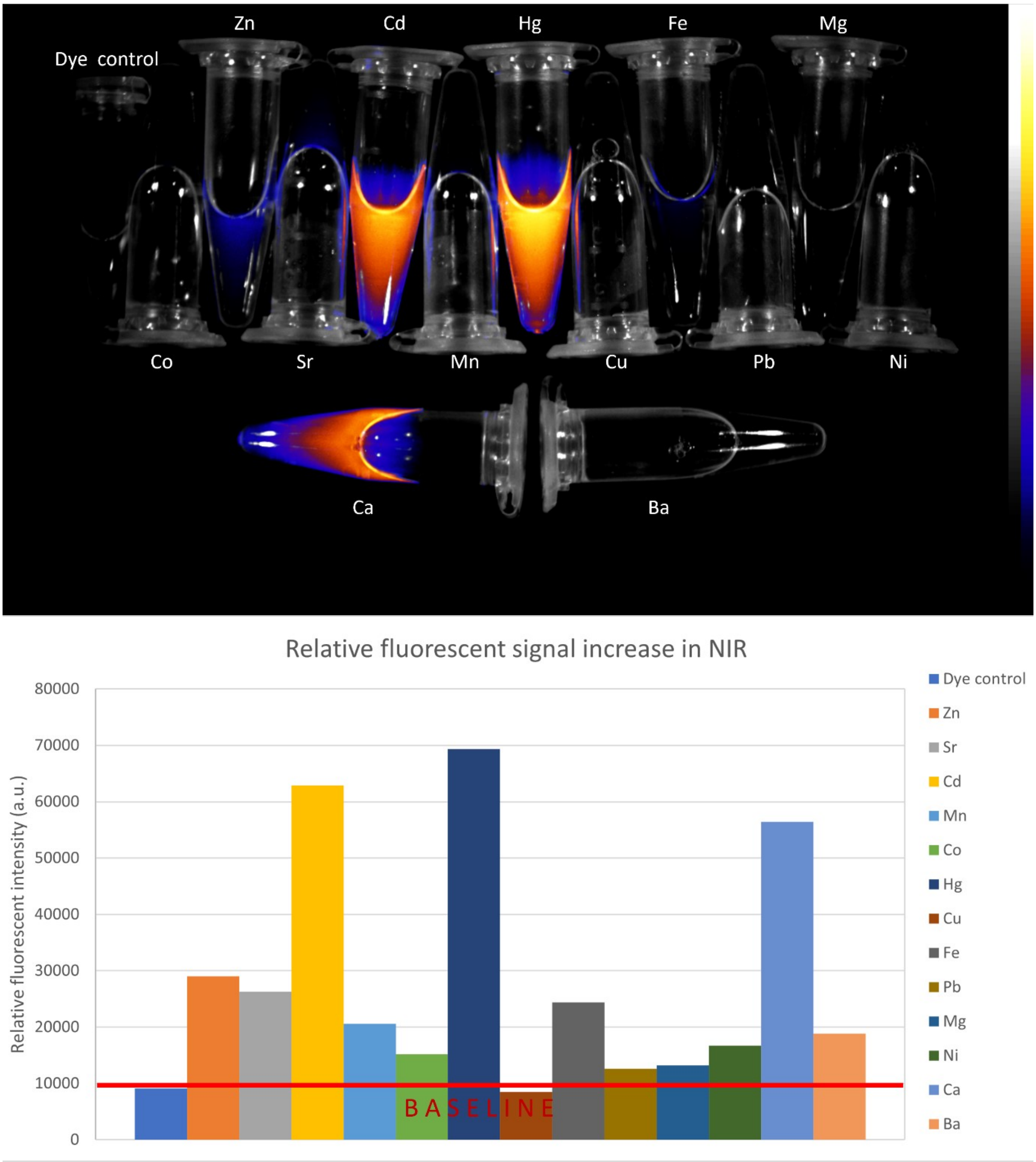
Dye control
Zn
Cd
Hg
Fe
Mg
Co
Sr
Mn
Cu
Pb
Ni
Ca
Ba
Relative fluorescent signal increase in NIR
Relative fluorescent intensity (a.u.)
80000
70000
60000
50000
40000
30000
20000
10000
0
BASELINE
Dye control
Zn
Sr
Cd
Mn
Co
Hg
Cu
Fe
Pb
Mg
Ni
Ca
Ba

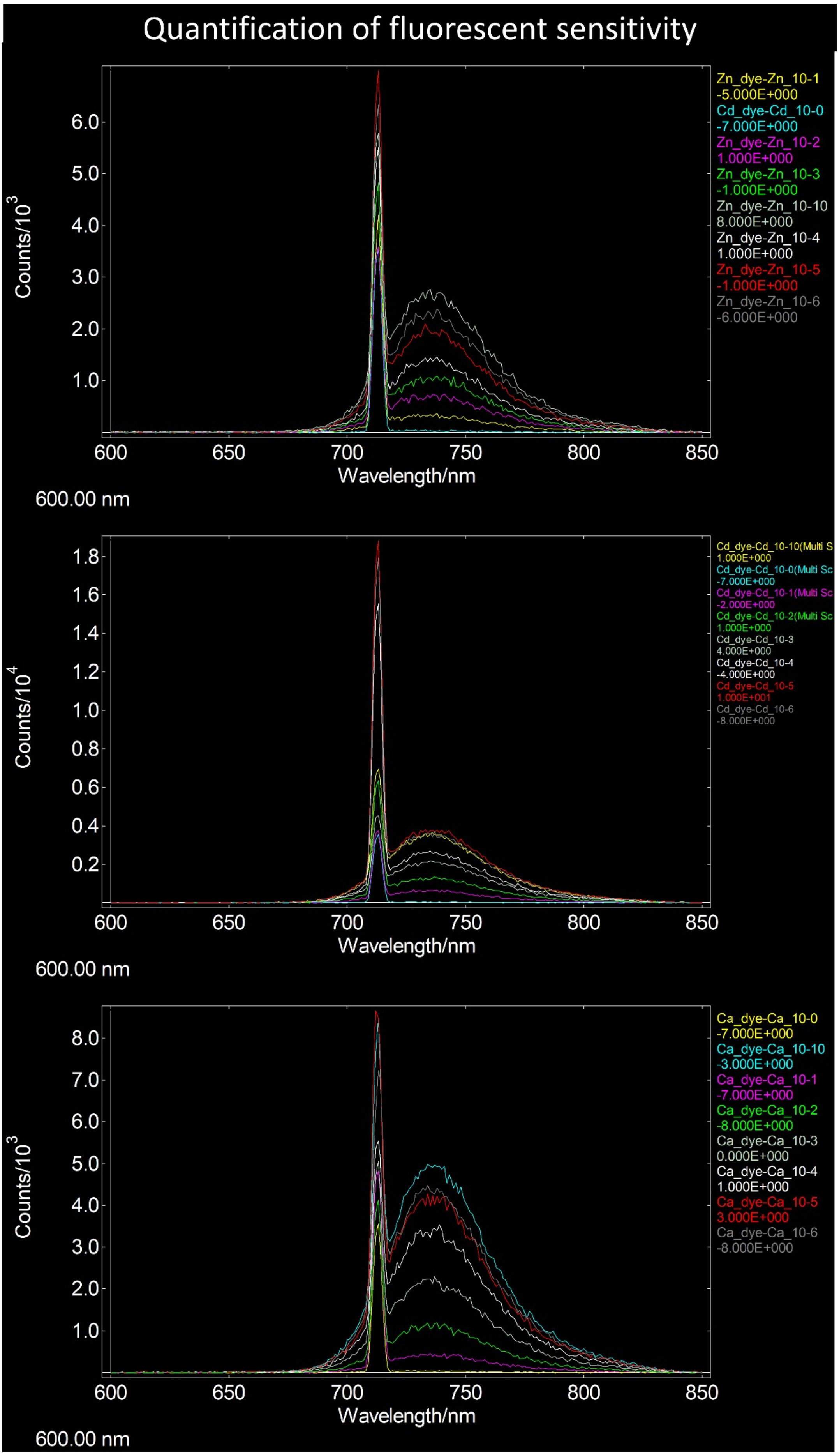
Quantification of fluorescent sensitivity
Counts/10^3
6.0
5.0
4.0
3.0
2.0
1.0
600
650
700
750
800
850
Wavelength/nm
600.00 nm
Zn_dye-Zn_10-1
-5.000E+000
Cd_dye-Cd_10-0
-7.000E+000
Zn_dye-Zn_10-2
1.000E+000
Zn_dye-Zn_10-3
-1.000E+000
Zn_dye-Zn_10-10
8.000E+000
Zn_dye-Zn_10-4
1.000E+000
Zn_dye-Zn_10-5
-1.000E+000
Zn_dye-Zn_10-6
-6.000E+000
Counts/10^4
1.8
1.6
1.4
1.2
1.0
0.8
0.6
0.4
0.2
600
650
700
750
800
850
Wavelength/nm
600.00 nm
Cd_dye-Cd_10-10(Multi S
1.000E+000
Cd_dye-Cd_10-0(Multi Sc
-7.000E+000
Cd_dye-Cd_10-1(Multi Sc
-2.000E+000
Cd_dye-Cd_10-2(Multi Sc
1.000E+000
Cd_dye-Cd_10-3
4.000E+000
Cd_dye-Cd_10-4
-4.000E+000
Cd_dye-Cd_10-5
1.000E+001
Cd_dye-Cd_10-6
-8.000E+000
Counts/10^3
8.0
7.0
6.0
5.0
4.0
3.0
2.0
1.0
600
650
700
750
800
850
Wavelength/nm
600.00 nm
Ca_dye-Ca_10-0
-7.000E+000
Ca_dye-Ca_10-10
-3.000E+000
Ca_dye-Ca_10-1
-7.000E+000
Ca_dye-Ca_10-2
-8.000E+000
Ca_dye-Ca_10-3
0.000E+000
Ca_dye-Ca_10-4
1.000E+000
Ca_dye-Ca_10-5
3.000E+000
Ca_dye-Ca_10-6
-8.000E+000

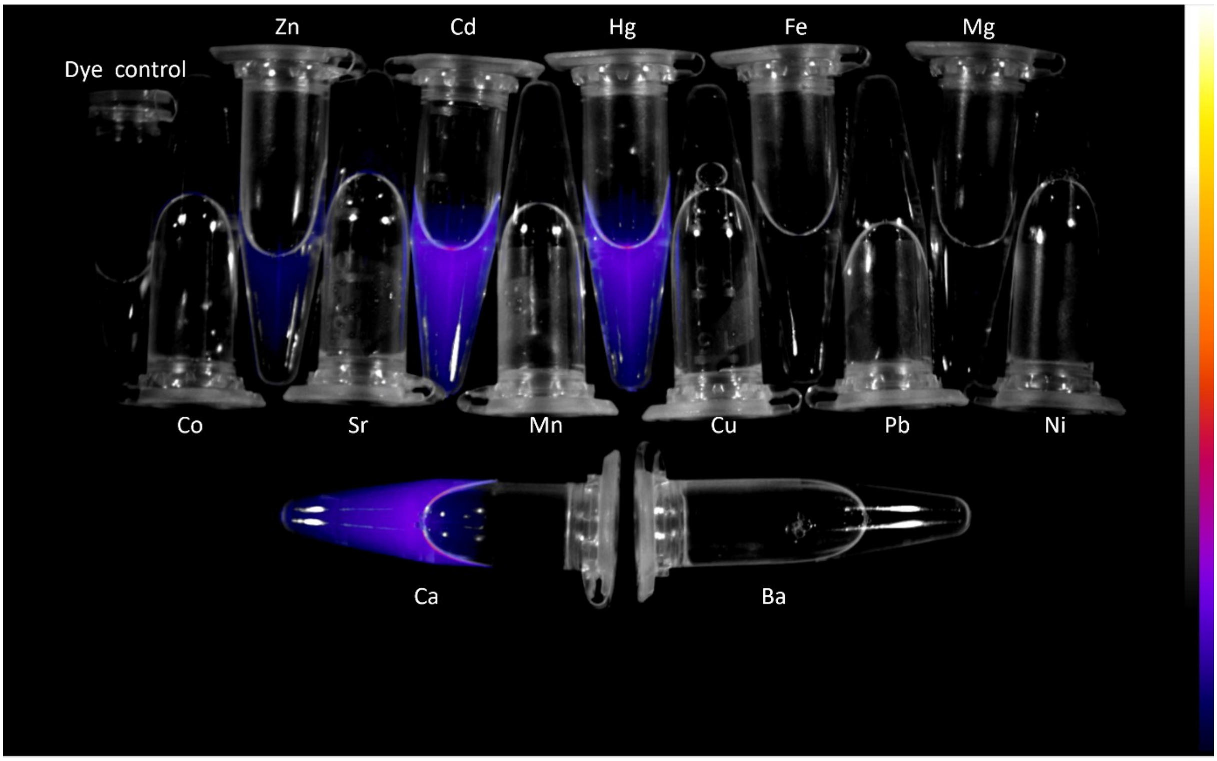

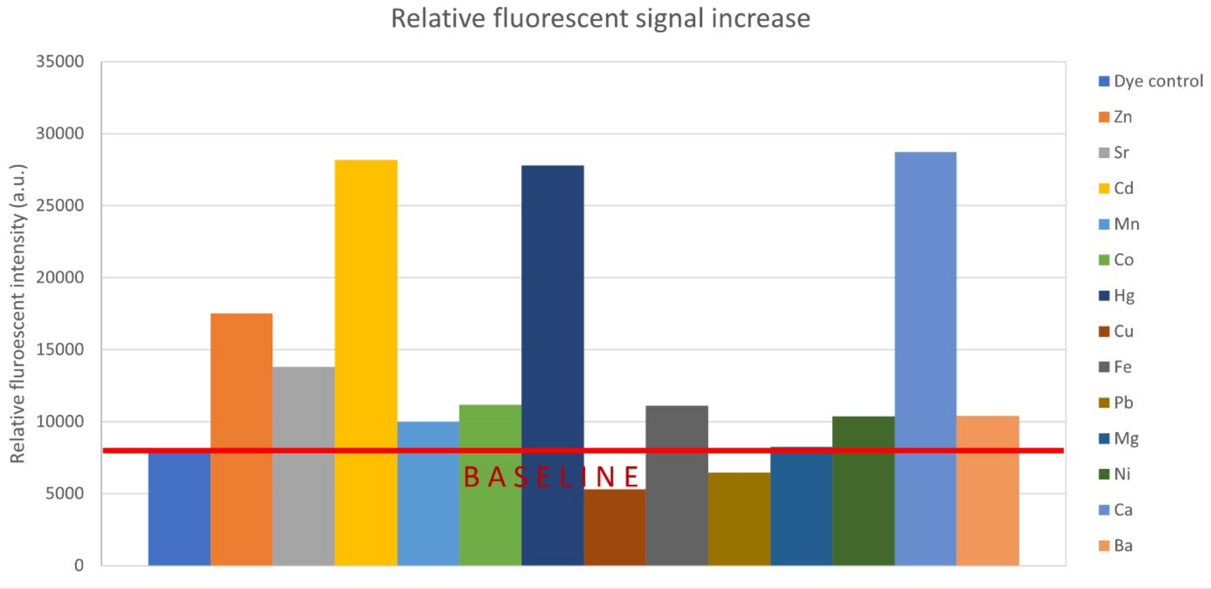

Molar Extinction Coefficient: The molar extinction coefficient (ε) of Solomon-red-BAPTA was determined by UV-Vis absorption spectroscopy using a NanoDrop 1100 spectrophotometer (Thermo Scientific) with a 1 mm path length. A concentration series of the dye (20, 40, 60, 80, and 100 µM) was prepared in 30 mM MOPS buffer (pH 6.8, 100 mM KCl). Absorbance values at the absorption maximum (λ = 711 nm) were plotted against concentration and the molar extinction coefficient was derived from the slope of the linear regression according to the Beer–Lambert law (A = ε·c·l), yielding ε = 67,600 $M^{-1}cm^{-1}$.

Fluorescence Quantum Yield: The fluorescence quantum yield (Φ) of Solomon-red-BAPTA was preliminarily estimated using the relative method with Rhodamine 6G in ethanol as the

reference standard (Φref = 0.95). Emission spectra were recorded on the Edinburgh FLS 980 fluorescence spectrophotometer under identical excitation wavelength, slit widths, and detector settings. The quantum yield was calculated using: $\Phi_{sample} = \Phi_{ref} \times (I_{sample}/I_{ref}) \times (A_{ref}/A_{sample}) \times (n_{sample}^2/n_{ref}^2)$, where I is the integrated emission intensity, A is the absorbance at the excitation wavelength, and n is the refractive index of the solvent. Due to the significant spectral mismatch between the reference and the dye (Δλem > 180 nm), the obtained value should be considered an approximation. Full spectroscopic characterization using a spectrally matched NIR reference standard is beyond the scope of the present work.

Fluorescence Lifetime Measurements: Fluorescence lifetime measurements were performed using the Edinburgh FLS 980 fluorescence spectrophotometer equipped with a time-correlated single photon counting (TCSPC) module. The dye was prepared from a 1 mM stock solution in acetonitrile, diluted 1:1 with 30 mM MOPS buffer (pH 6.8, 100 mM KCl) to yield a 50% v/v acetonitrile/water mixture. Ca^{2+}-free and Ca^{2+}-bound conditions were measured separately; Ca^{2+}-bound samples contained excess $CaCl_2$ (0.2 mM). Lifetime decays were fitted with mono- or bi-exponential decay models and the goodness of fit was assessed by the reduced chi-squared (χ^2) value. As the measurements were conducted in a mixed solvent system (50% MeCN/buffer), the reported lifetime values represent estimates under these specific conditions. Lifetime values in fully aqueous physiological media are expected to be somewhat lower due to increased non-radiative decay rates in water, and full characterization in aqueous conditions is beyond the scope of the present work.

pH Stability: The pH-dependent fluorescence stability of Solomon-red-BAPTA was assessed using the NanoDrop 1100 spectrophotometer. The dye (100 µM) was prepared in the following buffer systems: MES buffer at pH 5.5 and 6.5, HEPES buffer at pH 7.4, and Tris-HCl buffer at pH 8.5 and 9.6, each at 30 mM ionic strength with 100 mM KCl. Absorbance at the absorption maximum (711 nm) was recorded for each condition to define the operational pH stability window of the sensor.

Selectivity in the Presence of Competing Metal Ions: The selectivity of Solomon-red-BAPTA toward Ca^{2+} in the presence of potentially interfering divalent metal ions was evaluated using a NanoDrop 3300 fluorospectrometer (Thermo Scientific). Due to the instrument's LED configuration, samples were excited using the blue LED source (~470 nm) with emission recorded at 736 nm. The dye (0.5 mM final concentration) was prepared by diluting the 1 mM acetonitrile stock 1:1 with 30 mM MOPS buffer (pH 6.8, 100 mM KCl). Ca^{2+} was added at 0.2 mM and competing metal ions (Zn^{2+} as $ZnCl_2$, Cd^{2+} as cadmium acetate) at 0.02 mM (1/10th of the Ca^{2+} concentration), from 1 mM aqueous stock solutions prepared in MOPS buffer. Seven sample compositions were measured in triplicate: dye alone, dye + Ca^{2+}, dye + Zn^{2+}, dye + Cd^{2+}, dye + Ca^{2+} + Zn^{2+}, dye + Ca^{2+} + Cd^{2+}, and dye + Ca^{2+} + Zn^{2+} + Cd^{2+}. Buffer alone served as the instrument blank. Fluorescence intensity at 736 nm was reported as mean ± SD of triplicate measurements.

RESULTS

Spectroscopic characterization: Solomon-red-BAPTA exhibited an absorption maximum at 711 nm and an emission maximum at 736 nm, providing a Stokes shift of 24 nm sufficient to minimize detection-side light scattering. The molar extinction coefficient at the absorption maximum was determined to be ε = 67,600 $M^{-1}cm^{-1}$, confirming strong NIR absorption. A preliminary estimate of the Ca^{2+}-saturated fluorescence quantum yield yielded Φsat ≈ 0.25, though this value carries inherent uncertainty due to the large spectral mismatch with the Rhodamine 6G reference standard; comprehensive photophysical characterization with an appropriate NIR reference is warranted in future work.

Conc (µM)	Conc (M)	Abs1	Abs2	Abs3	Abs4	Abs5	Used1	Used2	Used3	Average A
20	2,00E-05	0.370	0.354	0.375			0.370	0.354	0.375	0,37
40	4,00E-05	0.502	0.534	0.515			0.502	0.534	0.515	0,52
60	6,00E-05	0.679	0.642	0.607	0.634	0.644	0.642	0.634	0.644	0,64
80	8,00E-05	0.785	0.845	0.846	0.715	0.788	0.785	0.788	0.715	0,76
100	1,00E-04	0.916	0.915	0.927			0.916	0.915	0.927	0,92

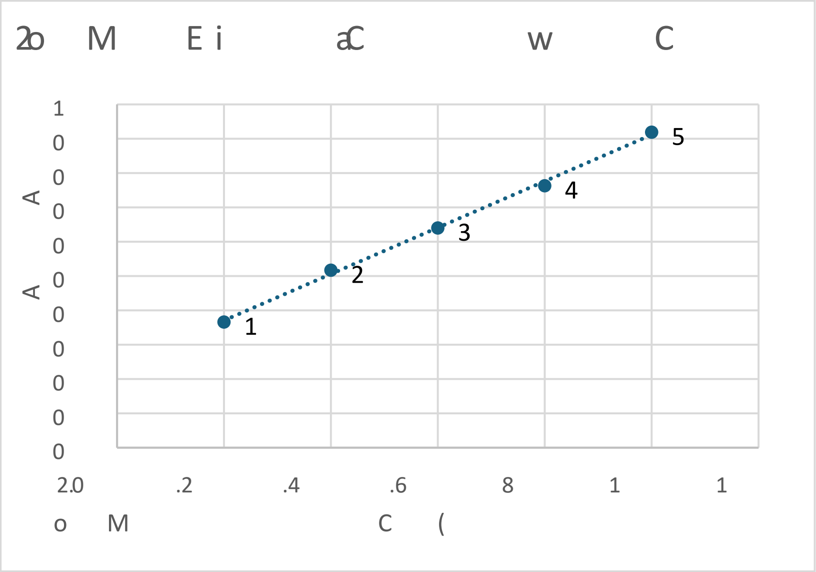

slope	6760
path length	0,1 cm
ε	67600 $M^{-1}cm^{-1}$
λ	711 nm

Fluorescence lifetime: Time-correlated single photon counting measurements in a 50% MeCN/MOPS buffer mixture revealed Ca^{2+}-free and Ca^{2+}-bound fluorescence lifetimes of approximately $\tau_{free} \approx 0.5$ ns and $\tau_{bound} \approx 1.8$ ns, respectively, representing an approximately 3.6-fold increase upon Ca^{2+} binding consistent with PET-based quenching by the free BAPTA chelator. These values are estimates under the mixed solvent conditions used and are expected to be somewhat lower in fully aqueous media.

pH stability: The fluorescence response of Solomon-red-BAPTA was stable across a physiologically relevant pH range. Consistent absorption at 711 nm was maintained between pH 6.5 and 8.5, encompassing the pH range of most biological compartments. Reduced stability was observed at pH 5.5 and 9.6, indicating the sensor is best suited for near-neutral to mildly alkaline conditions.

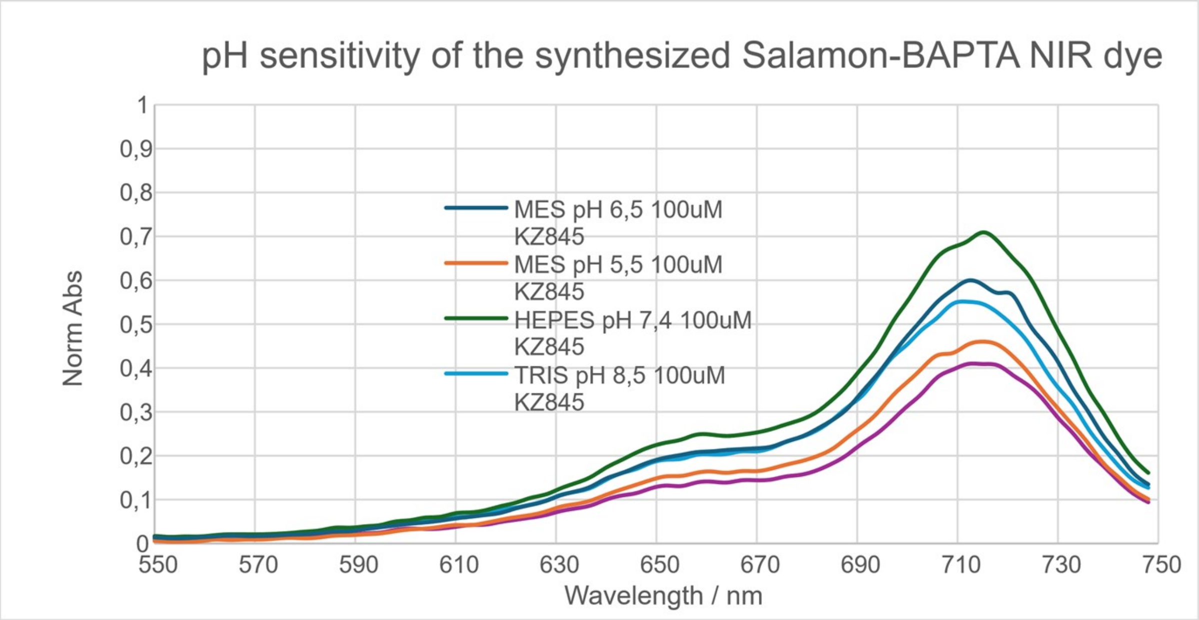

Metal ion selectivity: The dye alone produced a baseline fluorescence of 168.5 ± 12.3 RFU at 736 nm. Addition of Ca^{2+} (0.2 mM) increased the signal 5.7-fold (966.3 ± 78.6 RFU). At 1/10th the Ca^{2+} concentration (0.02 mM), neither Zn^{2+} (240.6 ± 73.5 RFU, 1.4-fold) nor Cd^{2+} (177.6 ± 6.1 RFU, 1.1-fold) alone produced significant fluorescence enhancement. The Ca^{2+}-induced signal was fully preserved in the presence of Zn^{2+} (1091.1 ± 156.0 RFU, 6.5-fold) and in the presence of Cd^{2+} (1196.2 ± 12.8 RFU, 7.1-fold), and remained dominant in the ternary mixture of all three ions (967.5 ± 56.8 RFU, 5.7-fold). These results demonstrate that Solomon-red-BAPTA maintains reliable Ca^{2+} selectivity in the presence of Zn^{2+} and Cd^{2+} at physiologically relevant concentration ratios.

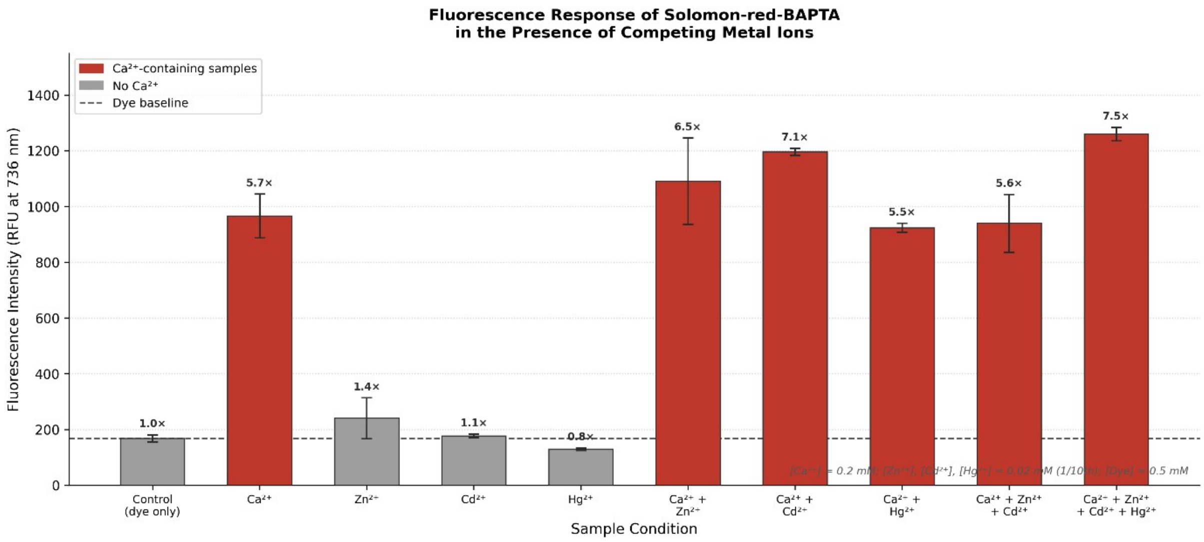

Hg^{2+} alone at 0.02 mM produced a fluorescence intensity of 130.1 ± 4.6 RFU, slightly below the dye baseline (168.5 ± 12.3 RFU), consistent with mild fluorescence quenching via the heavy atom effect. The Ca^{2+}-induced signal was preserved in the presence of Hg^{2+} (924.0 ± 16.5 RFU, 5.5-fold), and remained elevated in the quaternary mixture of all three competing ions (1259.9 ± 24.3 RFU, 7.5-fold). S8–S10 were measured in a separate session using an aged dye stock; no correction factor was applied.

[i] Sanjay K. Srivastava , Prem Man Singh Chauhan & Amiya P. Bhaduri (1999) A Novel Strategy for N-Alkylation of Primary Amines, Synthetic Communications, 29:12, 2085-2091, DOI: 10.1080/00397919908086201

[ii] Yinfeng Xu, Tiantian Cong, Ping Liuab & Peipei Sun, Highly regioselective para-methylthiolation/bridging methylenation of arylamines promoted by NH4I, Org. Biomol. Chem., 2015,13, 9742-9745, DOI: 10.1039/C5OB01679G

[iii] Shubhra B. Maity, Saikat Banerjee, Kyoung Sunwoo, Jong Seung Kim & Parimal K. Bharadwaj, A Fluorescent Chemosensor for Hg 2+ and Cd 2+ Ions in Aqueous Medium under Physiological pH and Its Applications in Imaging Living Cells, Inorganic Chemistry, 2015, 54, (8), 3929-3936, DOI:10.1021/acs.inorgchem.5b00106

[iv] Shubhra B. Maity, Saikat Banerjee, Kyoung Sunwoo, Jong Seung Kim & Parimal K. Bharadwaj, A Fluorescent Chemosensor for Hg 2+ and Cd 2+ Ions in Aqueous Medium under Physiological pH and Its Applications in Imaging Living Cells, Inorganic Chemistry, 2015, 54, (8), 3929-3936, DOI: 10.1021/acs.inorgchem.5b00106

[v] Zhichao Liu, Xia Jing, Sanjun Zhang, & Yang Tian, A Copper Nanocluster-Based Fluorescent Probe for Real-Time Imaging and Ratiometric Biosensing of Calcium Ions in Neurons, Analytical Chemistry, 2019, 91, (3), 2488-2497, DOI: 10.1021/acs.analchem.8b05360
